# Analysis of transcriptomic features reveals molecular endotypes of SLE with clinical implications

**DOI:** 10.1186/s13073-023-01237-9

**Published:** 2023-10-16

**Authors:** Erika L. Hubbard, Prathyusha Bachali, Kathryn M. Kingsmore, Yisha He, Michelle D. Catalina, Amrie C. Grammer, Peter E. Lipsky

**Affiliations:** 1 AMPEL BioSolutions, LLC, 250 W. Main St. #300, Charlottesville, VA 22902 USA; 2 RILITE Research Institute, Charlottesville, VA 22902 USA; 3 Altria, Richmond, VA 23230 USA; 4 AbbVie, Worcester, MA 01605 USA

**Keywords:** Systemic lupus erythematosus (SLE), Autoimmunity, Inflammation, Gene expression, Endotype, Machine learning (ML)

## Abstract

**Background:**

Systemic lupus erythematosus (SLE) is known to be clinically heterogeneous. Previous efforts to characterize subsets of SLE patients based on gene expression analysis have not been reproduced because of small sample sizes or technical problems. The aim of this study was to develop a robust patient stratification system using gene expression profiling to characterize individual lupus patients.

**Methods:**

We employed gene set variation analysis (GSVA) of informative gene modules to identify molecular endotypes of SLE patients, machine learning (ML) to classify individual patients into molecular subsets, and logistic regression to develop a composite metric estimating the scope of immunologic perturbations. SHapley Additive ExPlanations (SHAP) revealed the impact of specific features on patient sub-setting.

**Results:**

Using five datasets comprising 2183 patients, eight SLE endotypes were identified. Expanded analysis of 3166 samples in 17 datasets revealed that each endotype had unique gene enrichment patterns, but not all endotypes were observed in all datasets. ML algorithms trained on 2183 patients and tested on 983 patients not used to develop the model demonstrated effective classification into one of eight endotypes. SHAP indicated a unique array of features influential in sorting individual samples into each of the endotypes. A composite molecular score was calculated for each patient and significantly correlated with standard laboratory measures. Significant differences in clinical characteristics were associated with different endotypes, with those with the least perturbed transcriptional profile manifesting lower disease severity. The more abnormal endotypes were significantly more likely to experience a severe flare over the subsequent 52 weeks while on standard-of-care medication and specific endotypes were more likely to be clinical responders to the investigational product tested in one clinical trial analyzed (tabalumab).

**Conclusions:**

Transcriptomic profiling and ML reproducibly separated lupus patients into molecular endotypes with significant differences in clinical features, outcomes, and responsiveness to therapy. Our classification approach using a composite scoring system based on underlying molecular abnormalities has both staging and prognostic relevance.

**Supplementary Information:**

The online version contains supplementary material available at 10.1186/s13073-023-01237-9.

## Background

The absence of a typical disease pattern is a major limiting feature in understanding the pathogenesis of and developing more effective therapies for systemic lupus erythematosus (SLE, lupus) [1, 2]. Therefore, efforts have been undertaken to identify molecular endotypes of SLE, that is, subsets of patients defined by distinct pathobiological functions, biomarkers, or other disease mechanisms.

Determination of endotypes has already been employed in the management of cancer and allergic disease and is just beginning to be conceptualized in other autoimmune conditions [3–8]. One goal of endotyping of lupus is to identify groups of patients expected to be more likely to respond to specific treatments, thereby increasing the likelihood of success of clinical care and clinical trials [9, 10]. We and others have found that heterogeneity in lupus can manifest at the level of gene expression in peripheral blood [9, 11–14], suggesting that molecular profiling might serve as the basis of identifying specific lupus endotypes with clinical implications.

To date, several groups have reported subclassifications of SLE based on transcriptomics [9, 10, 12, 15–21], but, in general, these have been single-center studies of limited numbers of patients and a broad consensus of recognized subsets has not emerged. Moreover, these studies have not considered confounding variables on gene expression, such as ancestry or medication, nor have the findings been translated into clinical care or clinical trial design [13, 22]. Here, we present an approach to identify molecular endotypes of lupus patients based on transcriptomic profiling employing 32 immune and inflammatory-related features. We leverage transcriptomic data, machine learning (ML), and contemporary bioinformatics to subclassify 3166 lupus patients into eight endotypes and develop an accompanying clinical metric, the *Lu*pus *C*ell and *I*mmune *S*core (LuCIS), to estimate a patient’s lupus-related immunologic activity. To our knowledge, this is the first transcriptomic-based molecular endotyping approach that has staging and prognostic implications. Translation of these findings into the clinic may serve to facilitate personalized medicine.

## Methods

### Patient involvement

Patients were not directly recruited or involved in this study.

### Datasets

A total of 17 transcriptomic lupus datasets were utilized in this study (Table 1, Additional file 1: Table S1). These datasets were derived from both clinical trials and registries, and in most, the American College of Rheumatology (ACR) classification criteria for SLE were used with varying clinical data available. The datasets selected for initial validation and training had more extensive clinical metadata.

**Table 1:** Condensed summary of transcriptional whole blood lupus datasets

Dataset	Accession number	Technology	SLE Classification Criteria	Clinical metadata	Clinical trial	No. lupus samples analyzed
1	GSE88884 (ILL-1)	Microarray	ACR	Extensive	NCT01196091	813 baseline active female SLE
2	GSE88884 (ILL-2)	Microarray	ACR	Extensive	NCT01205438	807 baseline active female SLE
3	GSE45291	Microarray	ACR or SLICC	Extensive	None	266 SLE
4	GSE65391	Microarray	Not specified	Extensive	None	137 baseline pediatric SLE
5	GSE116006	RNA-seq	ACR	Age, gender, IFN status, drug exposure	NCT01405196	160 SLE
6	GSE22098	Microarray	Not specified	Age, gender, ancestry	None	28 adult SLE
7	GSE29536	Microarray	Not specified	None	None	27 SLE
8	GSE39088	Microarray	ACR	Age, gender, ancestry	None	21 SLE
9	GSE49454	Microarray	ACR	Extensive	NCT00920114	49 SLE
10	GSE61635	Microarray	Not specified	None	None	64 SLE
11	GSE72509	RNA-seq	Not specified	Gender, ancestry, Anti-Ro60 status, ISM status	None	99 SLE
12	GSE72747	Microarray	ACR	Age, gender, SLEDAI, GFR, dsDNA titer	NCT01058343	10 baseline SLE
13	GSE110174	Microarray	Not specified	Absolute neutrophils, steroid use	NCT00119678	144 SLE
14	GSE112087	RNA-seq	ACR	Age, gender, ancestry	None	29 female SLE
15	GSE138458	Microarray	ACR	High/low SLEDAI	None	307 SLE
16	GSE185047	Microarray	ACR	None	NCT02665364	177 baseline SLE
17	SRX10438277 (PRJNA717024)	RNA-seq	Not specified	Extensive	None	28 SLE

*ACR* American College of Rheumatology, *SLEDAI* Systemic Lupus Erythematosus Disease Activity Index, *SLICC* Systemic Lupus International Collaborating Clinics, *RNA-seq* RNA sequencing, *IFN* interferon, *ISM* interferon signature metric, *GFR* glomerular filtration rate

### Normalization of raw data files

#### Microarray data (Affymetrix and Illumina)

Raw data of each transcriptomic dataset was downloaded from Gene Expression Omnibus (GEO). All statistical analysis was conducted using R v. 4.0.4 and relevant Bioconductor packages. To inspect raw data files for outliers, PCA plots were generated for each dataset. Datasets culled of outliers were cleaned of background noise and normalized using either Robust Multiarray Average (RMA), GCRMA, or normexp background correction (NEQC), based on the microarray platform resulting in log2-transformed expression values into R expression set objects (e-sets). Analysis was conducted using normalized datasets prepared using both standard Affymetrix chip definition files (CDF), as well as custom made BrainArray CDFs. Illumina CDFs were used for the Illumina datasets.

#### RNA-sequencing (RNA-seq) data

Raw data files were downloaded from NCBI Sequence Read Archive (SRA) website using SRA toolkit (v. 2.10) and converted to FASTQ files using fastq dump. Quality of the FASTQ files was checked using FASTQC software (v. 0.11.9). Adapters and bad-quality reads were trimmed using Trimmomatic software (Unix-based tool v. 0.38). Good quality reads were aligned to the human reference genome (hg38) using STAR aligner (v. 2.7). STAR-aligned reads were saved as.sam files and were converted to.bam files using sambamba (v. 0.8). Read counts were summarized using the featureCounts function of the Subread R package (v. 1.61). Count normalization and log transformation were carried out using the DESeq2 (v. 1.32) R package.

Batch effects were examined in each individual dataset by PCA. No batch effects were detected and no corrections were made.

### Gene set variation analysis (GSVA)

GSVA is a non-parametric, unsupervised statistical method for estimating the variation of pre-defined gene sets in samples of transcriptomic expression datasets [23] GSVA works by transforming a gene matrix (gene-by-sample) into a gene set-by-sample matrix resulting in an enrichment score for each sample and pre-defined gene set. The inputs for the GSVA algorithm were a matrix of log2 expression values and a collection of pre-defined gene sets. Enrichment scores (GSVA scores) were calculated non-parametrically using a Kolmogorov-Smirnoff (KS)-like random walk statistic. The enrichment scores are the difference between the largest positive and negative random walk deviation from zero, respectively, for a given sample and gene set.

The enrichment scores take on values between − 1 and 1, where 1 represents enrichment of every gene in a particular gene set among the samples analyzed compared to every other gene not included in the specified gene set, whereas − 1 represents a relative lack of enrichment. Each gene in a gene set is given a rank based on expression values and the KS-like random walk statistic is calculated.

Out of 134 previously published gene sets and unpublished gene sets developed to represent immune cell signatures subsequently validated with flow cytometry [13, 24–27], 72 were used for GSVA after removing tissue-specific and redundant gene sets.

Calculated GSVA enrichment scores for the 32 gene sets in each patient were used as input to *k*-means clustering for determination of subsets. GSVA v. 1.38.2 was carried out in R v. 4.0.4.

### K-means clustering

Unsupervised algorithms like clustering were carried out to classify the lupus samples into subgroups/clusters of varying immunologic/inflammatory activity. Baseline GSVA enrichment scores of the 32 derived molecular signatures in two combined datasets, GSE88884 (ILL-1 and ILL-2), were used as input into five clustering algorithms: *k*-means, hierarchical, self-organizing maps, spectral, and Gaussian mixture modeling. These datasets were chosen for preliminary proof-of-concept because of the large number of samples and extensive associated clinical metadata. The resulting clusters from these methods were compared to one another by Adjusted Rand Index and silhouette scores were computed for each method (Additional file 1: Table S2). *K*-means clustering with the most stable clusters identified after 5000 iterations and minimal loss function was selected as the algorithm of choice based on having the highest silhouette score. The number of *k* clusters for each dataset was determined by elbow and silhouette plots (Additional file 2: Figure S1). KElbow Visualizer from the yellowbrick module in Python was used to determine the elbow of the plot. Briefly, the elbow is the cutoff point of diminishing returns and can be used to identify the optimal number of clusters. Silhouette analysis also informed how well-defined and well-separated clusters were, with a score of 1 representing perfect distinguishment (best) and -1 representing poor distinguishment (worst). Comparison of clusters from different datasets were compared by cosine similarity using the “lsa” R package [28]. Cosine similarity > 0.7 was considered highly similar.

*K*-means clustering was conducted using the scikit-learn (v. 0.24.1) Python library. The chosen parameters for the *k*-means algorithm were as follows: number of *k* clusters determined by elbow and silhouette methods for each dataset/cohort, method of initialization was set as *k*-means +  + , maximum number of iterations = 300, and 5000 runs of the algorithm with a different centroid seed were set. The final clustering results were determined by the best output of the 5000 consecutive runs by the sum of squared distances of samples to their closest cluster center.

### Development of gene modules and derivation of 32 molecular features

The process for deriving gene modules/signatures for the 134 starting molecular features (Additional file 2: Figure S2) was previously described [13]. Briefly, the gene modules were developed to identify cells and functional processes involved in immune and inflammatory responses specifically. This was done using literature mining, numerous publicly available databases (e.g., the Human Protein Atlas, Interferome, and Gene Cards) and from experience with perturbations in lupus gene expression [13]. Importantly, only genes that unambiguously were expressed by a specific cell type or utilized in a specific cellular function were incorporated into the final modules and a specific gene was only included in one module, with few exceptions. The interferon (IFN) gene module includes genes reflective of both the type I and type II interferon pathways. The low-density granulocyte (LDG), granulocyte, and neutrophil modules are distinct from each other; the granulocyte and neutrophil modules only share one gene, *CD177*. Briefly, the LDG signature was derived from the differential expression of LDGs to healthy control neutrophils and to SLE neutrophils, and consists largely of genes encoding neutrophil granule proteins [11, 13].

Determination of lupus endotypes was based on enrichment of 32 features, each comprised of a gene module/signature (Additional file 2: Figure S2). Feature selection involved an initial step of culling a library of 134 gene modules for tissue-specific and repeated signatures, yielding 72 gene modules that were used as input for gene set variation analysis (GSVA) [23] of samples from datasets 1–3 and 8 (Additional file 1: Table S1). GSVA was run on each dataset separately. Low-intensity genes were filtered and only those with interquartile range (IQR) > 0 across all the samples were retained for analysis. GSVA was also carried out separately for two feature selection cohorts within each dataset: (1) Lupus versus healthy controls, and (2) active (SLE Disease Activity Index (SLEDAI) ≥ 6) lupus versus inactive (SLEDAI < 6) lupus. For each of these two stratifications, GSVA enrichment scores from each dataset were concatenated; that is, a matrix of all 72 features by all samples from each dataset were combined, providing a sufficiently large cohort of patients for feature extraction and stratification.

Various feature selection techniques were employed to remove the noise and select features that contributed the most to the prediction variable. The concatenated GSVA score matrices included Feature Selection Cohort 1, comprised of 1907 SLE samples and 73 non-SLE healthy controls, and Feature Selection Cohort 2, comprised of 1663 active SLE samples and 244 inactive SLE samples from the four aforementioned datasets. See “Binary Classification to Derive 32 Molecular Features.”

Feature refinement was carried out independently in each Feature Selection cohort in Python v. 3.8.2 using scikit-learn (v. 0.24.1) [29]. First, the data were checked for missing values and low variance; neither were detected. Next, features were correlated by Pearson correlation to identify and remove redundant features. Pairs of features with correlation coefficient *r* > 0.9 were identified as highly correlated and ten gene modules were excluded.

Feature importance was used to identify the top features involved in classifying the two feature selection cohorts. Of nine ML algorithms, support vector machine (SVM) and random forest (RF) were most effective in each classification determined by sensitivity, specificity, Cohen kappa score, f1-score, and accuracy. Gini index (RF) and permutation importance (SVM) were employed to calculate the feature importance scores and the top 20 in each feature selection cohort by both algorithms were identified and selected. Redundant features were removed for a final 32 features. The immunoglobulin (IG) chain module was re-added (included in the 32) because we previously demonstrated the importance of specific IG chains in an ML model classifying lupus in patients of African ancestry (AA) from those of European ancestry (EA) [13].

### Binary classification to derive 32 molecular features

Specifically, two independent binary classifications on the feature selection cohorts were carried out in Python v. 3.8.2 using scikit-learn (v. 0.24.1) [29]. Several linear, nonlinear, and ensemble ML algorithms were implemented to distinguish lupus from healthy, non-lupus controls, and active lupus from inactive lupus. Since there were data imbalances in both feature selection cohorts, subsampling without replacement was carried out by creating 20 different folds/subsets by randomly selecting 73 lupus samples to match with the minority class in Feature Selection Cohort 1 and by creating 7 different folds/subsets of 250 random active lupus samples to match with the minority class in Feature Selection Cohort 2. The data from each fold were split into 70% training and 30% validation, and ML classifiers were built on the training data and evaluated on the validation data. Feature importance scores were computed for RF and SVM classifiers. Average performance measures were calculated from all 20 folds of Feature Selection Cohort 1 and 7 folds of Feature Selection Cohort 2. Average gene importance scores of the respective folds were computed. ROC curves and precision recall (PR) curves were plotted using the matplotlib (version 3.3.4) Python library [30].

### ML classification of final endotypes

**Fig. 1:**
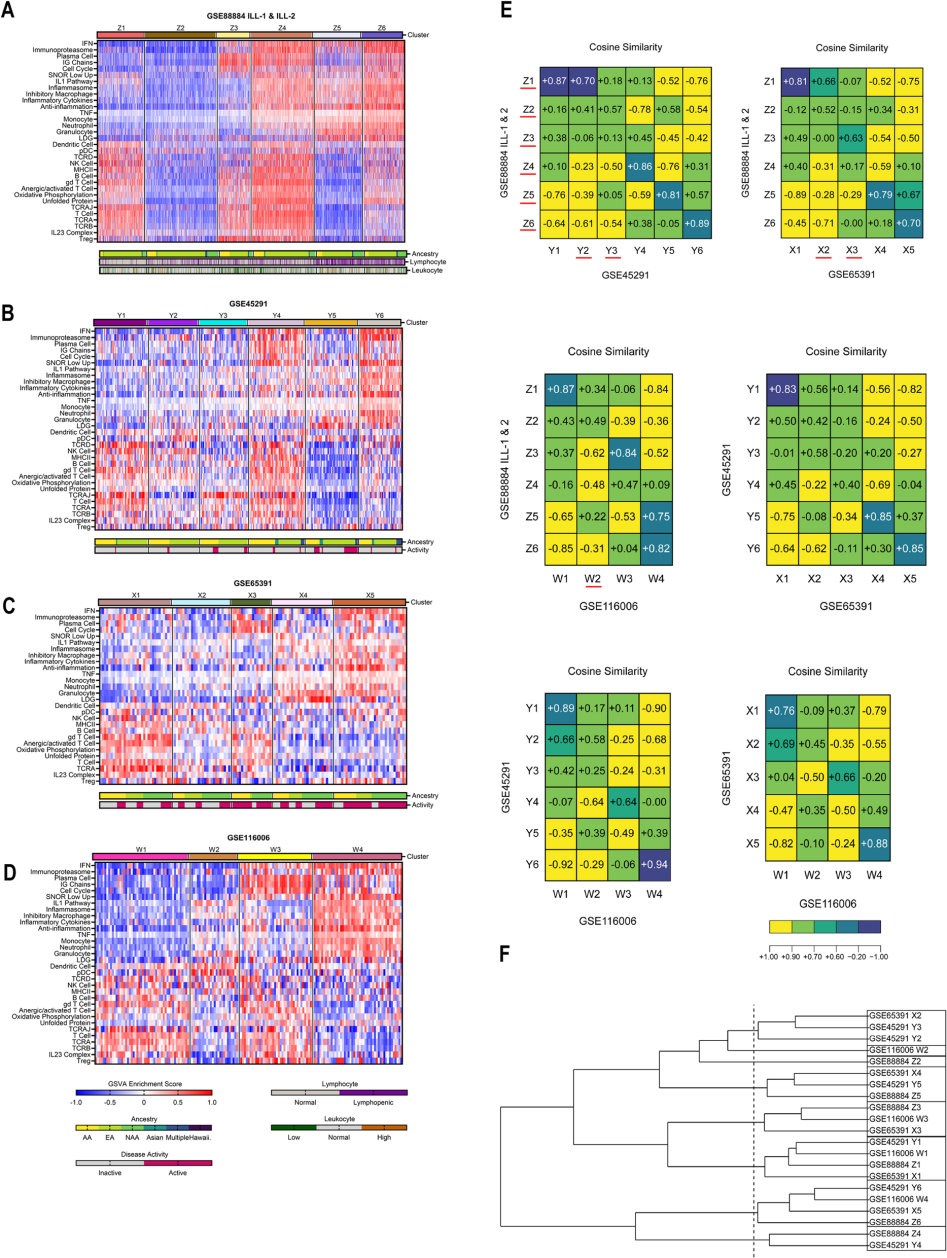
Identification of the optimal number of lupus endotypes using five datasets *K*-means clustering of GSVA scores of the 32 features in **A** 1620 adult female lupus patients from GSE88884 (ILL-1 & ILL-2) yielded six clusters using baseline gene expression, **B** 266 adult lupus patients from GSE45291 yielded six clusters, **C** 137 pediatric lupus patients from GSE65391 yielded five clusters, and **D** 160 adult lupus patients from GSE116006 yielded four clusters using baseline gene expression. Of note, only 28 features were used in GSE65391 because of the microarray chip restrictions. **E** Cosine similarity analysis and **F** hierarchical clustering of the endotypes identified in these five datasets led to a final designation of eight transcriptionally distinct endotypes. Endotypes were considered similar if their cosine similarity was > 0.7. Endotypes underlined in red in **E** indicate the unique endotypes between datasets. Hierarchical clustering using complete agglomeration and a cut height of 1.8 is displayed in **F**. If available, ancestry, disease activity (where active indicates SLEDAI ≥ 6), lymphopenia (< 1 billion lymphocytes/L), and leukopenia (< 3.8 billion leukocytes/L) are annotated with color bars below each heatmap. Heatmaps were generated in GraphPad Prism v. 9.4.0 (673). In **E**, heatmaps were generated in R using the plot.matrix package and edited in Adobe Illustrator. Dendrogram in **F** was generated in R using the ggplot2 package

**Fig. 2:**
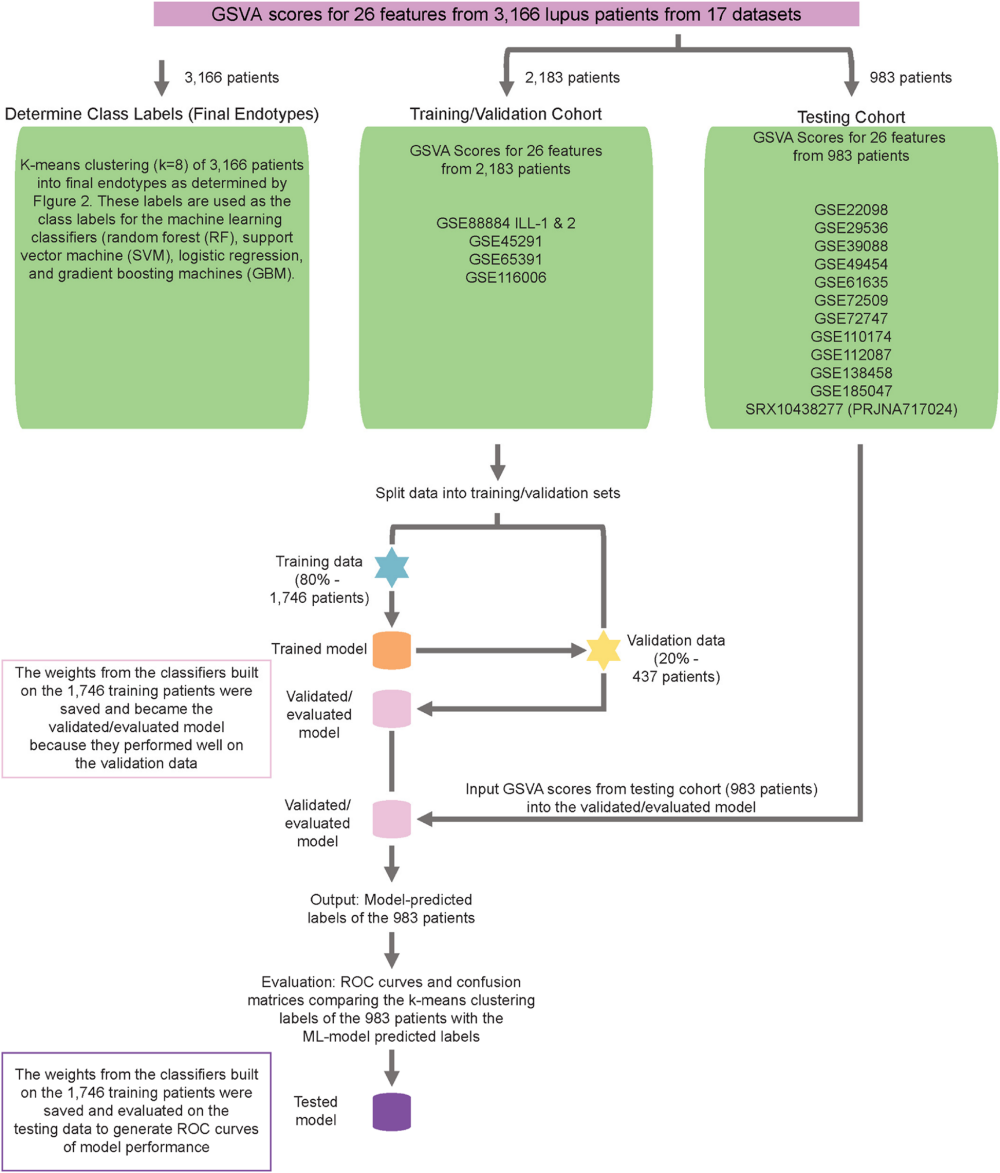
Experimental design of ML models to classify patients into final eight endotypes *K*-means clustering (*k* = 8), division of data into training, validation, and testing cohorts, and machine learning workflow to generate classifiers to predict final eight endotype membership of lupus patients. Flow diagram created in Adobe Illustrator

**Fig. 3:**
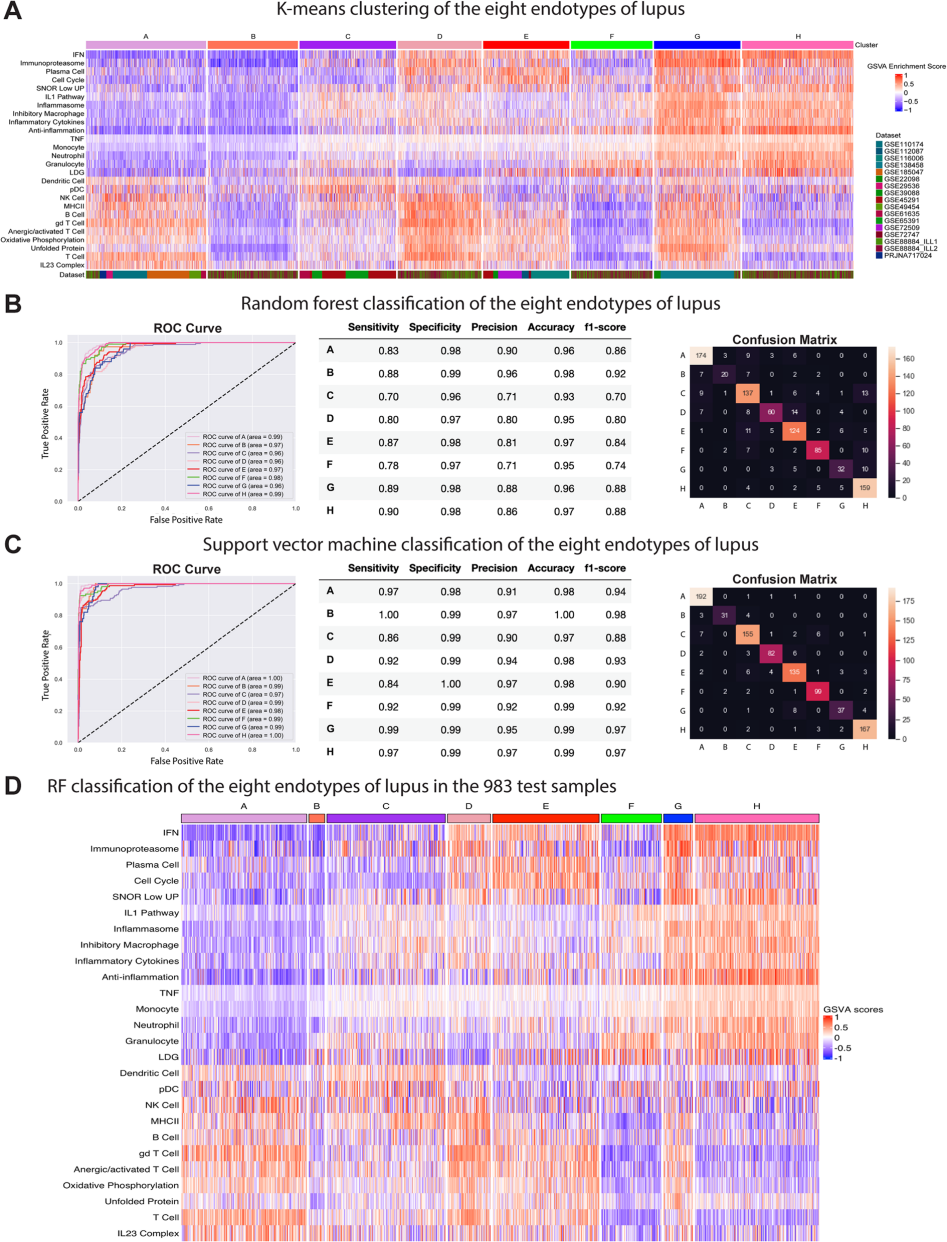
Machine learning algorithms can predict lupus endotype membership with high accuracy **A** Eight final endotypes of lupus were determined by *k*-means clustering of the 3166 patients’ concatenated GSVA enrichment scores of 26/32 features. Multi-class classification by ML categorized 3166 lupus patients from 17 datasets into eight patient endotypes. Area under the ROC curve (AUC), performance metrics, and confusion matrices for each of 4 classifiers on the testing cohort data (983 samples) are summarized: **B** random forest and **C** support vector machine. Each model was trained on 1746 lupus samples, validated with 437 lupus samples, and tested on the remaining 983 samples for a total *n* = 3166 from 17 datasets. **D** RF classification of the 983 samples into the eight endotypes. Heatmaps in **A** and **D** were generated in R with the ComplexHeatmap package. Plots in **B**, **C** were generated in Python using the scikit-learn and matplotlib libraries

In total, 3166 lupus patients from all 17 datasets were endotyped and used to train, validate, and test an ML classification tool. Five datasets (Table 1, Additional file 1: Table S1, Datasets 1–5) were used to determine the optimal number of endotypes (Fig. 1, Additional file 2: Figure S1). GSVA enrichment scores of 26 of the 32 molecular features that were measurable in all 17 datasets were input into the *k*-means clustering pipeline to arrive at eight endotypes—the class label for ML (Figs. 2 and 3A). The initial five datasets were also used to train and validate ML classifiers before testing on 12 independent datasets (Figs. 2 and 3B, C, Additional file 2: Figure S3, Additional file 1: Table S1, Datasets 6–17).

More specifically, with the input GSVA data and labels (Additional file 1: Table S3) from *k*-means clustering of 3166 lupus samples into eight endotypes, the samples were then split into training and validation (Additional file 1: Table S1, Datasets 1–5, *n* = 2183) and test (Additional file 1: Table S1, remaining datasets, *n* = 983) sets. One-vs.-one and one-vs.-rest multi-class classifications with leave-one-out cross-validation were employed to predict sample membership into one of eight lupus endotypes. Training data (*n* = 2183) were further split into 80% training and 20% validation data. Synthetic Minority Oversampling Technique (SMOTE) was applied on the training data to handle class imbalances [31]. RF, SVM, LR, and GB were employed in one-vs.-one and one-vs.-rest multi-class classification and XGB was additionally employed in one-vs.-rest multi-class classification. The ML models were built on training data, optimized, if necessary, using validation data by fine-tuning parameters, and their performances evaluated on the test sets based on sensitivity, specificity, Cohen’s kappa, f1-score, and accuracy. Non-lupus healthy controls were excluded from these analyses. ML was carried out in Python v. 3.8.8 using the scikit-learn (v. 0.24.1) library. ROC curves and PR curves were plotted using the matplotlib (v. 3.3.4) Python library.

Precise contributions by important features for each molecular endotype were identified using SHapley Additive ExPlanations (SHAP) [32]. Feature contribution and SHAP value plots, dependence plots, and waterfall plots were carried out and visualized in Python v. 3.8.8 using the shap module v. 0.39.0.

### Centroid-based ML validation to classify patients into final endotypes

As one validation of the ability of ML to classify lupus patients into one of the eight final endotypes, an alternate ML pipeline was developed with different class labels. The 3166 patients from 17 datasets were similarly split into train/validation and test datasets. *K*-means clustering (*k* = 8) was carried out on the training data (i.e., GSVA scores of 2183 samples comprised from 5 datasets). The cluster centers or centroids of these data were saved as a pickle file using the “pickle” Python module. The labels of the test data were determined by using these centroid definitions. That is, test data labels were assigned by calculating the distance to the training set-defined centroids of the GSVA scores and labeling the data point to the nearest centroid accordingly. These labels were then compared to the clusters A–H generated from the entire 3166 patient cohort by cosine similarity.

### Unsupervised ML validation to classify patients into final endotypes

As a second validation of the ability of ML to classify lupus patients into one of the eight final endotypes, a third ML pipeline was developed with different class labels. The 3166 patients from 17 datasets were similarly split into train/validation and test datasets. *K*-means clustering (*k* = 8) was carried out on the training/validation data (i.e., GSVA scores of 2183 samples comprised from 5 datasets). Five different ML models (LR, SVM, RF, GB, XGB) were trained using input GSVA data *k*-means labels and model weights were generated. The model weights were then used to predict the class of the patients from the 12 independent testing datasets (*n* = 983), for which we did not have true labels. The ML model-generated labels of the 983 test cohort patients were compared to the *k*-means generated labels of the 2183 training/validation cohort by cosine similarity.

### Binary classification to characterize individual endotypes from the least abnormal endotype using SHAP and Gini Index

Seven individual binary ML classifications with RF were performed comparing the seven more transcriptionally abnormal molecular endotypes with the least abnormal endotype (endotype A) in Python v. 3.8.2 using scikit-learn (v. 0.24.1). Similar to the multi-class classification methodology, training data (Additional file 1: Table S1, Datasets 1–5) were divided into training (80%) and validation (20%) sets for which classifiers were optimized if necessary and performance metrics evaluated on unseen test data (Additional file 1: Table S1, remaining 12 datasets). For each classification, the top 15 contributors of each abnormal endotype were determined using SHAP (in Python v. 3.8.8 using the shap module v. 0.39.0). SHAP dependence plots were generated to illustrate the impact of each feature on the final model outcome and its interaction with another feature. SHAP waterfall plots were also generated for individual samples to visualize and deconvolute the mathematical contribution of each feature to the overall SHAP value. SHAP bar plots were additionally generated to summarize the information from the waterfall plots across all samples. SHAP values for each of the classifiers were also visualized using the dot plot function from ggplot2 (v 3.3.3) Bioconductor package in R. Gini index analysis of the classifiers was also performed using the sklearn Python module and the Gini indices were visualized using dot plot function from ggplot2 (v 3.4.0) Bioconductor package in R. Example scripts for GSVA, *k*-means clustering, ML, data normalization, and SHAP analysis can be found at: https://github.com/pbachali16/ML-reveals-endotypes-in-lupus-samples-using-transcriptomic-features.git [33].

### Lupus Cell and Immune Score (LuCIS)

To summarize the data generated by *k*-means clustering of the 32 GSVA enrichment scores, a composite score, LuCIS, was developed using ridge-penalized logistic regression (RPLR). The GSVA enrichment scores of the 32 molecular features calculated for each lupus patient in the bookend clusters of GSE88884 ILLUMINATE-1 (ILL-1) & ILL-2 (i.e., the least abnormal endotype, Z1, and the most abnormal endotype, Z6) were input into a ridge regression algorithm with penalty. The resulting model provided the coefficients for LuCIS. To calculate a LuCIS value for each lupus patient, the GSVA enrichment scores for each module were binarized into zero or one based upon whether the GSVA score was less than zero or greater than zero, respectively. The binarized GSVA scores for each module were multiplied by the LuCIS coefficient in each patient and summed to create a raw LuCIS, which was normalized to a positive value. The ridge regression model was generated using glmnet from the “caret” R package v. 6.0–92 [34].

The relationships between LuCIS, SLEDAI, anti-dsDNA titers and complement levels were determined by Pearson correlations calculated in GraphPad Prism v. 9.4.0 (673).

LuCIS-defined subsets were also used to evaluate the relationship between LuCIS and flare or clinical response. Patients in GSE88884 ILL-2 were categorized into sextiles based on their LuCIS value. Each LuCIS sextile accounted for 1/6 of the total possible LuCIS space (maximum LuCIS = 14.39). Specifically, the LuCIS ranges for each sextile were as follows: Sextile 1: 0–2.4, Sextile 2: > 2.4–4.8, Sextile 3: > 4.8–7.2, Sextile 4: > 7.2–9.6, Sextile 5: > 9.6–12, Sextile 6: > 12–14.4. Significant differences in responses among sextiles were determined by the chi-square test for trend for multiple groups and, otherwise, the chi-square test for independence in GraphPad Prism v. 9.5.0 (730) or using the prop.trend.test function from the stats package [35] in R. Each test was performed independently on each sextile.

### Clinical evaluation and response measurement

Clinical metadata from both GSE88884 ILL-1 & ILL-2 and GSE65391 were used to characterize the transcriptomic-determined endotypes. Additional metadata for GSE88884 ILL-1 & ILL-2 included severe flare, defined by the SELENA-SLEDAI Flare Index [36]. Significant associations of clinical traits and endotypes were determined by the Kruskal–Wallis and Dunn’s multiple comparisons test in GraphPad Prism v. 9.1.0 (221) or the chi-square test for independence.

SLE responder index (SRI)-4 and SRI-5 from the ILL-2 trial (GSE88884) were used to assess clinical responders to tabalumab in the molecular endotypes [37]. Significant differences in responses among treatment groups were determined by the chi-square test for trend for multiple groups and, otherwise, the chi-square test for independence in GraphPad Prism v. 9.5.0 (730) or using the prop.trend.test function from the stats package [35] in R.

### Gaussian mixture variational autoencoder (GMVAE)

GMVAEs are powerful generative models which feature a pair of connected networks: an encoder, which converts high-dimensional input data into a smaller and denser representation by introducing latent variables, and a decoder, which outputs the probability distribution of the data [38]. Twenty clinical variables including ancestry (binary variables for having European, African, or Hispanic ancestry), SLEDAI components (binary variables for having arthritis, proteinuria, low complement, leukopenia, mucosal ulcers, rash, pleurisy, and vasculitis), five autoantibody titers (anti-dsDNA, anti-RNP, anti-Sm, anti-SSA, anti-SSB), and medication use (binary variables for taking antimalarials, corticosteroids, immunosuppressants, and non-steroidal anti-inflammatory drugs) from the lupus patients enrolled in ILLUMINATE-2 (GSE88884) were used as input for deep, unsupervised clustering by the GMVAE algorithm. The categorical clinical variables were binarized. GMVAE with back-propagation optimization identified six clusters.

### GSVA Enrichment Score Imputation

Because of inter-platform differences (i.e., microarray chips are restricted by their specific libraries), some GSVA gene sets were not represented across all datasets. To overcome this limitation, GSVA enrichment scores were imputed for each patient based upon their known relationship to other represented gene sets.

Some GSVA scores were imputed on a dataset-by-dataset basis. In these cases, the gene set with the highest Pearson correlation was used to first estimate a coefficient describing the relationship between the gene set of interest (i.e., the one that requires score imputation) and the correlated gene set. For each of these gene sets of interest (i.e., IG chains, TCRA, TCRAJ, TCRB, TCRD, and Treg), the most correlated gene set was found by computing Pearson correlations between GSVA enrichment scores of all 32 features in GSE88884 ILL-1 and ILL-2. These datasets were used as a reference (their GSVA scores were computed separately, then concatenated) due to the large number of samples and because all 32 gene sets were represented on the corresponding microarray chip. Next, a value of one was added to the GSVA scores of the “highest correlated modules” and the “modules of interest” so that there would not be any negative GSVA scores. Then, for each sample, the transformed score of the “module of interest” was divided by the transformed score of the “highest correlated module.” The mean of these calculations across the patients is the coefficient describing the relationship between the two modules.

In the dataset requiring imputation, a value of one was added to the GSVA scores of the “highest correlated module,” multiplied by the appropriate coefficient, and then a value of one was subtracted to arrive at the GSVA enrichment score of the gene set of interest.

## Results

### Identification of molecular endotypes in five representative lupus datasets

Six molecular subsets (endotypes) within 1620 active, female lupus patients in the combined datasets from the ILL clinical trials (GSE88884 ILL-1 & ILL-2) were identified by *k*-means clustering of GSVA enrichment scores of 32 immune cell/inflammatory pathway gene modules (Fig. 1A, Additional file 2: Figure S1). Endotypes labeled as Z1-Z6 were distinguished by patterns of enriched (red) and unenriched (blue) gene modules. To assign SLE endotypes as normally or abnormally enriched, lupus patient and control samples were re-clustered together (Additional file 2: Figure S4). Most non-lupus control samples clustered with the least abnormal SLE samples (11/17 in Z1’), indicating that these SLE patients had minimal to no gene expression differences from controls; analysis by cosine similarity indicated their module identity. Features were considered as abnormally enriched or expressed based on their GSVA enrichment score as compared to controls. For example, in non-lupus controls, modules such as IFN, plasma cell, IG chains, myeloid cell, and tumor necrosis factor (TNF) tended to have GSVA scores less than zero, whereas modules such as B cell, T cell, and T cell chains (TCRA, TCRAJ, TCRB, TCRD) tended to have GSVA scores greater than zero. Within the lupus patient samples, the endotype designated Z1 manifested the least number of abnormally enriched gene modules (features), whereas the endotype designated Z6 had the greatest number of abnormally enriched features, with other endotypes arrayed between.

Next, we extended this endotyping approach to include other unrelated datasets, two with both active and inactive patients, and a large RNA-seq dataset, to determine whether the same or additional endotypes could be detected (Additional file 1: Table S1, Datasets 3–5). Within these three other datasets, six endotypes were identified in a cohort of 266 adult lupus patients, five endotypes were identified in a cohort of 137 pediatric lupus patients, and four endotypes were identified in 160 other adult lupus patients (Fig. 1B–D, Additional file 2: Figure S1). As with GSE88884, non-lupus controls tended to cluster in the “least perturbed” endotype with similar enrichment patterns as Z1 (Additional file 2: Figure S5).

Cosine similarity analysis indicated that several of these SLE endotypes were reproducible among all datasets, whereas others were found in only some datasets (Fig. 1E). Of note, when compared to a large cohort of adult lupus patients, three shared subsets and two transcriptionally distinct subsets emerged in the pediatric patients using a cosine similarity cutoff of 0.7. Combining all unique endotypes across datasets indicated that a total of 11 endotypes can be identified by cosine similarity. However, hierarchical clustering of the mean GSVA scores of the individual endotypes suggested that after eight subsets, the next three subsets emerged within a very small statistical space (Fig. 1F), namely, that with a slightly lower cut height, eight endotypes were identified, which indicated that these three additional subsets were similar to other subsets even though they missed being captured by cosine similarity. Furthermore, the additional subsets contained very few members compared to the eight endotypes. In summary, these analyses conservatively identified eight as the optimal number of SLE endotypes. *K*-means clustering of the eight identified endotypes from the five datasets demonstrated distinct molecular patterns (Additional file 2: Figure S6A). Principal component analysis (PCA) and t-Distributed Stochastic Neighbor Embedding (t-SNE) demonstrated good separation of the subsets (Additional file 2: Figure S6B-C).

### ML to classify SLE samples into endotypes

To identify the eight endotypes of lupus among a complete cohort of 3166 SLE patients, we concatenated the GSVA enrichment scores of 26/32 measurable features across 17 datasets and employing the *k*-means (*k* = 8) pipeline, subsetted all samples into eight endotypes labeled A-H (Figs. 2 and 3A). These results became the class labels for subsequent ML. It was essential that all subsets have a “true label” in order to validate subsequent ML algorithms, so this step was employed to assign subset designation of all patients within the universe of 3166 SLE patients examined. Each endotype was characterized by unique patterns of dysregulation of the 26 informative gene modules (Additional file 2: Figure S7).

Next, we employed GSVA scores from the five training/internal validation datasets (*n* = 2183, Additional file 1: Table S1, Datasets 1–5) to generate an ML model to classify patients into the final eight endotypes. Testing and external validation were carried out on gene expression profiles of 983 patient samples from 12 additional, independent datasets not used to generate the ML model (Additional file 1: Table S1, remaining datasets). The training data from the 2183 samples were further partitioned into training (80%) and validation (20%) sets and one-vs.-one multi-class classification by multiple algorithms was carried out to predict endotype memberships and carry out internal validation. Receiver operating characteristic (ROC) curves for RF, SVM, logistic regression (LR), and gradient boosting (GB) models were generated on the 983 patients from 12 test datasets not used to generate the model (external validation) and demonstrated high predictive capabilities (Fig. 3B, C, Additional file 2: Figure S3). LR had the highest precision overall with sensitivity ranging from 89 to 100% and specificity ranging from 99 to 100% for the eight endotypes. Altogether, all the classifiers worked well, and even though SVM and LR appeared to be somewhat more effective, we selected RF because it was effective in both multi-class and binary analyses and also afforded the opportunity to access many feature importance techniques. Classification of the 983 patients from the test set into the eight endotypes using the RF classifier is shown (Fig. 3D).

Because of concern that assignment of “true labels” might have biased the outcome of the external validation, we carried out an additional ML approach, the centroid-based ML validation, in which “true labels” were not assigned by *k*-means clustering of the GSVA scores of the entire 3166 patients. Rather, labels for the test set (983 patients, 12 datasets) were generated by centroid similarity of the GSVA scores to the training set. With this external validation ML design, we observed classification performance with high accuracy (Additional file 2: Figure S8-10). A third ML approach, the unsupervised ML validation, was also carried out, in which class labels were generated using only the initial five training/validation datasets. Five different ML models (LR, SVM, RF, GB, extreme GB, or XGB) were trained and model weights were generated. The model weights were then used to predict the class of the patients from the 12 independent testing datasets, for which we did not have true labels (Additional file 2: Figure S11A). When the output of the ML models on the 12 datasets were compared to the outputs from the five training/validation datasets using cosine similarity, it can be seen that there was a high level of sameness of the derived endotypes (cosine similarity > 0.9 for each comparison) for each of the five ML algorithms employed (Additional file 2: Figure S11B-F). This third orthogonal approach clearly shows that the ML model generates the same endotypes from unrelated datasets in an unbiased manner and validates the performance of the models.

To determine whether all 26 features were required to achieve similar performance, we analyzed the model performance when features were randomly removed. We found that all 26 features were needed to identify the eight endotypes by *k*-means optimally (Additional file 2: Figure S12). In addition, the RF classifier and other ML models were largely built using female patients. To ensure the model was applicable to both sexes, we applied the RF classifier from the first ML approach (Additional file 2: Figure S8-10) to a large cohort of male patients (Additional file 2: Figure S13). Indeed, we observed all endotypes in the male cohort, demonstrating these endotypes are not restricted to female patients.

### Development of composite metric LuCIS

**Fig. 4:**
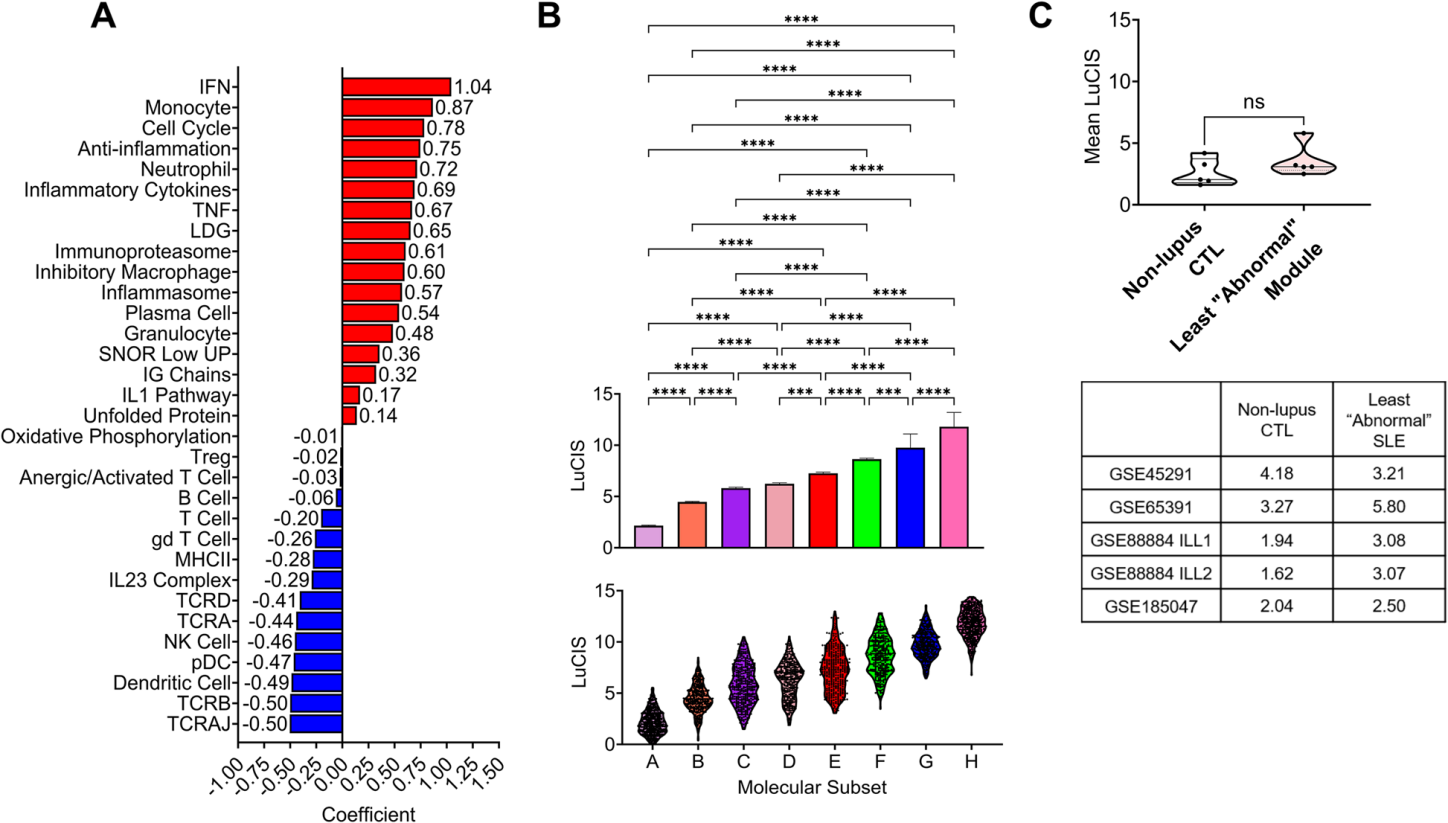
LuCIS summarizes the severity of molecular abnormalities in individual lupus patients Logistic regression with ridge penalization was employed to classify endotype A (Z1), the “least abnormal,” and endotype G/H (Z6), the “most abnormal,” from GSE88884 ILL-1 & ILL-2 which produced coefficients (**A**) that can be used to calculate LuCIS. **B** The mean + SEM (top) and distribution (bottom) of LuCIS calculated for the eight endotypes in all 17 datasets using the 26 features shown in **A** and the imputed values for the six features not measured on all platforms (IG chains, TCRA, TCRAJ, TCRB, TCRD, Treg) as described in the Supplemental Methods. Statistical differences between mean LuCIS of the endotypes were evaluated with the Kruskal–Wallis test and Dunn’s multiple comparisons. **C** Comparison of mean LuCIS between non-lupus healthy controls and the least “abnormal” lupus endotype in five independent datasets for which adequate controls were available. Missing values for GSVA modules not measured (IG chains, TCRAJ, TCRB, TCRD) in GSE65391 were imputed as described in the Methods. All plots were generated, and statistics were computed, in GraphPad Prism v. 9.4.0 (673)

With the identification of eight endotypes representing the apparent universe of lupus patients and high predictive capability of ML algorithms, we sought to reduce the information from gene expression profiles into a clinical metric, designated LuCIS, to display the range of molecular abnormalities numerically. An RPLR model was employed to calculate a LuCIS value for each patient based on his or her binarized GSVA enrichment score (Fig. 4A). These scores were then compared to the placement of each patient in our eight lupus endotypes and showed increasing LuCIS values for each endotype (Fig. 4B).

To contextualize LuCIS values, we calculated the scores of healthy, non-lupus controls in five datasets for which adequate control samples were available (Fig. 4C). Notably, the mean LuCIS values of the least abnormal lupus endotypes were not significantly different from those of the non-lupus controls, indicating that LuCIS identified the least abnormal endotypes’ resemblance to a normal transcriptional profile.

### Use of SHAP to determine the most important features of endotypes

We determined the most important GSVA enrichment scores contributing to the endotype groupings using additional ML classification and SHAP. To accomplish this, we used ML to classify samples using one-vs.-rest multi-class classification (Additional file 2: Figure S14) and then employed SHAP to compute the contribution of each feature to each endotype. A summary of mean absolute SHAP values across patients in the eight endotypes from the extreme GB classifier revealed the top 20 features contributing to the model, with gamma/delta (gd) T cells, major histocompatibility complex II (MHCII), and IFN being the overall most impactful (Additional file 2: Figure S15). Anti-inflammation, granulocyte, and neutrophil features most distinguished endotype H, the most perturbed endotype (Fig. 3A), whereas the lack of enrichment of monocytes, anti-inflammation, and IFN most impacted the least perturbed endotype, endotype A.

To elucidate the specific features determinant of the eight endotypes, we additionally employed seven binary classifications, each comparing one of the seven more transcriptionally perturbed endotypes (B–H) to the least abnormal endotype (A). These classifiers (Additional file 2: Figure S16-22) demonstrated excellent performance with a mean positive predictive value of 0.97 for the RF classifier, but all classifiers performed well (Additional file 1: Table S4).

**Fig. 5:**
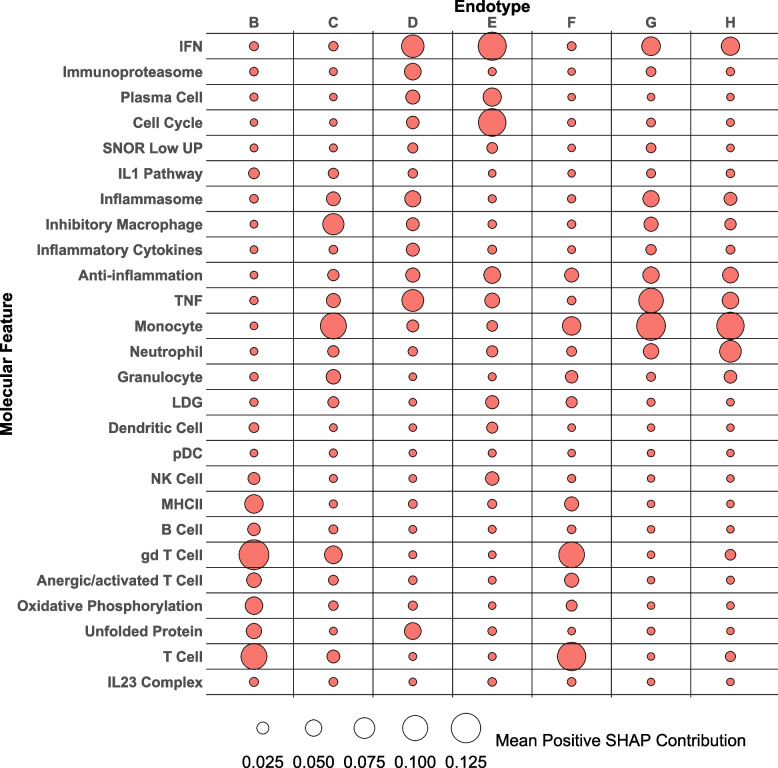
SHAP analysis reveals features most distinctive of transcriptional perturbations in the seven abnormal lupus endotypes SHAP analysis of the seven binary RF classifiers distinguishing the seven out of eight most transcriptionally abnormal lupus endotypes (B–H) from the eighth least abnormal endotype (A) reveals the features most contributory to the ML model’s classification capacity. Mean SHAP values were calculated using only the samples in each more severe endotype (B–H) that were positive. The size of the data points enumerates the positive mean SHAP value of each feature listed on the *y*-axis. Bubble plot rendered in R using the ggplot2 package

SHAP analysis of the RF classifiers was then employed to delineate the features that characterized individual endotypes from those of the most normal lupus endotype (endotype A) (Fig. 5). Data are shown as the features that distinguish endotypes. In addition, the impact of each feature at the individual patient levels is shown as SHAP waterfall plots (Additional file 2: Figures S16-22). Different patterns of features distinguished most of the endotypes (Fig. 5). For example, the endotype with the most immunologic abnormalities (H) demonstrated high contributions from the monocyte, neutrophil, TNF, and IFN signatures (Fig. 5, Additional file 2: Figure S22), whereas T cell signatures were most contributory to endotype B (Fig. 5, Additional file 2: Figure S16). SHAP dependence plots detailed the various effects of the individual features on predictions made by the model with, for example, granulocytes having an impact throughout the range of granulocyte GSVA scores, gd T cells at the transition of GSVA scores from positive to negative, and LDG at the extremes of GSVA scores for distinguishment of endotype B (Additional file 2: Figure S16E). Using the Gini Index, another feature importance metric employed with the RF classifier, we were able to confirm these analyses (Additional file 2: Figure S23).

### Clinical data do not identify molecular endotypes

To test whether clinical characteristics alone could identify the endotypes, we employed our clustering pipeline on ILL-2 lupus patient clinical metadata. With *k*-means, six subsets based on clinical features alone were identified (Additional file 2: Figure S24A). Another six subsets were also identified by separate employment of a variational autoencoder to determine whether a deep learning algorithm would alternatively be able to identify the endotypes (Additional file 2: Figure S24B). The clinical *k*-means subsets were largely dictated by ancestry, whereas the clinical autoencoder subsets were ancestrally heterogeneous. Notably, by Adjusted Rand Index, the clinically determined subsets were significantly different from the molecular endotypes (Additional file 2: Figure S24C-D) and from each other (Additional file 2: Figure S24E). To corroborate this finding, we employed ML classifiers using the same clinical data as features to determine whether they could predict molecular endotype memberships (Additional file 2: Figure S25). Performance was suboptimal, with a mean RF classifier precision of 32%, further indicating that clinical characteristics are insufficient features to identify the molecular endotypes.

### Clinical characterization of SLE endotypes

**Fig. 6:**
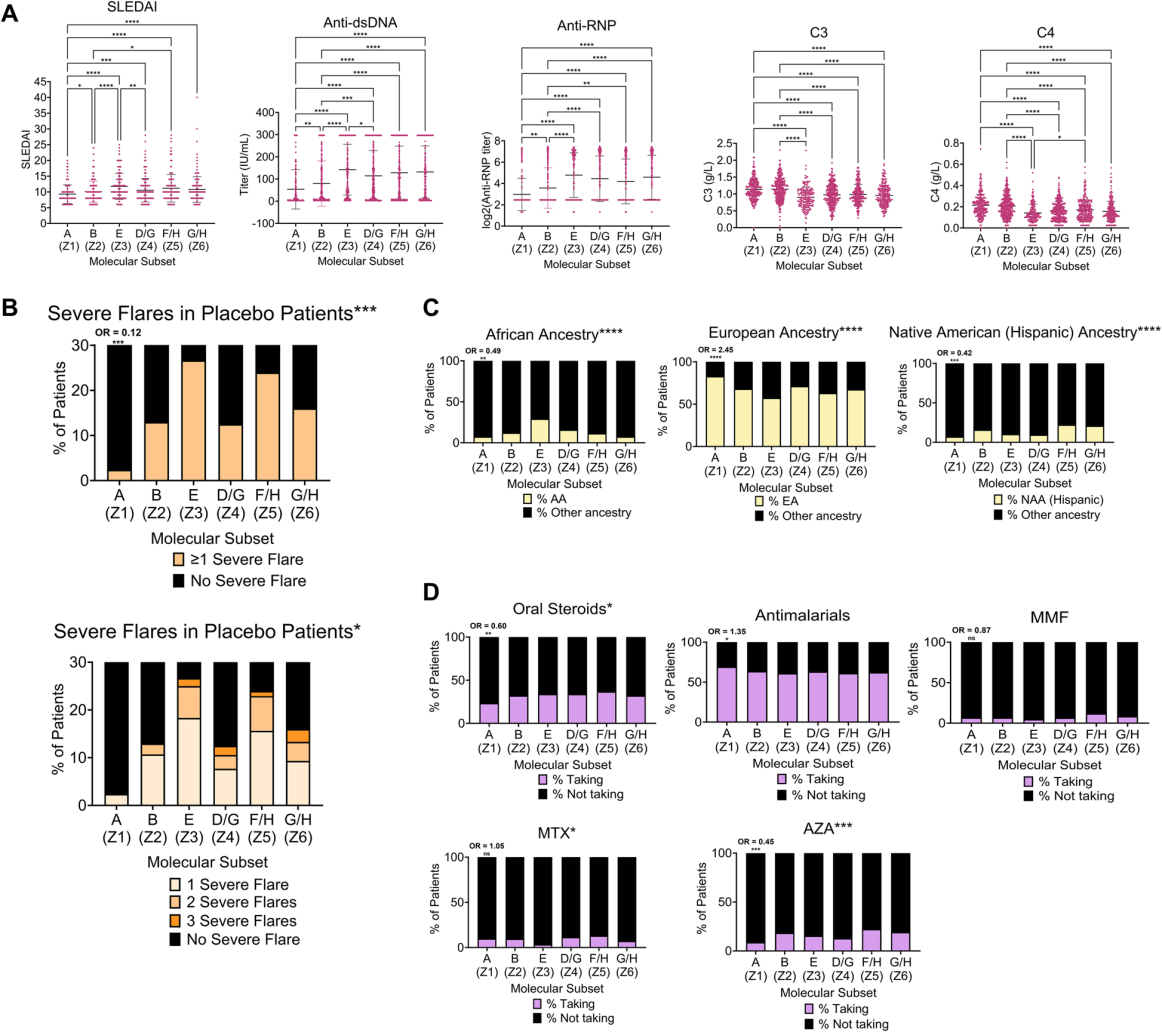
Clinical characterization of SLE endotypes Clinical metadata were summarized for each cluster from GSE88884 (ILL-1 & ILL-2) using baseline values. Metadata was categorized by **A** quantitative immunologic/inflammatory and systemic disease indicators, **B** incidence of subsequent flares over 52 weeks in placebo patients receiving SoC medication (*n* = 550), **C** patient ancestry, and **D** medication use. Labels on *x*-axes indicate the shorthand name for the endotypes. Clusters were relabeled as one of the eight endotypes using cosine similarity. Scatterplots in **A** display the mean ± SD for each endotype; statistical differences were found with Dunn’s multiple comparisons test. Significant associations between categorical variables and endotypes in **B–D** (denoted with asterisks in titles) were identified using chi-square test of independence. In **B–D**, odds ratios of endotype A (Z1) having a positive value for the clinical trait of interest as compared to the other cohorts combined are displayed above the respective bar with significance indicated by asterisks. Missing data (n.d.) were excluded from analyses. All plots were generated, and statistics were computed, in GraphPad Prism v. 9.4.0 (673). MMF = mycophenolate mofetil. MTX = methotrexate. AZA = azathioprine. n.d. = no data. *p < 0.05; **p < 0.01; ***p < 0.001; ****p < 0.0001

Next, we sought to determine whether endotype membership was associated with various clinical features of SLE. For clarity, subsets/endotypes identified in Fig. 1A were re-assigned to a letter classification (A-H) based on cosine similarity to the final eight endotypes (Fig. 6, Additional file 2: Figure S26). Despite all patients having SLEDAI ≥ 6 in GSE88884 ILL-1 & ILL-2, analysis of the associated metadata revealed significant differences among endotypes with respect to SLEDAI, autoantibody titers, lymphopenia, and serum complement levels. Subset A (Z1, the least abnormal) had the lowest SLEDAI, lowest autoantibody titers, and highest complement levels, whereas endotypes E (Z3), D/G (Z4), F/H (Z5), and G/H (Z6) manifested more abnormal clinical characteristics (Fig. 6A, Additional file 2: Figure S27). By these systemic measures, endotype A (Z1) exhibited the lowest disease activity. On the other hand, E (Z3) exhibited the most disease activity, followed closely by F/G (Z6). Endotypes associated with more disease activity were characterized by various combinations of enrichment of features for plasma cells, myeloid cells, neutrophils, inflammatory cytokines, and lymphopenia (Fig. 1A).

Of note, the patients in A (Z1) receiving standard of care (SoC) medication had a lower frequency of severe flares over the subsequent 52 weeks as compared to the patients in other subsets (OR = 0.116, *p* = 0.00041, Fig. 6B, Additional file 1: Table S5). Significant relationships between endotype membership and ancestry (African, European, or Hispanic) (Fig. 6C) and medication use (oral steroids, azathioprine, or methotrexate) were also identified (Fig. 6D). Patients of each ancestry were noted in each subset, although AA lupus patients were most enriched in E (Z3) and least enriched in A (Z1) (Fig. 6C). More subtle but significant differences were found among endotypes for vasculitis, alopecia, leukopenia, arthritis, mucosal ulcers, accompanying organ systems, and the overall number of SLEDAI domains involved (Additional file 2: Figures S28-29).

Because extensive clinical metadata accompanied the pediatric lupus patients (GSE65391), we examined the relationships between endotype and clinical features and found significant differences among endotypes with regard to SLEDAI, complement levels, and ESR, with endotype E (X3) exhibiting the most disease activity (Additional file 2: Figure S30). Although mean anti-dsDNA levels varied among endotypes and were highest in E (X3), intra-group variation was too high to detect significant intergroup differences. Pediatric endotypes also differed by occurrence of lymphopenia (*p* < 0.05), oral steroid use (*p* < 0.01), and HCQ use (*p* < 0.01), but, interestingly, not ancestry (*p* > 0.05, Additional file 2: Figure S30C-E). However, the endotype with the highest SLEDAI, E (X3), contained the highest proportions of AA and Native American Ancestry (NAA/Hispanic) patients and patients with proliferative nephritis (Additional file 2: Figure S30F).

### Relationship between endotypes and ancestry and SoC therapies

Because we previously found that ancestry and SoC therapies, such as mycophenolate, can significantly influence gene expression [13], and there were variations in subset membership based on ancestry and medication use (Fig. 6C-D), we examined these relationships in greater detail. When only EA patients from GSE88884 were clustered (*n* = 1118), the same six endotypes as observed in the full GSE88884 ILL-1 & ILL-2 cohort were represented (Additional file 2: Figure S31). Similar results were seen in the AA population (*n* = 216, Additional file 2: Figure S32), although the percentage of patients in the least abnormal subset was diminished (R1, chi-square, *p* = 0.03) and a plasma cell-enriched subset was more prominent (R3, chi-square, *p* = 0.02). When only NAA/Hispanic patients were clustered (*n* = 232), five out of six endotypes from the full cohort were identified (Additional file 2: Figure S33). Thus, endotype distribution varied based on ancestry, even though most endotypes were observed in each ancestry group.

We repeated these analyses among patients stratified by immunosuppressive agents at baseline and compared distributions of patients to the endotypes in the full prototypic cohort (all active female) to identify which endotypes were maintained (cosine similarity > 0.7, Additional file 2: Figure S34). Treatment with mycophenolate or methotrexate in combination with steroids appeared to deplete the most perturbed endotype, G/H (Z6), whereas this endotype was expanded in groups treated without SoC SLE therapies, without immunosuppressive agents, and without steroids alone. Treatment with steroids with methotrexate also appeared to deplete endotype E (Z3), which exhibited the highest disease activity. The distribution of patients in the least perturbed endotype, A (Z1), also increased with treatment by steroids and immunosuppressive agents. Altogether, these analyses illustrate that some endotypes cannot be found/are reduced (by cosine similarity) in patients treated with immunosuppressive drugs, and implies that therapy can suppress the appearance of specific endotypes. In addition, therapy may contribute to the proportion of patients in each endotype.

### Utility of molecular endotyping in determining patients with likelihood of therapeutic response

**Fig. 7:**
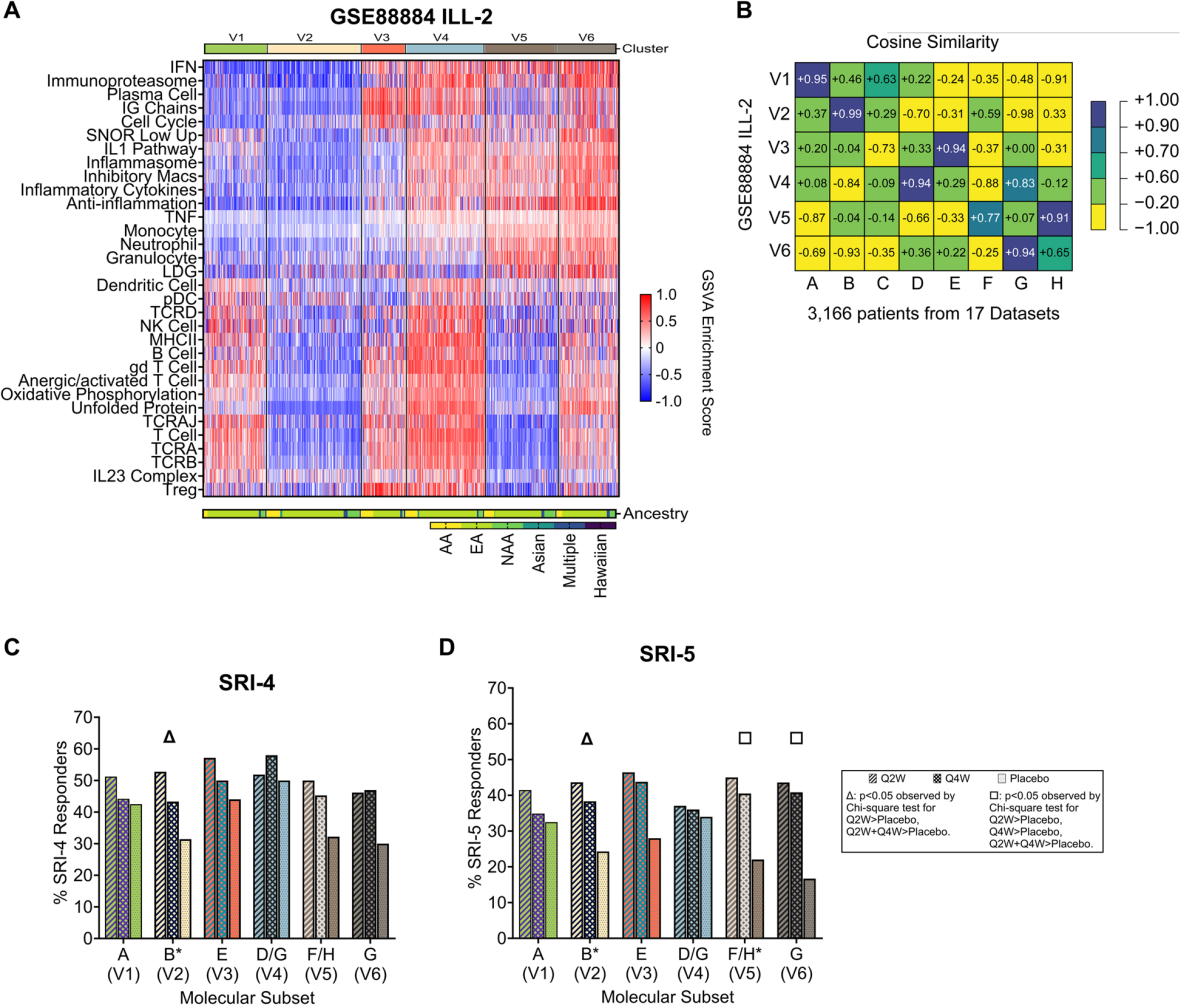
Endotyping to stratify patients who are more likely to respond to treatment Gene expression profiles (**A**) of *k*-means clustering (*k* = 6) of 807 active female lupus patients from GSE88884 ILLUMINATE-2. **B** Cosine similarity comparing the eight global SLE endotypes to the molecular endotypes in ILL-2. Clinical responses by **C** SRI-4 and **D** SRI-5 per endotype determined by gene expression data and 32 features. Responses among the treatment groups in **C** and **D** were ascertained by the chi-square test for trend (all three doses) or chi-square test for independence. Chi-square tests were performed for each endotype individually and summarized on the same plot. Significant results of the chi-square test for trend are denoted in the *x*-axis label of the endotype. Clusters were relabeled as one of the eight global endotypes using cosine similarity. Q2W and Q4W indicate frequency of drug administration was every 2 weeks and 4 weeks, respectively. Heatmap in **A** was generated with GraphPad Prism v. 9.4.0 (673). Cosine similarity plot in **B** was generated with the plot.matrix R package and edited in Adobe Illustrator. Histograms in **C**, **D** were generated with GraphPad Prism v. 9.4.0 (673). *p < 0.05

To explore the clinical utility of molecular endotyping in greater detail, we applied the *k*-means clustering pipeline to the patients belonging to the successful ILL trial, GSE88884 ILL-2 [39], and identified six endotypes similar to those found in the combined trial datasets (Fig. 7A, Additional file 2: Figure S35). Endotypes were re-assigned to A-H based on cosine similarity (Fig. 7B). We examined clinical response to the investigational product, tabalumab, by two metrics: SRI-5, used in the trial, and the more standard SRI-4 [39], among these endotypes (Fig. 7C, D). We identified three responsive groups by SRI-5 (B [V2], F/H [V5], and G [V6]) and one responsive group by SRI-4 (B [V2]), each with a response effect size > 20%. Of note, the endotype with the least immunologic activity (A [V1]) was not responsive by either metric. Notably, although A (V1) manifested a high placebo SRI-5 response rate, the placebo response rates between A (V1) and D/G (V4) were not significantly different (*p* > 0.05). Clinically, the responsive endotypes were characterized by lymphopenia at baseline (chi-square, *p* < 0.0001, OR = 3.2) and were comprised of more patients taking azathioprine (chi-square, *p* < 0.01, OR = 2.0), more NAA/Hispanic patients (chi-square, *p* < 0.0001, OR = 2.6), and fewer EA patients (chi-square, *p* < 0.05, OR = 0.72) (Additional file 2: Figure S36, Additional file 1: Table S6). The responsive endotypes also trended toward having an increased likelihood to experience a severe flare over the subsequent 52 weeks on SoC therapy (chi-square, *p* = 0.06, OR = 2.0).

### Utility of LuCIS in determining likelihood of flare and therapeutic response

**Fig. 8:**
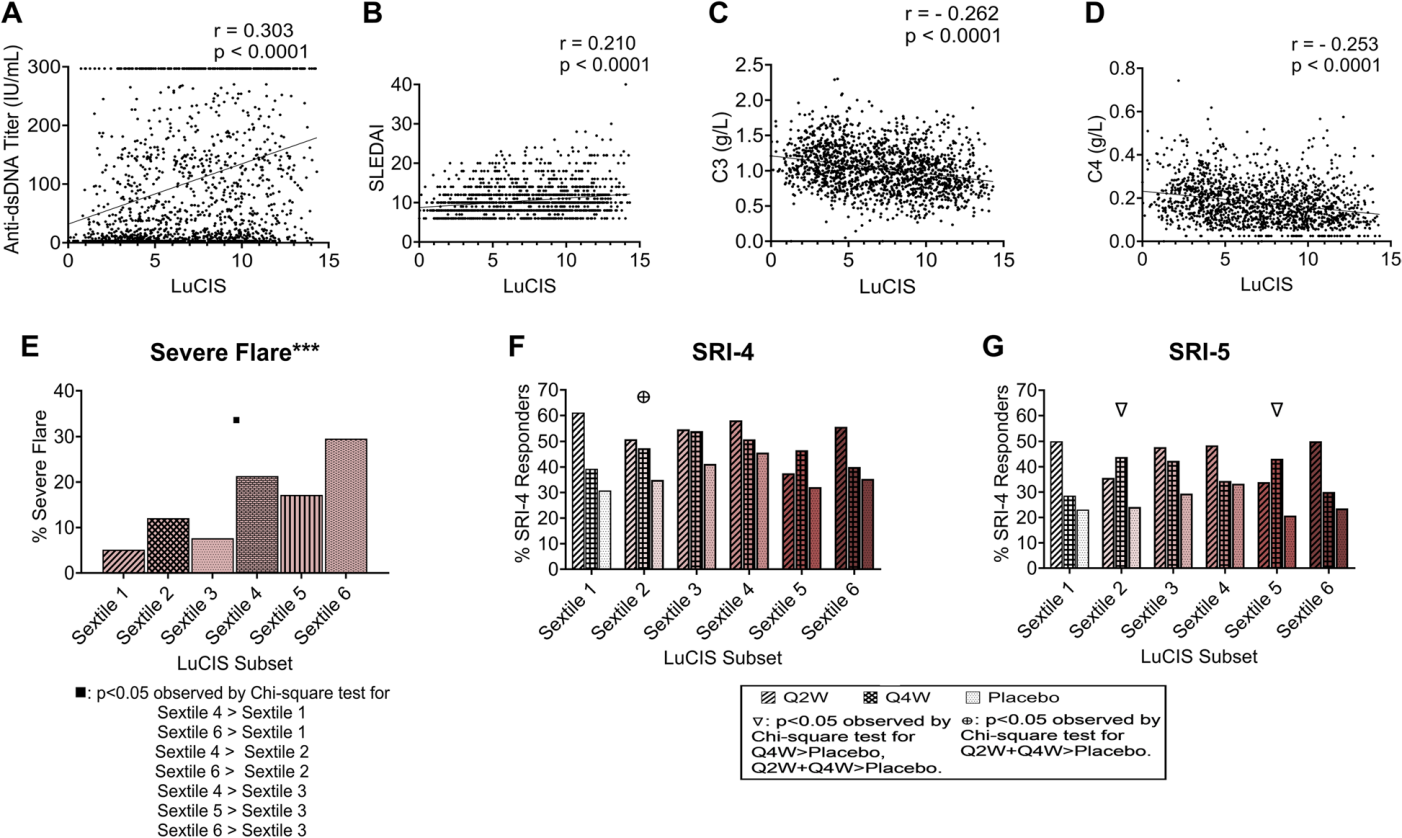
The relationship between LuCIS value and clinical variables, flares or clinical response Pearson correlations were computed between LuCIS values and **A** anti-dsDNA titers, **B** SLEDAI, **C** serum C3, and **D** serum C4 using LuCIS values of all the patients from GSE88884 ILL-1 & ILL-2 (*n* = 1620). LuCIS values were divided into sextiles for GSE88884 ILL-1 & ILL-2 and **E** incidence of subsequent flares over 52 weeks in placebo patients receiving SoC medication (*n* = 550) was examined. LuCIS values were also divided into sextiles for GSE88884 ILL-2, and **F** SRI-4 response and **G** SRI-5 response were examined. Significant associations between incidence of flare among the sextiles (denoted with asterisks in the title) were identified using the chi-square test for trend. Differences in incidence of flare between sextiles were determined by chi-square test of independence and denoted below the figure. Similarly, responses among the treatment groups in each LuCIS sextile independently were determined by the chi-square test for trend (all three doses) or chi-square test for independence. Significant results of the chi-square test for trend are denoted in the plot title. All plots were generated, and statistics were computed, in GraphPad Prism v. 9.5.0 (730). *p < 0.05; **p < 0.01; ***p < 0.001

Finally, we also determined whether there was a relationship between LuCIS values and clinical features. First, we examined the correlation between LuCIS and anti-dsDNA titer, SLEDAI, serum C3, and serum C4 (Fig. 8A–D) in GSE88884 for which full clinical data were available. Positive correlations were identified (*p* < 0.0001) between LuCIS and anti-dsDNA titer or SLEDAI, whereas negative correlations were identified (*p* < 0.0001) between LuCIS and C3 or C4; however, coefficients were modest, suggesting that LuCIS is related to these metrics but may provide additional information not captured by SLEDAI or serology that is reflective of immunologic activity. Next, we used groupings defined by LuCIS values to predict likelihood of flare or response to active treatment in the ILL-2 trial. Patients were assigned to sextiles based on their LuCIS value, to mirror the number of groups identified by the clustering pipeline. LuCIS-defined subsets were associated with a likelihood of severe flare (Fig. 8E) and response to the investigational product in post hoc analysis of the ILL-2 trial (Fig. 8F, G).

## Discussion

The specific disease manifestations in individual lupus patients vary greatly and are difficult to predict. In addition, efforts to stratify lupus patients into clinically informative and actionable groups have been largely unsuccessful and often reduce to differences in serologic features, systemic features, patient demographics, and the presence (or absence) of an interferon signature [9, 40–42]. Herein, we describe a novel characterization of lupus patients based on identifiable endotypes using inflammatory and immunologic transcriptomic features, *k*-means clustering, and ML.

Using 17 datasets that contained diverse patients and data from different gene expression platforms, we were able to identify eight total endotypes representative of the apparent universe of molecularly defined lupus. With these data, we were able to overcome dataset-specific heterogeneity and develop two independent, informative tools: a multi-class ML classifier that predicts endotype membership of individual lupus samples, and a transcriptomic-based composite score, LuCIS, that estimates the level and severity of lupus-related immunologic activity. The ML classifiers showed high predictive capabilities after validation in unrelated datasets, demonstrating the robustness of the endotypes. Further interrogation of features contributing to the ML models demonstrated specific gene signatures involved in the classification of patients into endotypes that could be further probed for druggable targets. The LuCIS calculations demonstrated an overall worsening of lupus-related immunologic aberrancies as endotype membership progressed from A to H and that, in combination with endotyping, LuCIS values could serve as a new clinical metric to estimate lupus activity not captured by current approaches. It is important to note that LuCIS merges together various immunologically distinct aberrations (lymphopenia, IFN, inflammatory cytokines), and, thereby, provides a numeric score, whereas subset membership groups patients with similar immunologic perturbations. Indeed, when patients were subsetted by the LuCIS value, it was possible to see differences in subsequent flare frequency and clinical response to therapy in a manner that was similar to that noted when the individual subsets were evaluated.

In the large cohort of adult lupus patients from the ILLUMINATE trials (GSE88884), the endotype with the least abnormal transcriptional profile manifested the lowest mean SLEDAI, lowest ANA titers, highest serum complement levels, and lowest incidence of lymphopenia, whereas the subsets with more abnormal transcriptional profiles showed significantly more abnormal clinical features. The endotype with the least abnormal transcriptional profile also had a significantly lower frequency of severe flares over the subsequent 52 weeks while receiving SoC medication and exhibited no significant clinical response to the investigational product, tabalumab. Moreover, we identified significant clinical responses to tabalumab in three endotypes with perturbed gene expression profiles. Notably, the effect sizes in these responsive subsets greatly exceeded that reported for responsiveness of the entire population (10.7 vs > 20%) [39]. Thus, we were able to identify clinically meaningful phenotypes based on the gene expression-based endotypes.

Several of the endotypes identified in the adult lupus patients were also found in pediatric patients, but a few transcriptionally distinct endotypes emerged among the pediatric patients that led to the final determination of eight global SLE molecular subsets. General patterns of clinical characterization were mirrored in the pediatric endotypes, with the least transcriptionally perturbed endotype exhibiting comparably low disease activity, and the endotype manifesting the highest disease activity being comprised of the fewest EA patients. However, hydroxychloroquine usage differed among endotypes, unlike in adults, and pediatric endotype membership did not depend on patient ancestry. This could have been reflective of overall higher proportions of non-EA ancestries in the pediatric patients.

Because gene expression can be influenced by the use of corticosteroids, immunosuppressive agents, or patient ancestry, we repeated our analyses in cohorts restricted to patients of single ancestries and separate cohorts of patients taking oral steroids and/or mycophenolate, which we have shown to significantly affect plasma cells and other gene expression [13]. Most endotypes were identified across ancestries, but we found different distributions of patients by ancestry among the endotypes; in particular, very few AA patients were found in the endotype with the least perturbed transcriptional profile, and, likewise, few EA patients were found in the subset with the greatest number of immunologic perturbations when all ancestries were considered. These findings suggest that these identified endotypes may be reflective of immunologic activity that presents clinically as more severe SLE among AA cohorts compared to EA cohorts [13, 43]. That is, the transcriptional profiling and subsequent endotyping presented herein may serve as a proxy for lupus immune activity.

We further illustrated this point by stratifying patients according to SoC medication and examining alterations in the distribution of patients among endotypes. When patients only taking oral steroids were considered, there was increased representation of a transcriptionally perturbed endotype; however, the addition of patients taking mycophenolate or methotrexate reduced the appearance of this endotype, confirming the clinical relevance of these drugs and the capacity of our endotype identification to detect these influences.

Additionally, we were able to demonstrate the utility of transcriptomic profiling over current, standard biomarkers, and patient demographics. Sub-setting lupus patients by *k*-means clustering of clinical data alone stratified patients primarily by ancestry and was not robust to methodology, as employment of clustering by a deep learning, variational autoencoder produced significantly different results, and both differed from the molecular subsets, a finding confirmed by ML. Thus, the molecular endotypes could not be identified based on clinical metadata.

There are limitations to our study including the generalizability of our findings. The majority of the patients analyzed were female, and one of the largest cohorts of our data, GSE88884, was restricted to patients selected for enrollment in a clinical trial and did not have active lupus nephritis or central nervous system involvement. Additionally, less than 5% of total patients analyzed were children, which may limit applicability of these endotypes to these patients. However, we included 137 pediatric patients as one of our formative datasets to identify the endotypes and we analyzed as many datasets as were available. Another possible limitation is the unknown clinical utility of LuCIS. Although there were significant correlations between LuCIS and SLEDAI, anti-dsDNA, and serum complement (negative), most correlations were modest. Additional real-world evidence will be necessary to determine the clinical utility of LuCIS. We anticipate future directions of this research to include analysis of longitudinal gene expression data, and we have indeed begun examining the fluidity of endotype membership among lupus patients over time in groups taking targeted therapies. Initial results show a tendency to normalize gene expression profiles following treatment with the cereblon binder, iberdomide [44].

In keeping with the shift of clinical medicine to greater emphasis on individualization and precision, we identified eight endotypes that map the molecular heterogeneity of individual lupus patients while also representing an entire disease population. Our body of work and the development of LuCIS based on these eight endotypes demonstrates how an individual patient can be classified using his or her blood gene expression profile. With the power of big data, bioinformatics, and ML, we anticipate this new method of clinical classification of lupus will aid physicians’ overall decision making, facilitating precise therapeutic courses of action and disease management strategies.

## Conclusions

We have developed a novel patient stratification methodology based on analysis of gene expression profiles of blood samples to identify endotypes (subsets) of patients with SLE. We leverage this information to develop a machine learning algorithm that effectively predicts endotype membership and also identifies the major molecular features driving subset membership. Finally, we have also developed a novel composite score defining gradations of immunologic perturbations, based on gene expression profiles. This composite score complements the endotypes and summarizes the extent of modular immunologic abnormalities, and may have both staging and prognostic relevance. These results provide the basis of precise identification of SLE patient endotypes and provide important novel information that should foster personalized care of patients with this heterogeneous disease.

### Supplementary Information

**Additional file 1:** This additional file consists of the following supplementary tables referenced throughout the manuscript: **Table S1.** Summary of transcriptional whole blood lupus datasets. **Table S2.** Comparison of clustering algorithms in GSE88884 ILL-1 & ILL-2. **Table S3.** GSVA enrichment scores and endotype membership of SLE patients. **Table S4.** Binary classifier performance metrics for eight lupus endotypes. **Table S5.** Contingency tables for severe flare calculation in placebo patients from GSE88884 ILL-1 & ILL-2. **Table S6.** Characteristics of clinically responsive endotypes to tabalumab in GSE88884 ILL-2. **Table S7.** Contingency tables for severe flare calculation in placebo patients from GSE88884 ILL-2.

**Additional file 2.** This additional file consists of the following supplemental figures referenced throughout the manuscript: **Figure S1.** Determination of k clusters. **Figure S2.** Experimental design of the process used to determine the final 32 modules for endotyping. **Figure S3.** Machine learning algorithms can predict lupus endotype membership with high accuracy. **Figure S4**. K-means clustering and comparison of lupus and control samples in GSE88884. **Figure S5**. K-means clustering of lupus and control samples. **Figure S6**. Eight molecular subsets in five formative SLE datasets. **Figure S7.** Modular immune dysregulation. **Figure S8.** Centroid based ML design to validate the classification of patients into final endotypes. **Figure S9.** Cosine similarity between A-H endotype designation and 8 clusters identified by k-means of the training/validation or testing cohorts. **Figure S10.** ML algorithms can predict lupus endotype membership with high accuracy. **Figure S11.** Unsupervised ML design to validate the classification of patients into final endotypes. **Figure S12.** All 26 features are required for endotype identification. **Figure S13.** ML-Predicted endotypes in male SLE patients. **Figure S14.** One-vs-rest multi-class classification of lupus endotype memberships. **Figure S15.** Summary Plot of SHAP values for XGB multi-class ML model. **Figure S16.** Distinguishment of endotype B from A. **Figure S17.** Distinguishment of endotype C from A. **Figure S18.** Distinguishment of endotype D from A. **Figure S19.** Distinguishment of endotype E from A. **Figure S20.** Distinguishment of endotype F from A. **Figure S21.** Distinguishment of endotype G from A. **Figure S22.** Distinguishment of endotype H from A. **Figure S23.** Gini index analysis reveals features most distinctive of transcriptional perturbations in the seven abnormal lupus endotypes. **Figure S24.** Lupus subsets derived from clinical features. **Figure S25.** Machine learning prediction of molecular endotype memberships using clinical metadata as features. **Figure S26.** Endotype reassignment in GSE88884. **Figure S27.** Clinical characterization of SLE endotypes. **Figure S28.** SLEDAI manifestations among six adult lupus endotypes in GSE88884. **Figure S29.** Organ system involvement among six adult lupus endotypes in GSE88884. **Figure S30.** Clinical characteristics of five pediatric lupus endotypes. **Figure S31.** Endotypes of EA adult SLE patients. **Figure S32.** Endotypes of AA adult SLE patients. **Figure S33.** Endotypes of NAA adult SLE patients. **Figure S34.** Distribution of patients in GSE88884 drug treatment cohorts. **Figure S35.** Endotypes in GSE88884 ILL-2. **Figure S36.** Clinical characterization of SLE endotypes in GSE88884 ILL-2.

## Data Availability

The datasets analyzed during the study are publicly available in the GEO repository: GSE88884 (https://www.ncbi.nlm.nih.gov/geo/query/acc.cgi?acc=GSE88884) [39] GSE45291 (https://www.ncbi.nlm.nih.gov/geo/query/acc.cgi?acc=GSE45291) [45] GSE65391 (https://www.ncbi.nlm.nih.gov/geo/query/acc.cgi?acc=GSE65391) [9] GSE116006 (https://www.ncbi.nlm.nih.gov/geo/query/acc.cgi?acc=GSE116006) [46] GSE22098 (https://www.ncbi.nlm.nih.gov/geo/query/acc.cgi?acc=GSE22098) [47] GSE29536 (https://www.ncbi.nlm.nih.gov/geo/query/acc.cgi?acc=GSE29536) [48] GSE39088 (https://www.ncbi.nlm.nih.gov/geo/query/acc.cgi?acc=GSE39088) [49] GSE49454 (https://www.ncbi.nlm.nih.gov/geo/query/acc.cgi?acc=GSE49454) [14] GSE61635 (https://www.ncbi.nlm.nih.gov/geo/query/acc.cgi?acc=GSE61635) [50] GSE72509 (https://www.ncbi.nlm.nih.gov/geo/query/acc.cgi?acc=GSE72509) [51] GSE72747 (https://www.ncbi.nlm.nih.gov/geo/query/acc.cgi?acc=GSE72747) [52] GSE110174 (https://www.ncbi.nlm.nih.gov/geo/query/acc.cgi?acc=GSE110174) [53] GSE112087 (https://www.ncbi.nlm.nih.gov/geo/query/acc.cgi?acc=GSE112087) [20] GSE138458 (https://www.ncbi.nlm.nih.gov/geo/query/acc.cgi?acc=GSE138458) [15] GSE185047 (https://www.ncbi.nlm.nih.gov/geo/query/acc.cgi?acc=GSE185047) [54] or the BioProject repository: PRJNA717024 (SRX10438277) (https://www.ncbi.nlm.nih.gov/bioproject/PRJNA717024) [55] The R and Python scripts for data preprocessing, GSVA, k-means clustering, ML and SHAP analysis are available on GitHub: https://github.com/pbachali16/ML-reveals-endotypes-in-lupus-samples-using-transcriptomic-features [44]

## Abbreviations

AA: African ancestry
ACR: American College of Rheumatology
EA: European ancestry
GB: Gradient boosting
gd T Cell: Gamma/delta T cell
GFR: Glomerular filtration rate
GSVA: Gene set variation analysis
IFN: Interferon
IG: Immunoglobulin
ILL: ILLUMINATE
ISM: Interferon signature metric
IQR: Interquartile range
LDG: Low-density granulocyte
LR: Logistic regression
LuCIS: *Lu*Pus *C*ell and *I*mmune *S*core
MHCII: Major histocompatibility complex II
ML: Machine learning
NAA: Native American ancestry
PCA: Principal component analysis
PR: Precision recall
RF: Random forest
RNA-seq: RNA sequencing
ROC: Receiver operating characteristic
RPLR: Ridge-penalized logistic regression
SHAP: SHapley Additive ExPlanations
SLE or lupus: Systemic lupus erythematosus
SLEDAI: Systemic Lupus Erythematosus Disease Activity Index
SLICC: Systemic Lupus International Collaborating Clinics
SoC: Standard of care
SRI: SLE responder index
SVM: Support vector machine
TNF: Tumor necrosis factor
t-SNE: T-Distributed Stochastic Neighbor Embedding
XGB: Extreme gradient boosting
