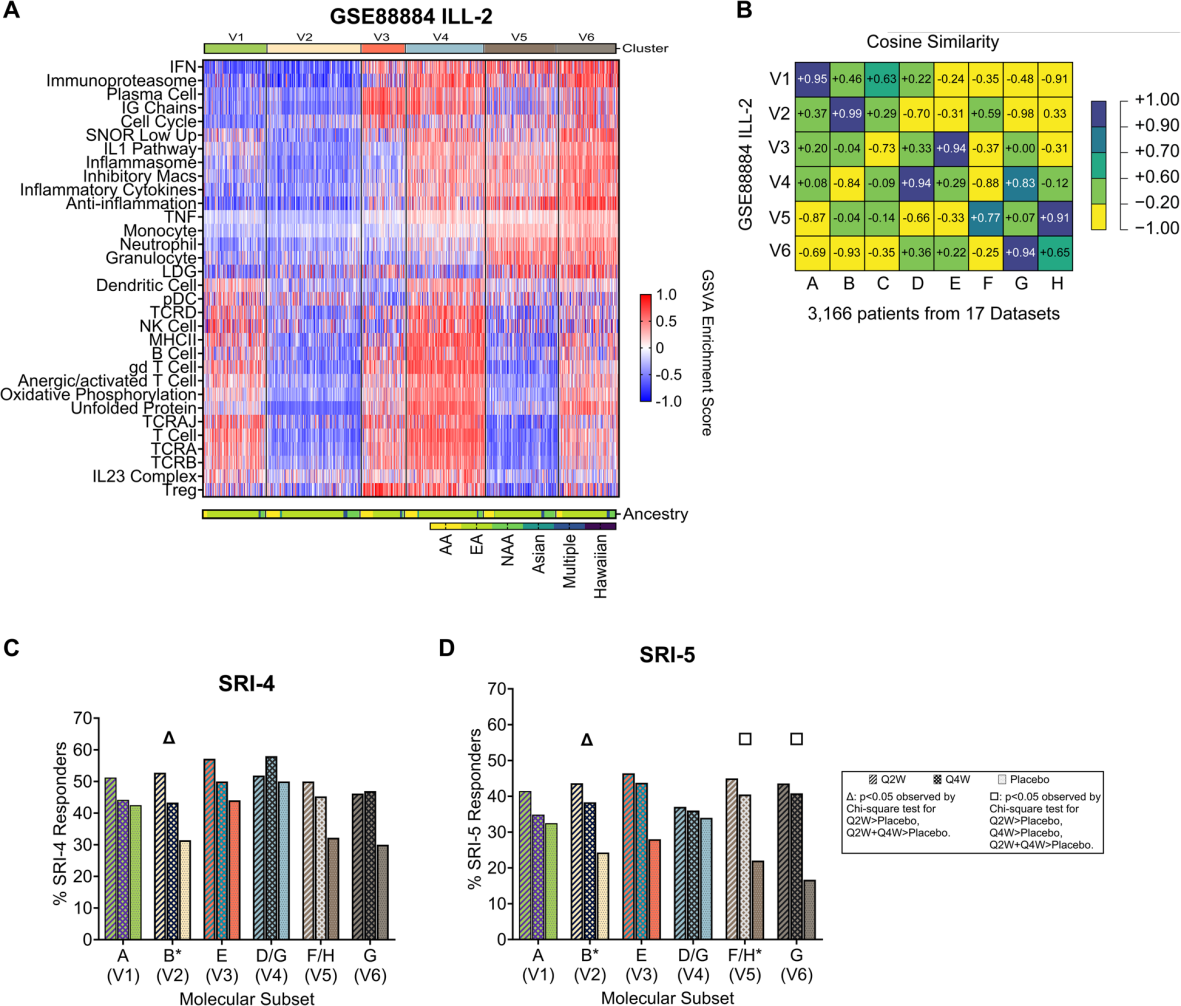# Publisher Correction: Analysis of transcriptomic features reveals molecular endotypes of SLE with clinical implications

**DOI:** 10.1186/s13073-023-01251-x

**Published:** 2023-12-13

**Authors:** Erika L. Hubbard, Prathyusha Bachali, Kathryn M. Kingsmore, Yisha He, Michelle D. Catalina, Amrie C. Grammer, Peter E. Lipsky

**Affiliations:** 1 AMPEL BioSolutions, LLC, 250 W. Main St. #300, Charlottesville, VA 22902 USA; 2 RILITE Research Institute, Charlottesville, VA 22902 USA; 3 Altria, Richmond, VA 23230 USA; 4 AbbVie, Worcester, MA 01605 USA

**Publisher Correction: Genome Med 15, 84 (2023)**

**https://doi.org/10.1186/s13073-023-01237-9**

Figure 5 in the original publication of this article [1] was incorrectly cut-off during the publication process. The incorrect and correct version of Fig. 5 are shown in this correction article. Furthermore, in figure 7 the annotation bars above and below the heatmap were not properly represented, the correct version of figure 7 is shown in this correction article. The original article has been updated. The publisher apologizes for the inconvenience caused to authors & readers.

**Incorrect figure**


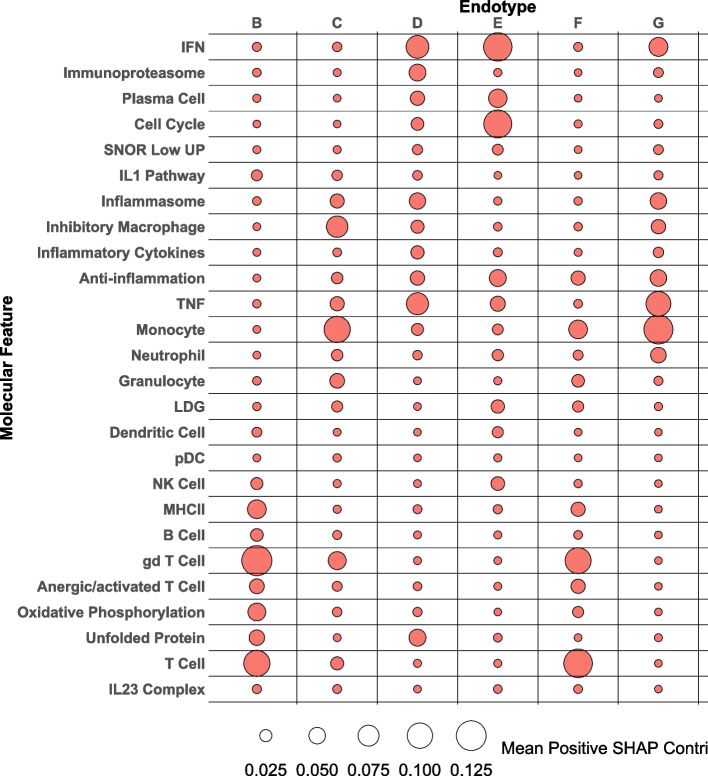


**Correct figure**


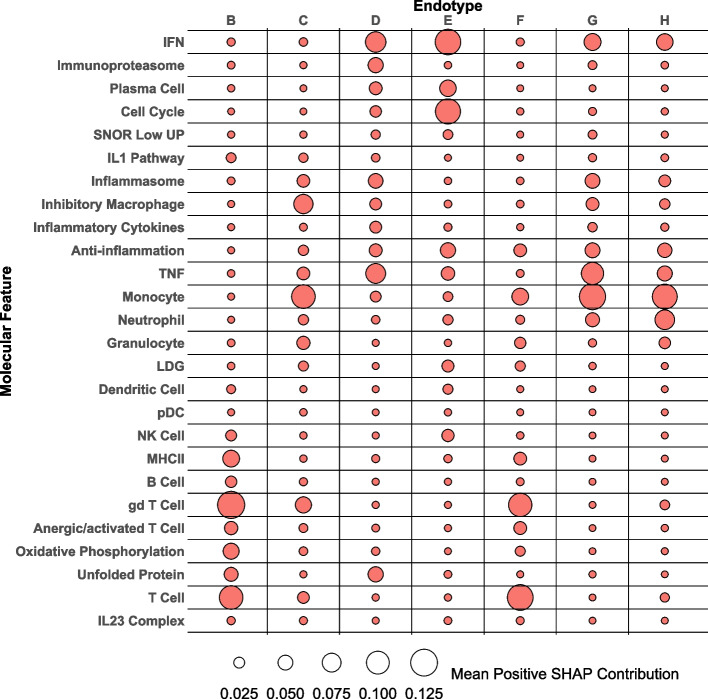


**Fig. 7**