# Supplementary material for: Analysis of transcriptomic features reveals molecular endotypes of SLE with clinical implications
Source: Genome Med. 2023 Oct 16;15:84. doi: 10.1186/s13073-023-01237-9 (PMC10578040; doi:10.1186/s13073-023-01237-9)
Supplement: Supplementary file 2 — Additional file 2. This additional file consists of the following supplemental figures referenced throughout the manuscript: Figure S1. Determination of k clusters. Figure S2. Experimental design of the process used to determine the final 32 modules for endotyping. Figure S3. Machine learning algorithms can predict lupus endotype membership with high accuracy. Figure S4. K-means clustering and comparison of lupus and control samples in GSE88884. Figure S5. K-means clustering of lupus and control samples. Figure S6. Eight molecular subsets in five formative SLE datasets. Figure S7. Modular immune dysregulation. Figure S8. Centroid based ML design to validate the classification of patients into final endotypes. Figure S9. Cosine similarity between A-H endotype designation and 8 clusters identified by k-means of the training/validation or testing cohorts. Figure S10. ML algorithms can predict lupus endotype membership with high accuracy. Figure S11. Unsupervised ML design to validate the classification of patients into final endotypes. Figure S12. All 26 features are required for endotype identification. Figure S13. ML-Predicted endotypes in male SLE patients. Figure S14. One-vs-rest multi-class classification of lupus endotype memberships. Figure S15. Summary Plot of SHAP values for XGB multi-class ML model. Figure S16. Distinguishment of endotype B from A. Figure S17. Distinguishment of endotype C from A. Figure S18. Distinguishment of endotype D from A. Figure S19. Distinguishment of endotype E from A. Figure S20. Distinguishment of endotype F from A. Figure S21. Distinguishment of endotype G from A. Figure S22. Distinguishment of endotype H from A. Figure S23. Gini index analysis reveals features most distinctive of transcriptional perturbations in the seven abnormal lupus endotypes. Figure S24. Lupus subsets derived from clinical features. Figure S25. Machine learning prediction of molecular endotype memberships using clinical metadata as features. Figure S26. End [file 13073_2023_1237_MOESM2_ESM.pdf]

GSE88884

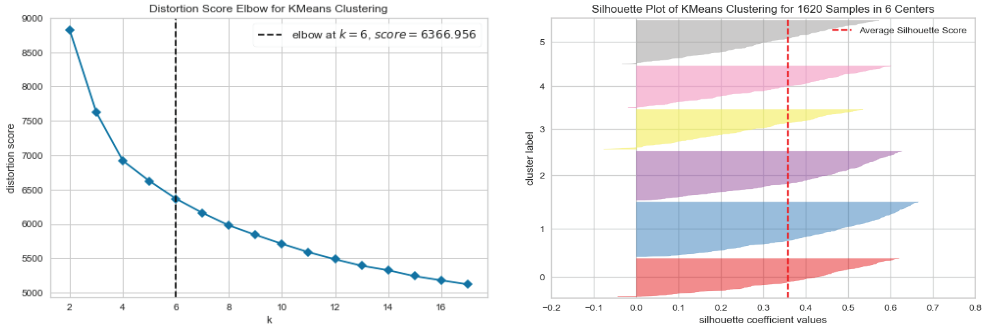

GSE45291

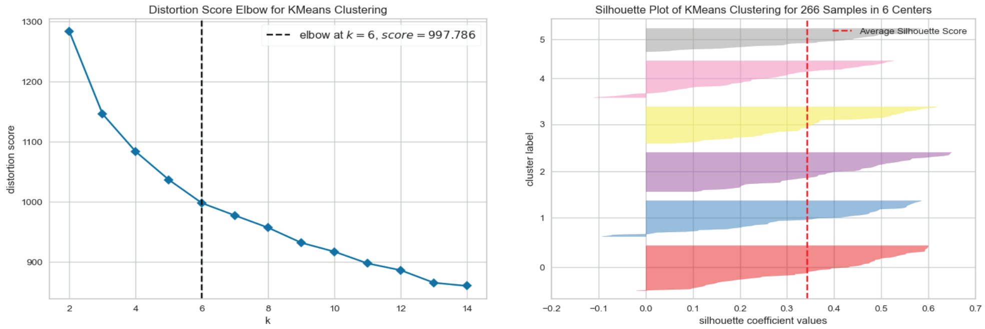

GSE65391

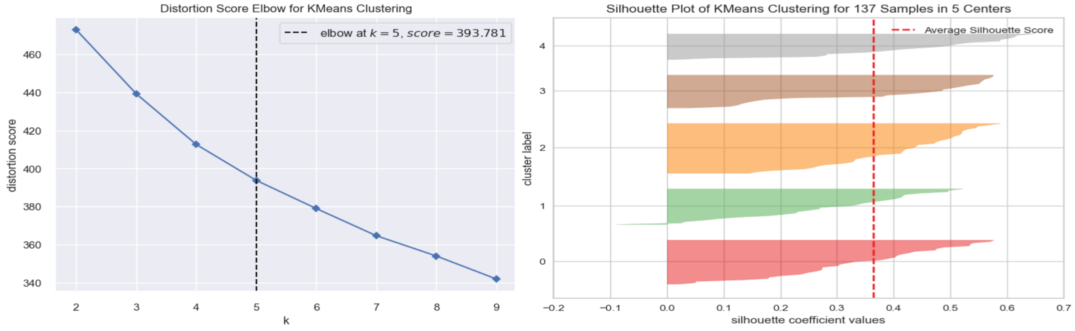

GSE116006

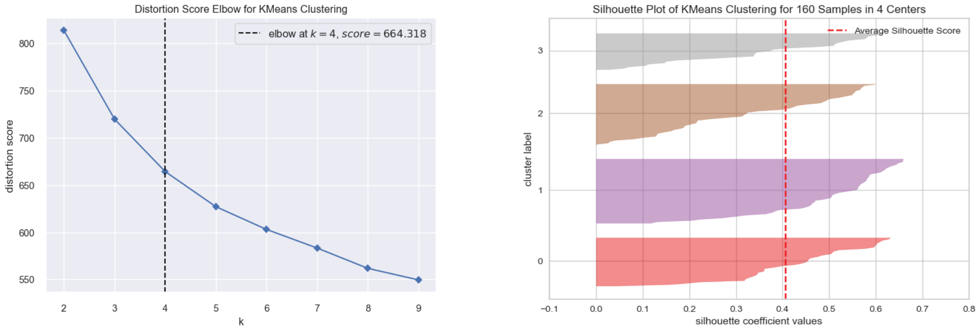

Figure S1. Determination of k clusters.
For each individually endotyped dataset, the Elbow method and Silhouette analysis were used to determine the optimal number of clusters, or endotypes, using KElbowVisualizer. The distortion score (sum of squared distances from each point to its center) of lupus samples is plotted on y-axis and the range of k values on x-axis. Once k was determined, Silhouette analysis on PCA components was conducted and Silhouette plots visualized to confirm the appropriateness of clustering. Silhouette plots were interpreted by average score across all samples, where 1 represents clearly distinguished clusters (best) and -1 represents poorly formed clusters (worst), and by the peaks of each cluster surpassing the average Silhouette score. Datasets are identified by their respective GEO accession number. Plots were generated in Python with matplotlib

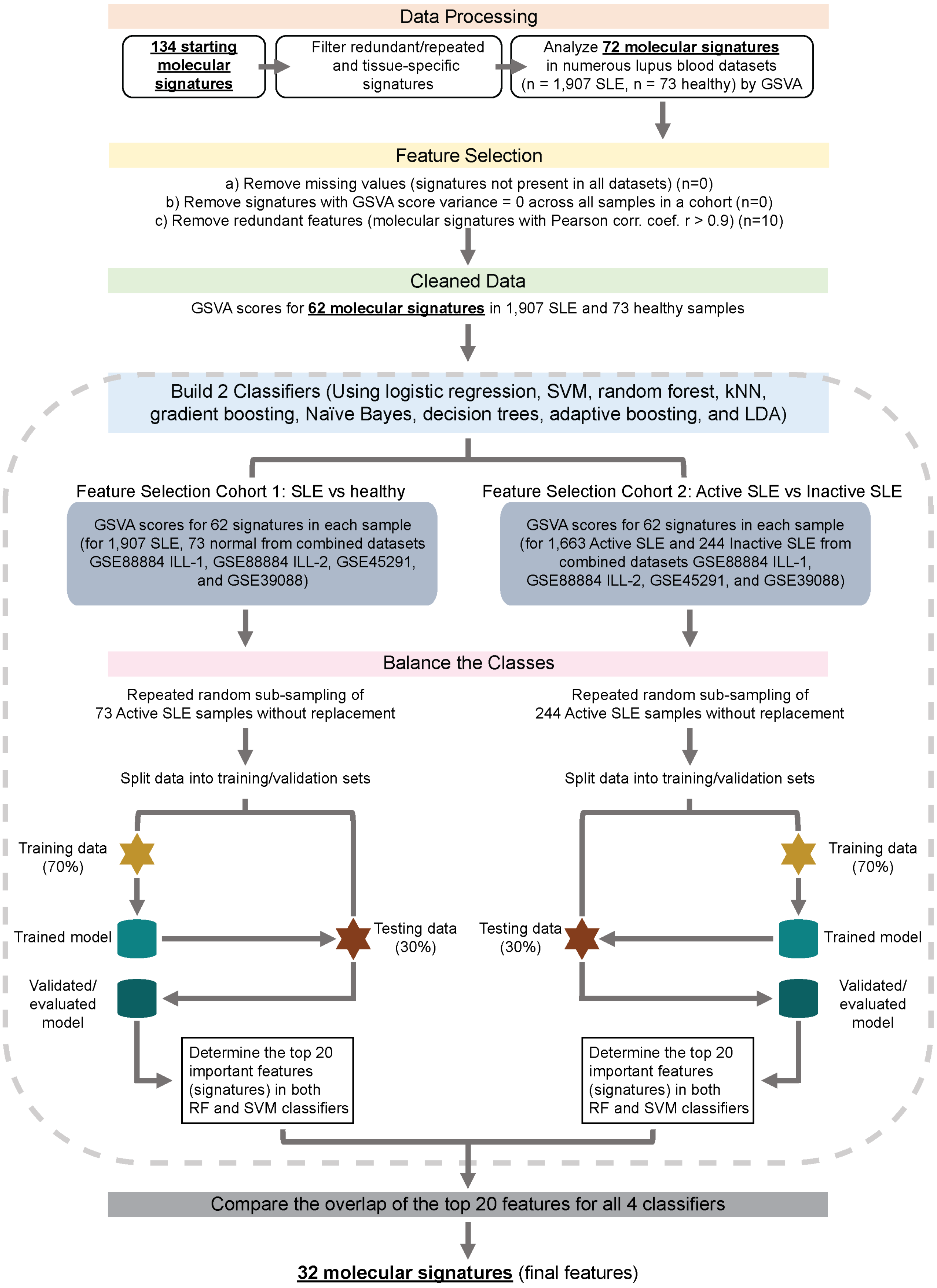

Figure S2. Experimental design of the process used to determine the final 32 modules for endotyping.
Data processing and machine learning workflow to arrive at the 32 features used to stratify patients for the identification of endotypes. Flow diagram created in Adobe Illustrator.

A Logistic regression classification of the eight endotypes of lupus

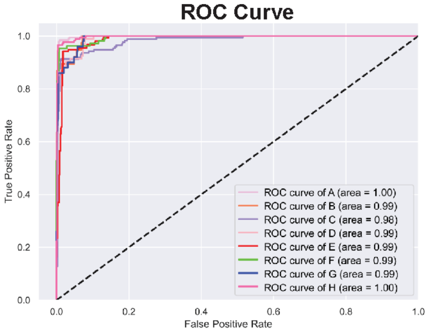

	Sensitivity	Specificity	Precision	Accuracy	f1-score
A	0.97	0.99	0.95	0.99	0.96
B	1.00	1.00	1.00	1.00	1.00
C	0.94	0.99	0.94	0.99	0.94
D	0.94	0.99	0.96	0.99	0.95
E	0.89	0.99	0.93	0.98	0.91
F	0.95	1.00	0.97	0.99	0.96
G	0.99	0.99	0.95	0.99	0.97
H	0.98	1.00	0.98	1.00	0.98

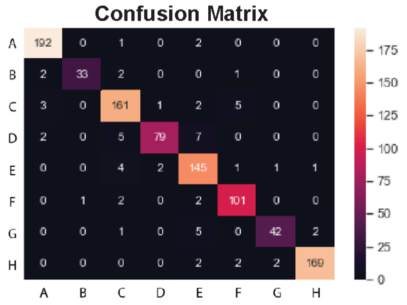

B Gradient boosting machines classification of the eight endotypes of lupus

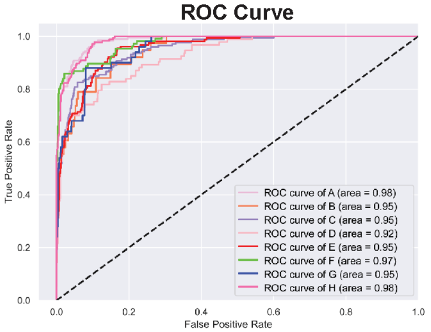

	Sensitivity	Specificity	Precision	Accuracy	f1-score
A	0.80	0.97	0.84	0.95	0.82
B	0.84	0.98	0.89	0.97	0.86
C	0.76	0.96	0.72	0.94	0.74
D	0.92	0.97	0.82	0.97	0.87
E	0.71	0.97	0.76	0.95	0.73
F	0.81	0.96	0.65	0.95	0.72
G	0.89	0.99	0.94	0.97	0.91
H	0.87	0.99	0.95	0.97	0.91

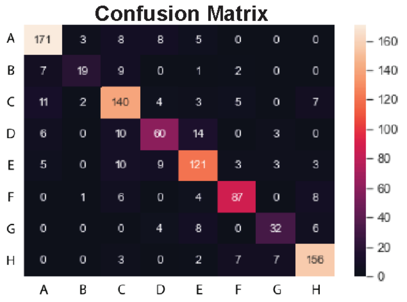

Figure S3. Machine learning algorithms can predict lupus endotype membership with high accuracy. Multi-class classification by ML categorized 3,166 lupus patients from 17 datasets into eight patient endotypes. Area under the ROC curve (AUC), performance metrics, and confusion matrices for 2/4 classifiers on the testing cohort data (983 samples) are summarized: (A) logistic regression and (B) gradient boosting. Each model was trained on 1,746 lupus samples, validated with 437 lupus samples, and tested on the remaining 983 samples for a total n=3,166 from 17 datasets. Plots were generated in Python using the scikit-learn and matplotlib libraries.

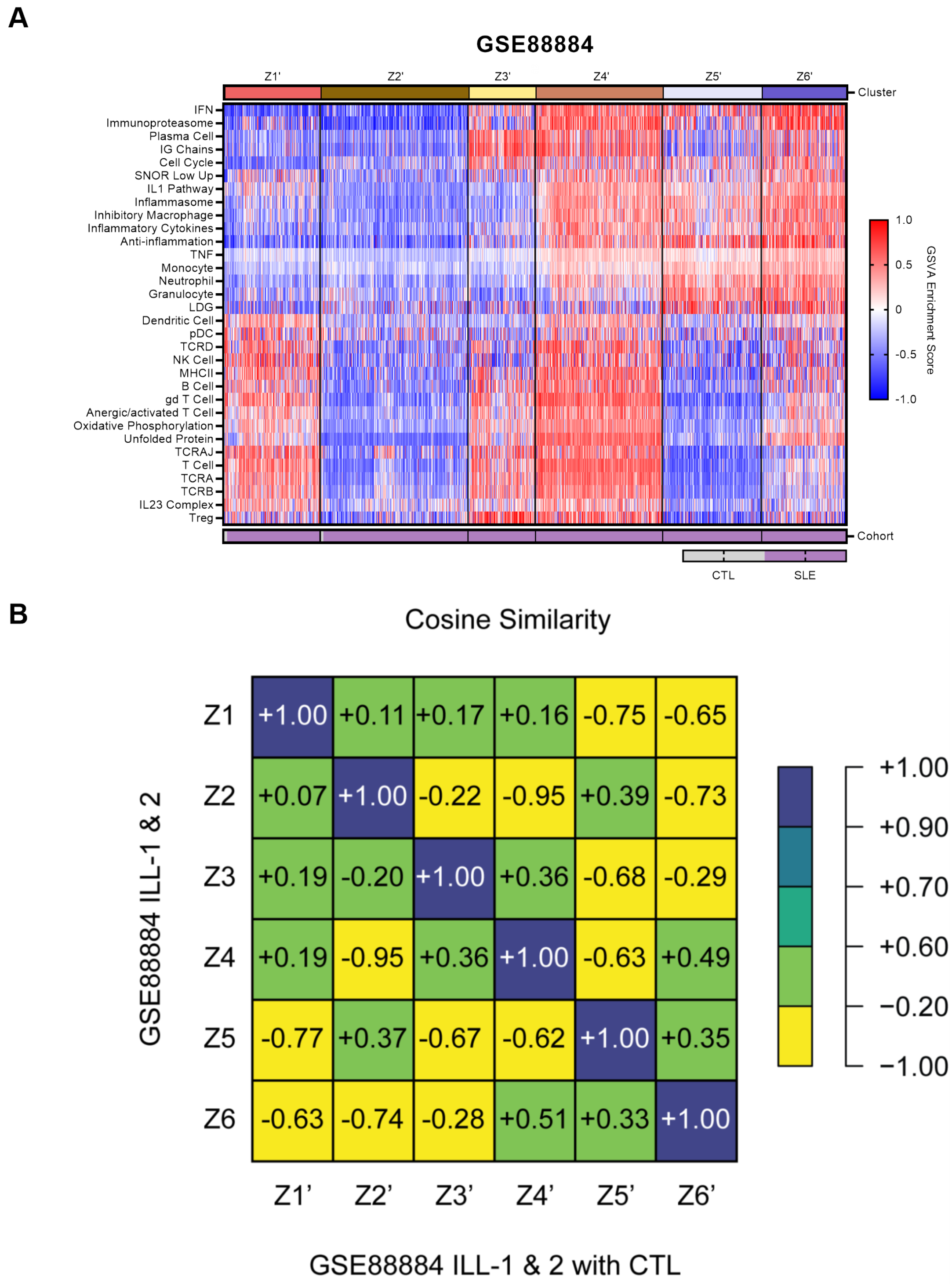

Figure S4. K-means clustering and comparison of lupus and control samples in GSE88884.
(A) K-means clustering of GSVA scores of the 32 features in 1,620 lupus patients and 17 healthy controls (CTLs) in GSE88884. 64.7% of the controls were found in Z1’ (Z1’ = 11, Z2’ = 6). (B) Cosine similarity of the k-means clusters of the 1,620 lupus patients only versus patients and controls. Heatmap in (A) was generated with GraphPad Prism v. 9.4.0 (673). Cosine similarity plot in (B) was generated in R with the plot.matrix package and edited in Adobe Illustrator.

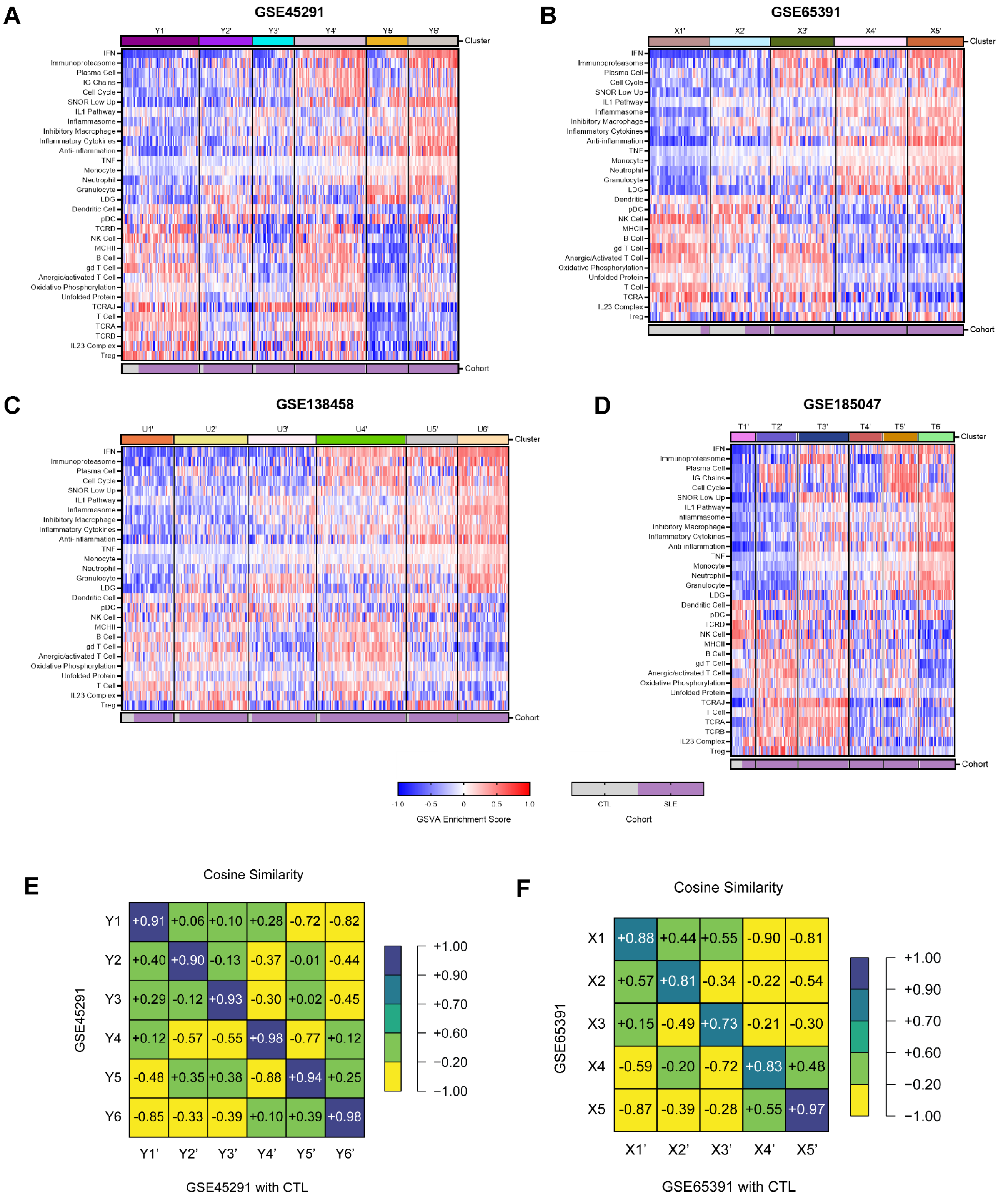

Figure S5. K-means clustering of lupus and control samples.

K-means clustering of GSVA scores of the 32 features in lupus patients and controls (CTLs) in (A) GSE45291 (266 SLE and 20 CTL), (B) GSE65391 (137 SLE and 57 CTL), (C) GSE138458 (307 SLE and 23 CTL), and (D) GSE185047 (177 SLE and 10 CTL). Of note, only 28 and 27 features were used in GSE65391 and GSE138458 respectively because of the microarray chip restrictions. Endotypes in GSE116006 were featured Figure 1 but did not contain control samples, thus datasets in (C) and (D) were clustered with controls to further illustrate the assignation of “abnormally enriched” endotypes. The control samples in GSE138458 were patients with non-autoimmune rheumatic diseases recruited from health fairs, whereas in the other datasets controls were healthy. In (A), 75% of controls were found in Y1’ (Y1’ = 15 CTLs, Y2’ = 3 CTLs, Y3’ = 2 CTLs). In (B), 57.9% of controls were found in X1’ (X1’ = 33 CTLs, X2’ = 22 CTLs, X3’ = 2 CTLs). In (C), 47.8% of controls were found in U1’ (U1’ = 11 CTLs, U2’ = 4 CTLs, U3’ = 3 CTLs, U4’ = 3 CTLs, U5’ = 2 CTLs). In (D), all 10 controls were found in T1’. Heatmaps in (A-D) were generated with GraphPad Prism v. 9.4.0 (673). Cosine similarity of k-means clusters identified in SLE patients alone versus SLE + CTLs in (E) GSE45291 and (F) GSE65391. The cosine similarity plots in (E-F) were generated in R with the plot.matrix package and edited in Adobe Illustrator.

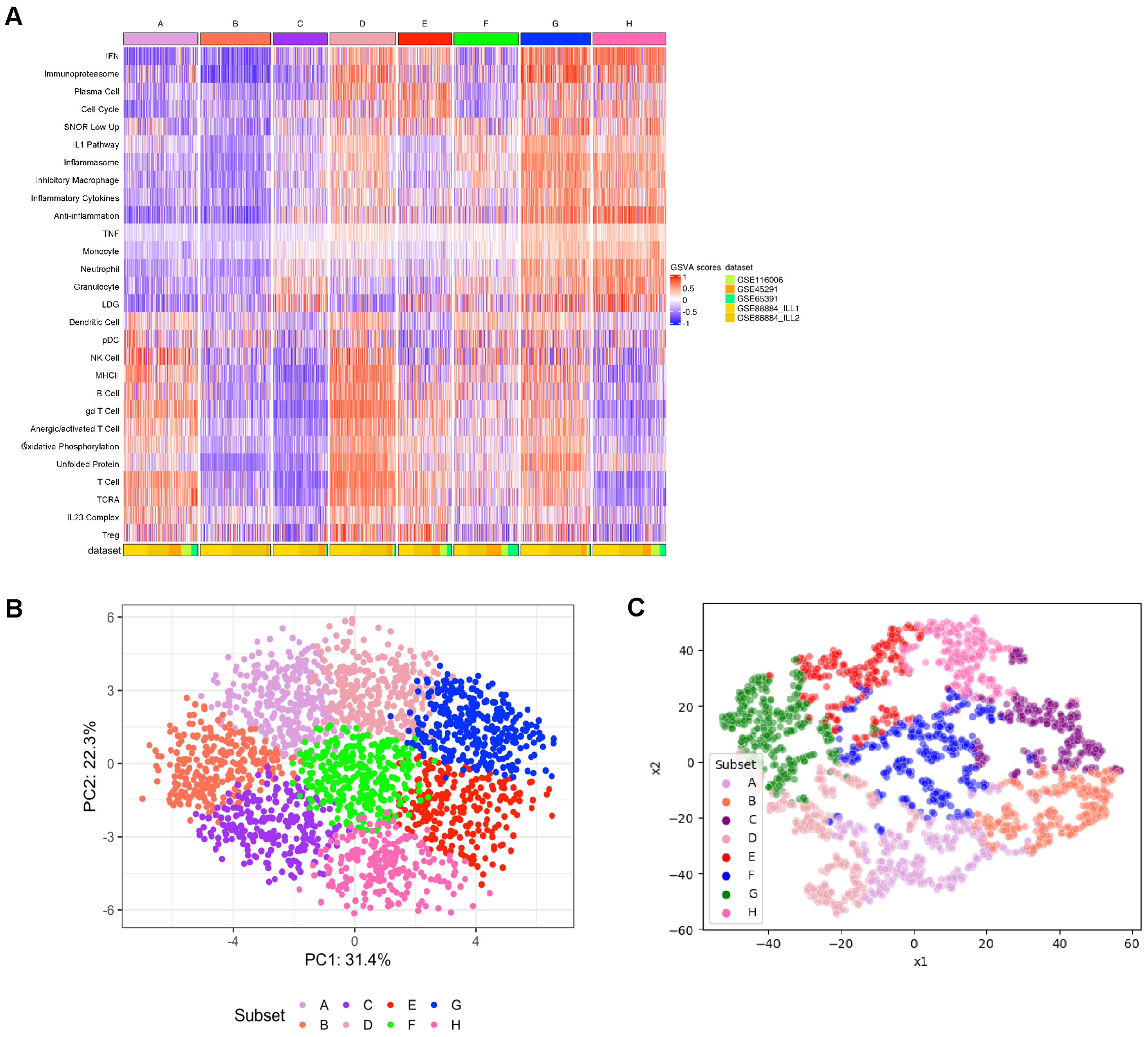

Figure S6. Eight molecular subsets in five formative SLE datasets.
(A) Heatmap visualization of eight lupus subsets identified by k-means clustering applied on 2,183 SLE samples. The gene expression data from five datasets was analyzed. GSVA scores of 2,183 samples using 28 informative gene modules were passed to k-means clustering technique to identify the lupus subsets. The lupus subsets are ordered from “least abnormal” to “most abnormal” immunologic activity. (B) PCA was conducted on 2,183 * 28 modules and the first two components were visualized. (C) The 2,183 patients from five datasets were also visualized with t-distributed stochastic neighbor embedding (t-SNE). Labeling of clusters in (B) and (C) was done using cluster memberships determined by (A). Heatmap in (A) was generated in R with ComplexHeatmap. The PCA plot in (B) with stats and ggplot and the t-SNE plot was generated in in Python with scikit-learn.

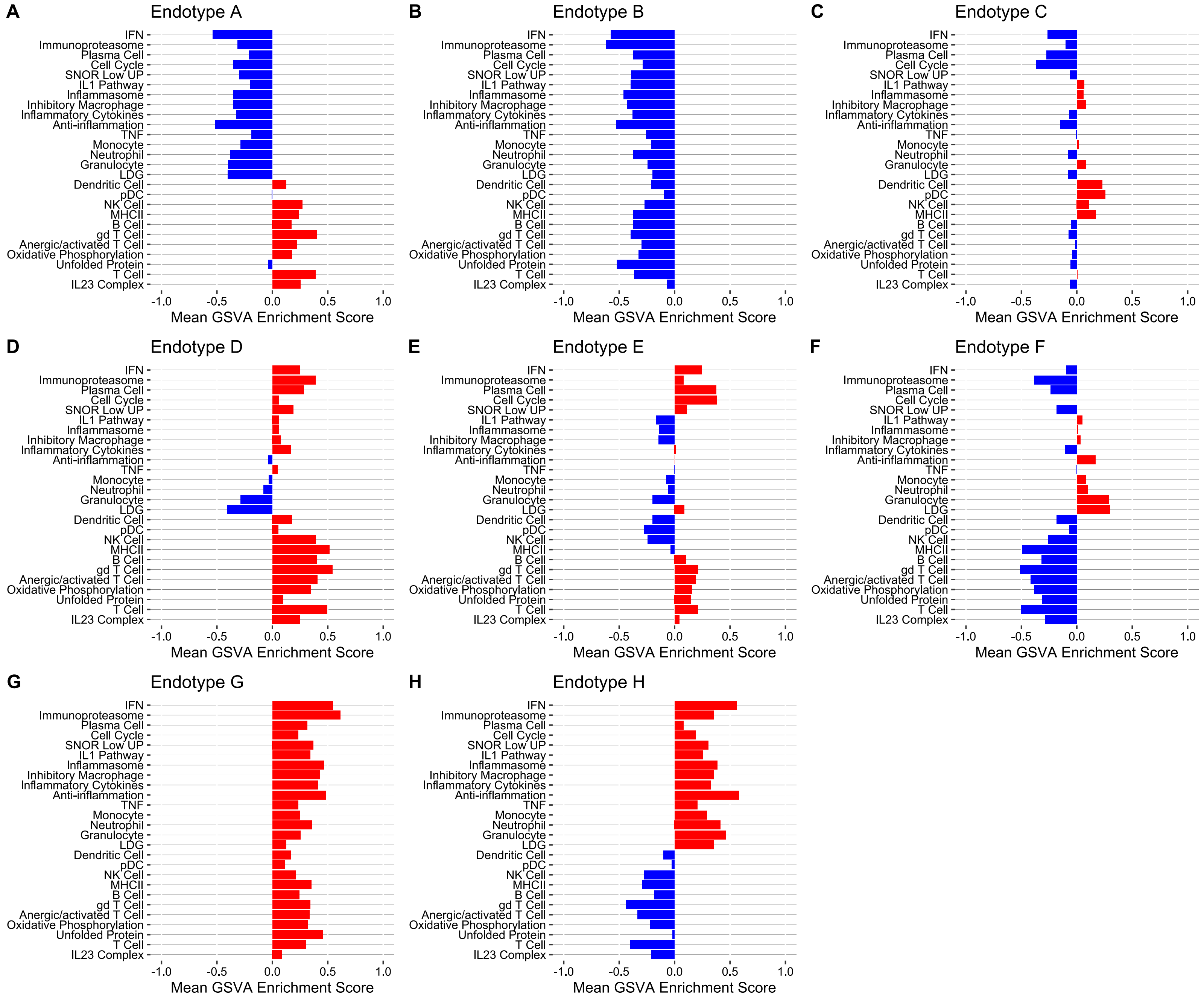

Figure S7. Modular immune dysregulation.

Mean GSVA scores for 26/32 features for each endotype in 3,166 SLE samples were visualized as bar plots to demonstrate the extent of immune dysregulation. The red bars represent positive enrichment scores and blue represent negative enrichment scores. Bar plots were generated in R with the ggplot2 package.

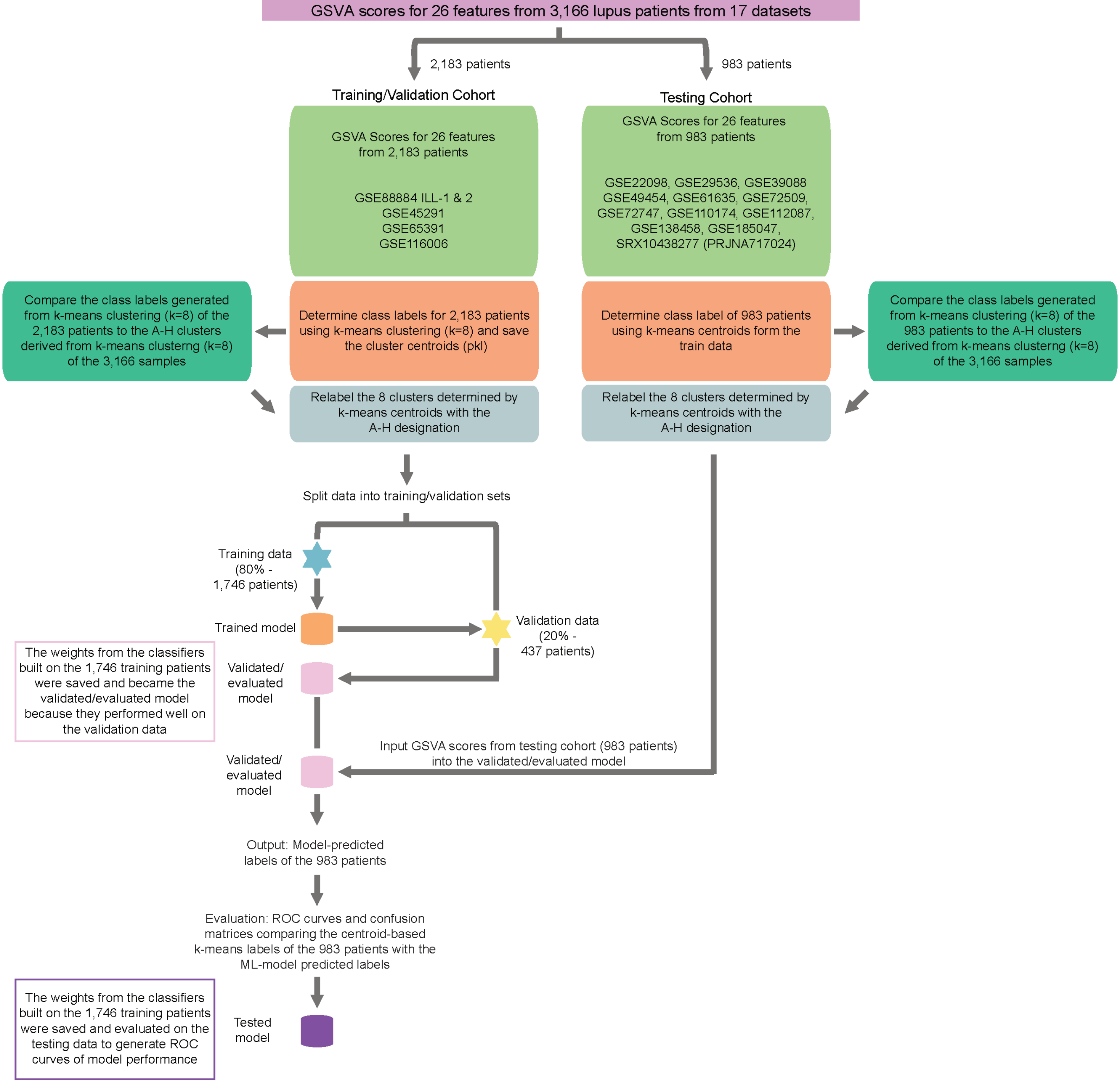

Figure S8. Centroid-based ML design to validate the classification of patients into final endotypes. As an alternate approach to confirm the validity of ML models shown in the main text, an additional ML design was carried out. For development and validation of ML models (shown in Figure S10), the 3,166 patients were split into a training/validation cohort (five datasets, n=2,183; training (n=1,746), validation (n=437)) and a testing cohort (12 datasets, n=983). The class labels of the 2,183 patients were generated using k-means clustering (k=8). The weights from the k-means clustering centroids were saved. Then, the class labels of the 983 patients were independently generated using k-means clustering (k=8) with the saved weights. Class labels for both cohorts were relabeled according to their cosine similarity with A-H, so that nomenclature would be consistent (Figure S9). These identified endotypes were used as the class label for subsequent ML. The performance of ML classifiers built on the training data was evaluated using the validation samples, and the performance was satisfactory. Thus, the ML model weights from the training data were set as the final ML model weights. The final performance of the classifiers was evaluated using the test data (n=983). The ROC curves, performance metrics, and confusion matrices were made for the 983 samples. Flow diagram created in Adobe Illustrator.

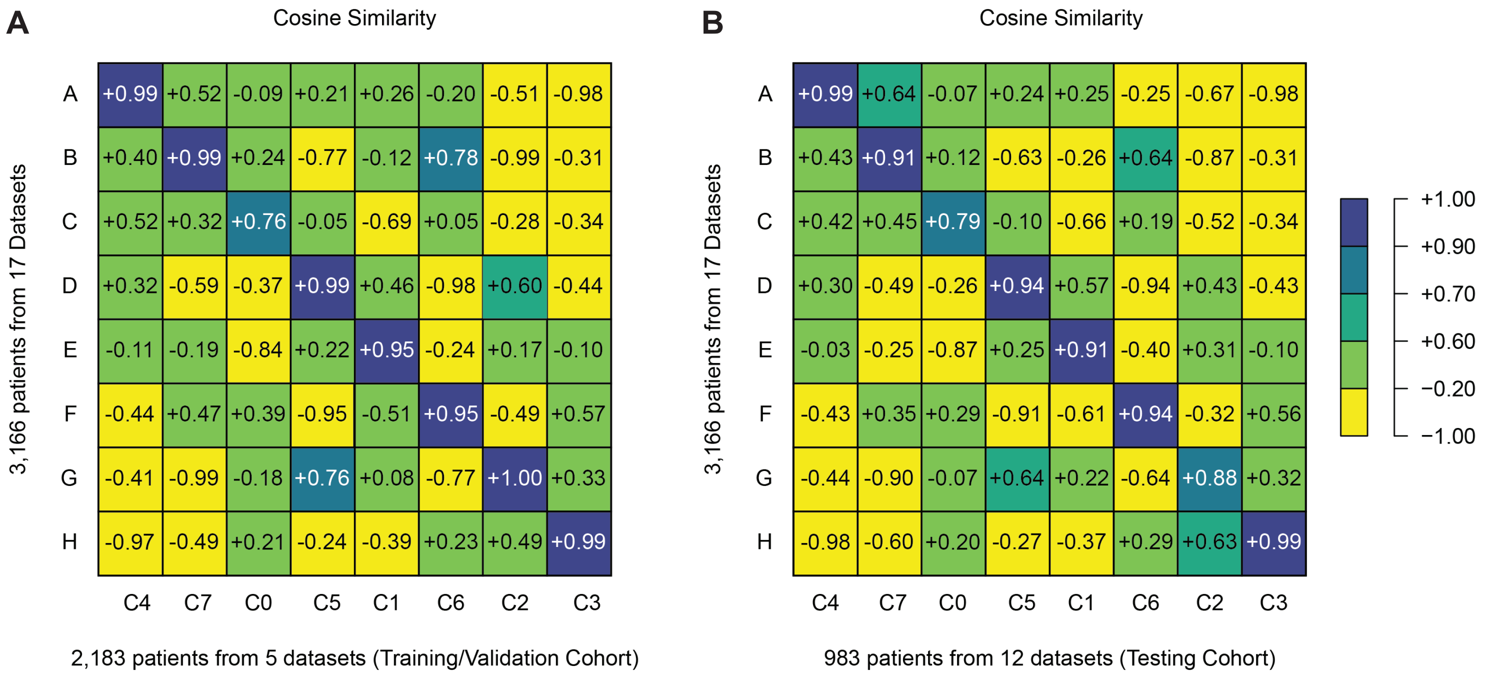

Figure S9. Cosine similarity between A-H endotype designation and 8 clusters identified by k-means of the training/validation or testing cohorts.
Cosine similarity between the A-H labels (generated from k-means clustering of the 3,166 patients from 17 datasets, see Figure S8) and (A) the training/validation cohort of 2,183 patients from five datasets or (B) the testing cohort of 983 patients from 12 datasets was used to designate the independently derived clusters as A-H, so that class label in the alternate ML design would be consistent. The cosine similarity plots were generated in R with the plot.matrix package and edited in Adobe Illustrator.

A

Random forest classification of the eight endotypes of lupus

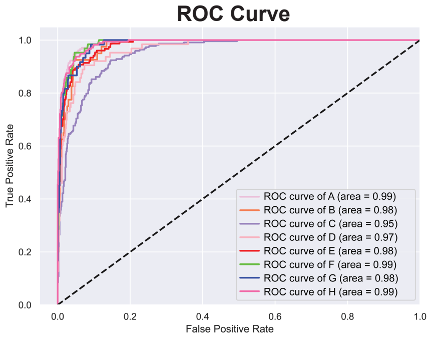

	Sensitivity	Specificity	Precision	Accuracy	f1-score
**A**	0.82	0.98	0.89	0.96	0.85
**B**	0.81	0.98	0.85	0.96	0.83
**C**	0.92	0.97	0.81	0.96	0.86
**D**	0.89	0.98	0.89	0.97	0.89
**E**	0.95	0.98	0.82	0.98	0.88
**F**	0.75	0.99	0.84	0.97	0.79
**G**	0.89	0.99	0.92	0.97	0.90
**H**	0.92	0.99	0.94	0.98	0.93

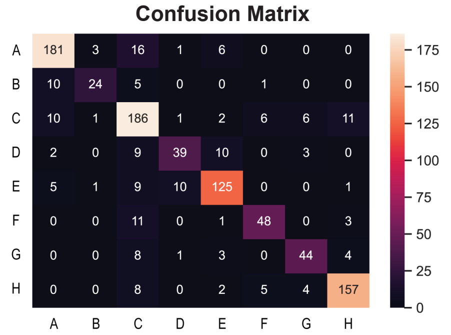

B

Support vector machine classification of the eight endotypes of lupus

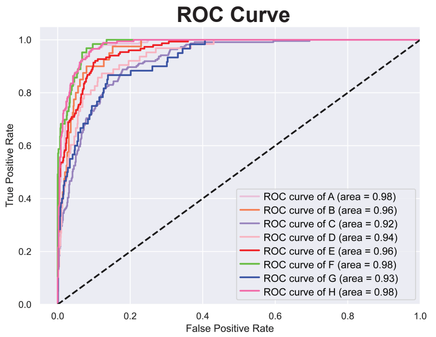

	Sensitivity	Specificity	Precision	Accuracy	f1-score
**A**	0.71	0.96	0.77	0.92	0.74
**B**	0.67	0.98	0.84	0.95	0.75
**C**	0.75	0.96	0.74	0.93	0.74
**D**	0.72	0.97	0.80	0.94	0.76
**E**	0.82	0.95	0.62	0.94	0.71
**F**	0.78	0.98	0.74	0.96	0.76
**G**	0.82	0.96	0.81	0.94	0.81
**H**	0.92	0.97	0.85	0.97	0.88

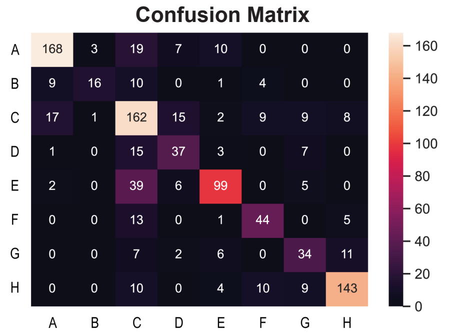

C

Logistic regression classification of the eight endotypes of lupus

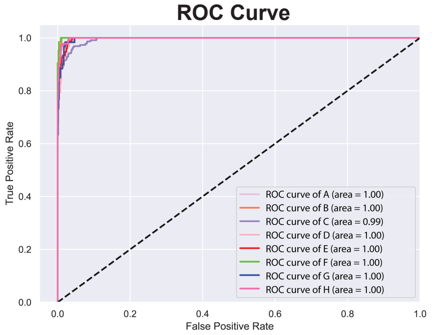

	Sensitivity	Specificity	Precision	Accuracy	f1-score
**A**	0.96	0.99	0.97	0.99	0.96
**B**	0.90	0.99	0.96	0.98	0.93
**C**	0.87	0.99	0.93	0.97	0.90
**D**	0.98	1.00	1.00	1.00	0.99
**E**	0.97	0.99	0.90	0.99	0.93
**F**	0.97	0.99	0.88	0.99	0.92
**G**	1.00	0.99	0.96	0.99	0.98
**H**	0.97	1.00	0.98	0.99	0.97

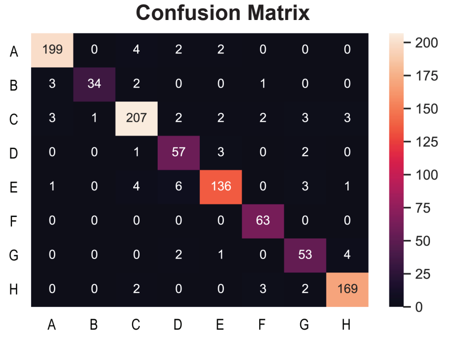

D

Gradient boosting machines classification of the eight endotypes of lupus

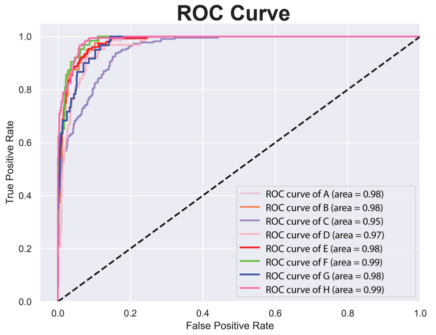

	Sensitivity	Specificity	Precision	Accuracy	f1-score
**A**	0.81	0.98	0.86	0.95	0.83
**B**	0.73	0.98	0.80	0.95	0.76
**C**	0.88	0.95	0.76	0.95	0.82
**D**	0.89	0.98	0.85	0.97	0.87
**E**	0.82	0.98	0.80	0.97	0.81
**F**	0.78	0.98	0.80	0.97	0.79
**G**	0.88	0.98	0.89	0.97	0.88
**H**	0.89	0.99	0.95	0.98	0.92

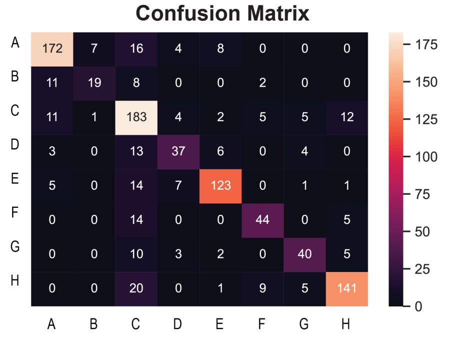

Figure S10. ML algorithms can predict lupus endotype membership with high accuracy
Centroid-based multi-class ML models classified 3,166 lupus patients from 17 datasets into eight patient endotypes, per the workflow described in Figure S8. Area under the ROC curve (AUC), performance metrics, and confusion matrices for each of four classifiers on the testing cohort data (983 samples) are summarized: (A) random forest, (B) support vector machine, (C) logistic regression, and (D) gradient boosting. Each model was trained on 1,746 lupus samples, validated with 437 lupus samples, and tested on the remaining 983 samples for a total n=3,166 from 17 datasets. Plots in (A-D) were generated in Python with the scikit-learn and matplotlib libraries.

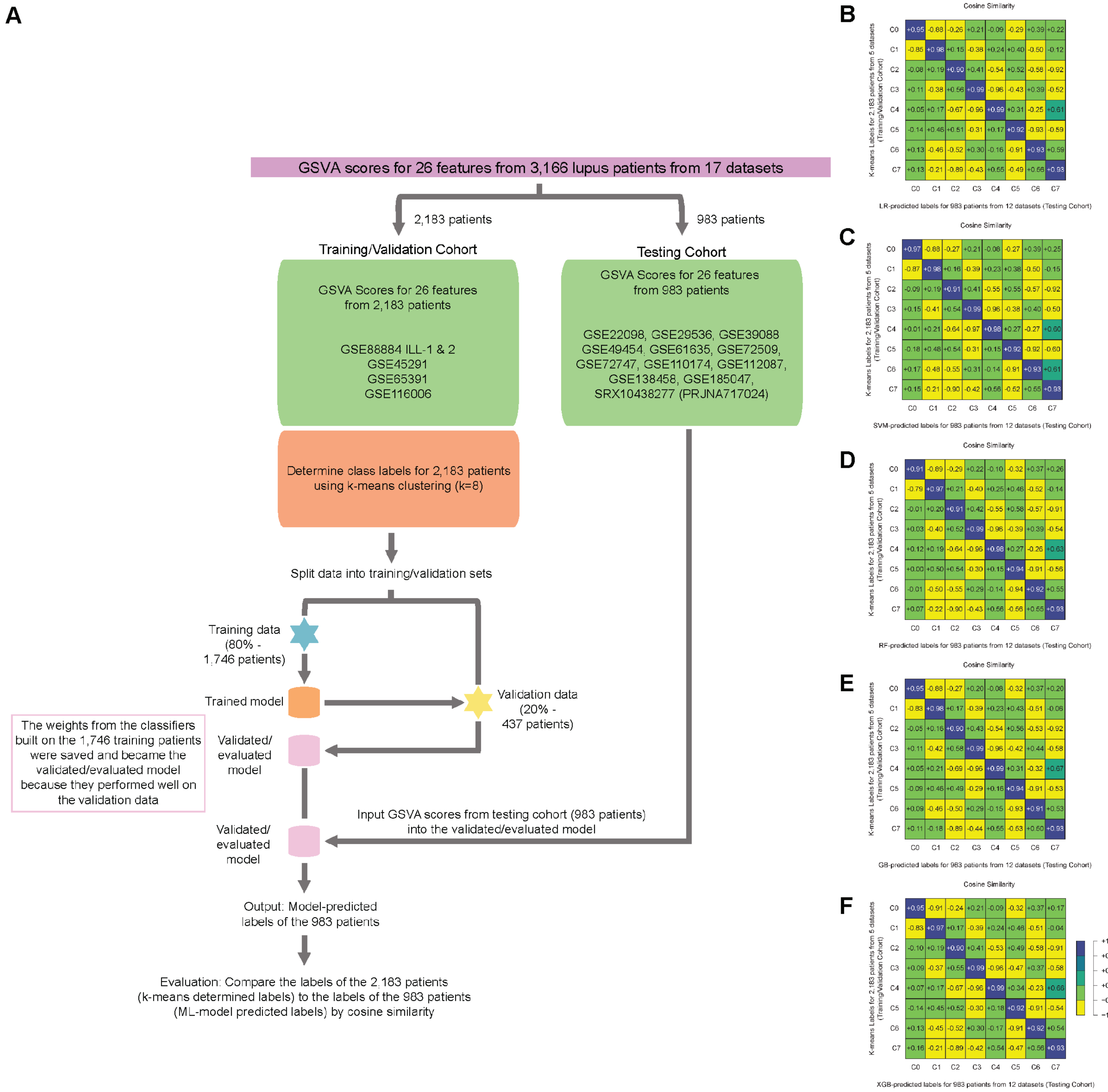

Figure S11. Unsupervised ML design to validate the classification of patients into final endotypes. As a third approach to confirm the validity of ML models shown in the main text, another ML design was carried out. (A) For development and validation of multiple ML classifiers, the 3,166 patients were split into a training/internal validation cohort (five datasets, n=2,183; training (n=1,746), internal validation (n=437)) and a testing cohort (12 datasets, n=983). The class labels of the 2,183 patients were generated using k-means clustering (k=8). The performance of ML classifiers built on the training data was evaluated using the validation samples, and the performance was satisfactory. Thus, the ML model weights from the training data were set as the final ML model weights. These ML models were then used to predict the labels of the 983 patients of the testing (external validation) cohort. After subsets were generated for the test set of 983 patients, cosine similarity was employed to compare the subsets generated from the class labels of training/internal validation cohort (generated by k-means clustering) and the test (external validation) cohort (generated by the ML models) using weights from (B) logistic regression, (C) support vector machine, (D) random forest, (E) gradient boosting, and (F) extreme gradient boosting (XGB). Flow diagram created in Adobe Illustrator. The cosine similarity plots were generated in R with the plot.matrix package and edited in Adobe Illustrator.

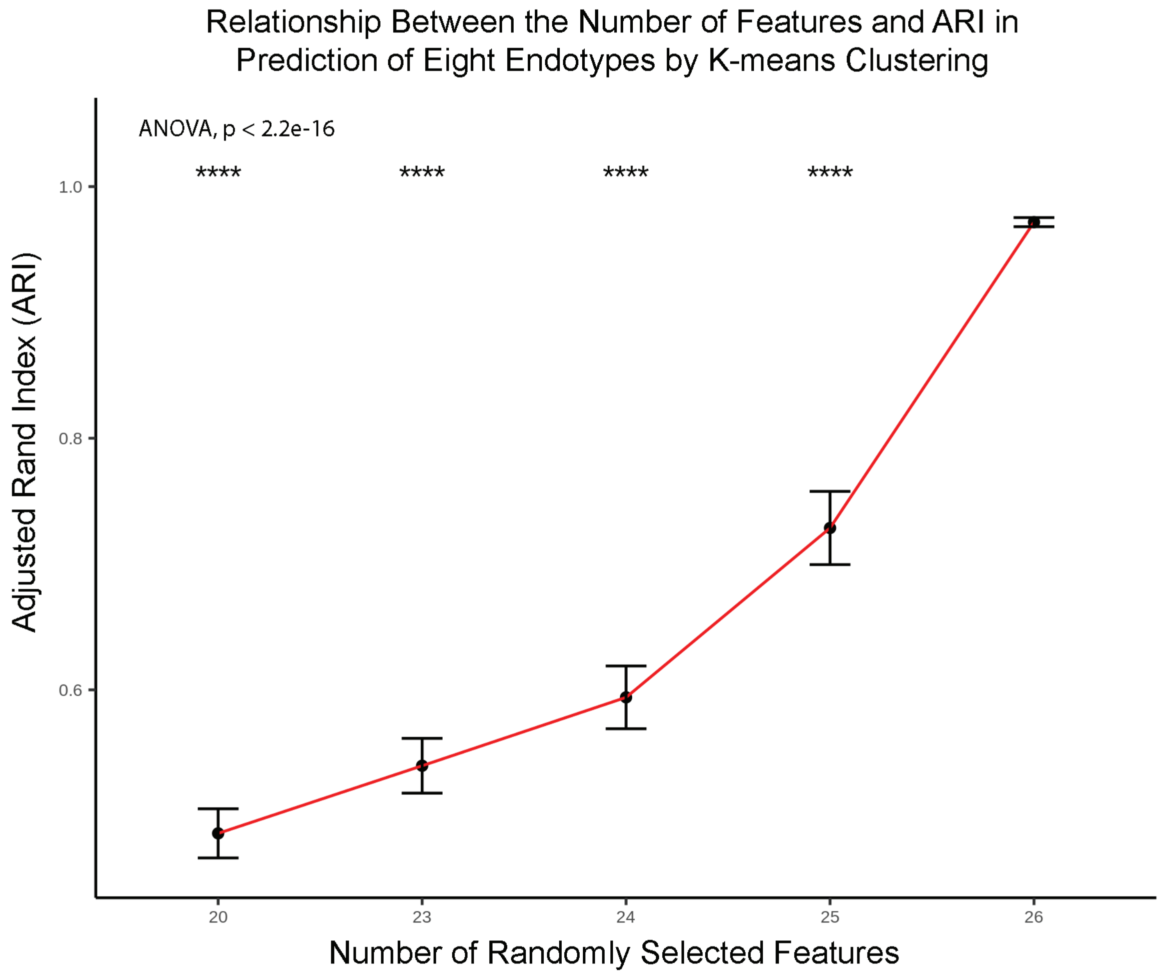

Figure S12. Optimal endotype identification requires all 26 features.
To determine the optimal number of features for subsetting, 0-6 features were randomly deleted from the initial 26 features over 100 iterations and the remaining 20-26 features used as input into k-means clustering of the 3,166 samples from 17 datasets. The composition of the resulting subsets was compared to the reference cluster (the original k-means result with all 26 features on the 3,166 samples) by adjusted rand index (ARI). ARI is plotted as mean±SD over 100 iterations. Significant differences in the mean ARI derived from each number of randomly selected features was calculated by ANOVA and t-test. Results of the t-test compared to the ARI derived using all 26 features are shown above each point. ARI was calculated in Python with scikit-learn and plots were generated in R with the ggplot package. *p<0.05; **p<0.01; ***p<0.001; ****p<0.0001.

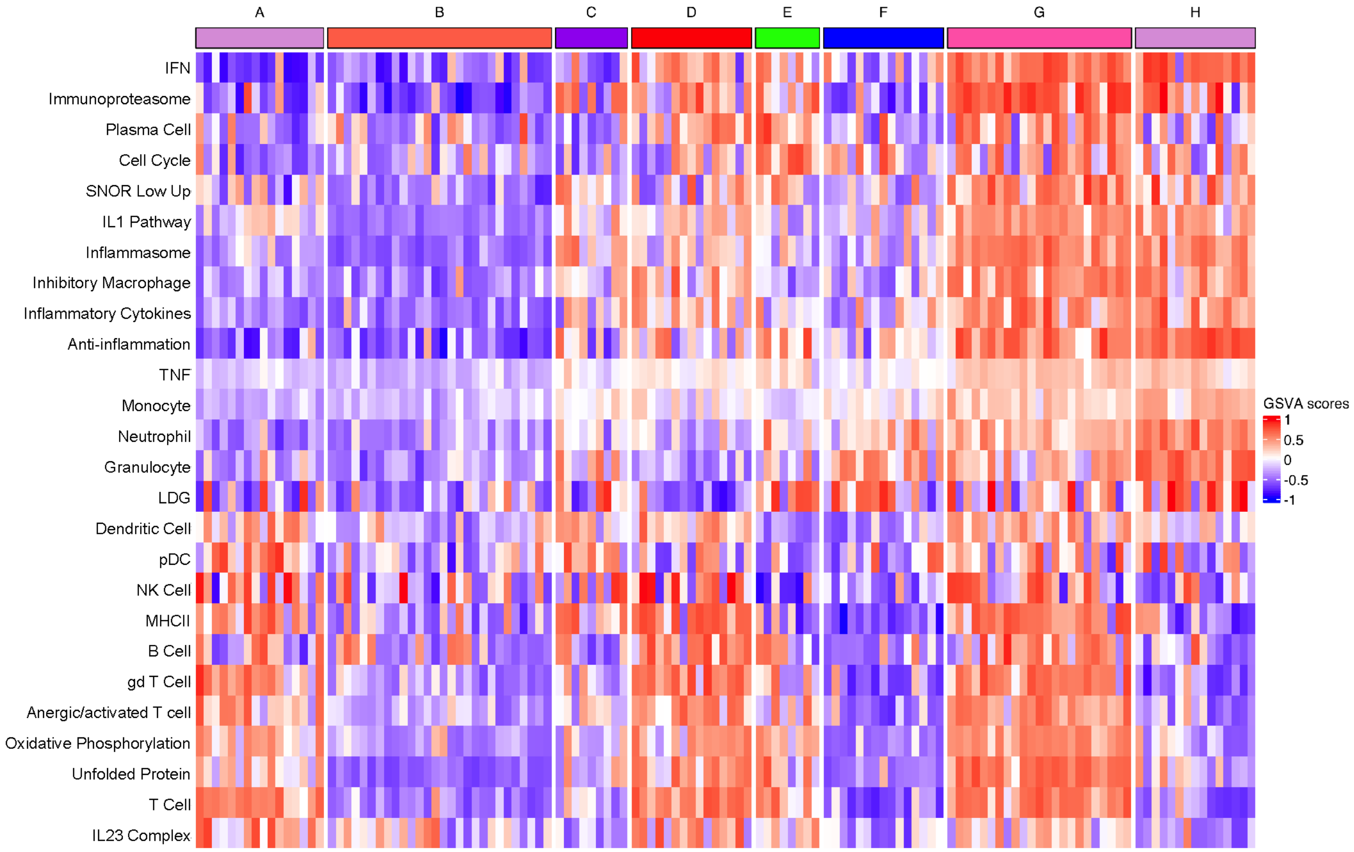

Figure S13. ML-Predicted endotypes in male SLE patients.
RF classification of 129 male SLE samples from GSE88884 ILL-1 & ILL-2 into the eight lupus endotypes. Model weights from the ML classifier built on training data (2,183 samples) were applied to GSVA scores for the 26 informative gene modules of the 129 samples to predict the lupus subsets. The heatmap was generated in R with the ComplexHeatmap package and ordered from “least abnormal” to “most abnormal” immunologic activity.

A Random forest classification of the eight endotypes of lupus

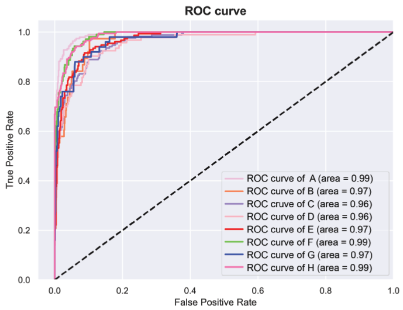

	Sensitivity	Specificity	Precision	Accuracy	f1-score
A	0.84	0.99	0.93	0.97	0.88
B	0.88	0.99	0.91	0.97	0.89
C	0.76	0.97	0.75	0.94	0.75
D	0.84	0.98	0.86	0.97	0.85
E	0.84	0.98	0.83	0.97	0.83
F	0.78	0.96	0.66	0.95	0.72
G	0.93	0.98	0.89	0.97	0.91
H	0.85	0.98	0.89	0.97	0.87

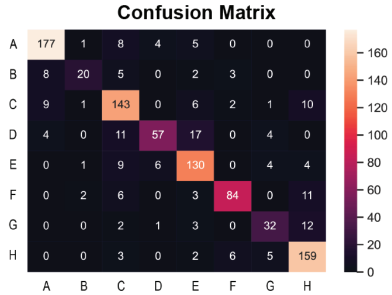

B Support vector machine classification of the eight endotypes of lupus

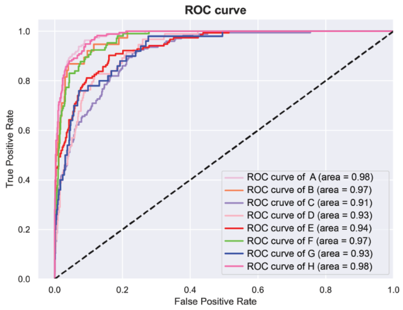

	Sensitivity	Specificity	Precision	Accuracy	f1-score
A	0.83	0.98	0.85	0.95	0.84
B	0.93	0.99	0.93	0.98	0.93
C	0.64	0.92	0.50	0.89	0.56
D	0.76	0.99	0.89	0.96	0.82
E	0.62	0.98	0.76	0.94	0.68
F	0.68	0.98	0.71	0.95	0.69
G	0.90	0.97	0.86	0.96	0.88
H	0.85	0.97	0.84	0.96	0.84

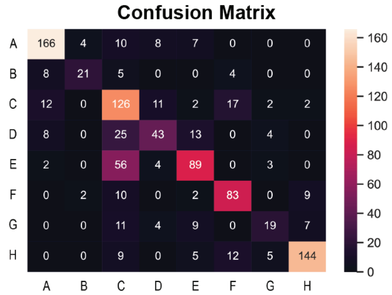

C Logistic regression classification of the eight endotypes of lupus

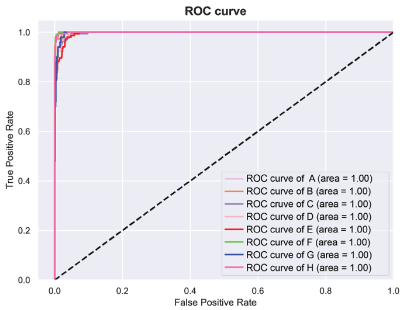

	Sensitivity	Specificity	Precision	Accuracy	f1-score
A	0.97	0.99	0.94	0.99	0.95
B	0.98	1.00	1.00	1.00	0.99
C	0.94	0.99	0.94	0.99	0.94
D	0.98	1.00	0.98	1.00	0.98
E	0.91	0.99	0.95	0.99	0.93
F	0.92	0.99	0.92	0.99	0.92
G	0.97	0.99	0.96	0.99	0.96
H	0.97	0.99	0.97	0.99	0.97

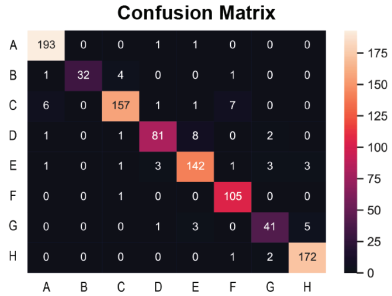

D Gradient boosting machines classification of the eight endotypes of lupus

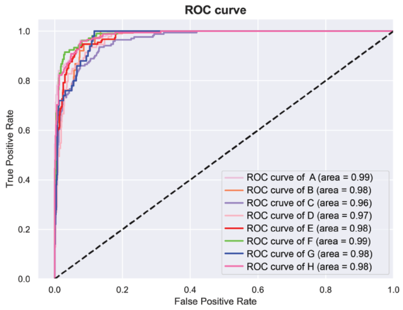

	Sensitivity	Specificity	Precision	Accuracy	f1-score
A	0.83	0.97	0.84	0.95	0.83
B	0.84	0.99	0.92	0.97	0.88
C	0.78	0.97	0.75	0.95	0.76
D	0.86	0.97	0.81	0.96	0.83
E	0.78	0.97	0.78	0.95	0.78
F	0.86	0.97	0.74	0.96	0.80
G	0.89	0.98	0.89	0.96	0.89
H	0.85	0.99	0.93	0.97	0.89

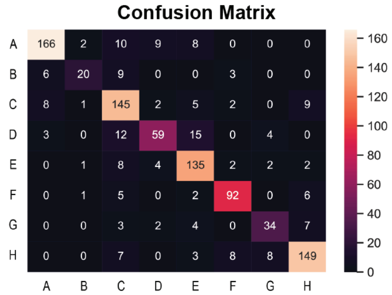

E Extreme gradient boosting classification of the eight endotypes of lupus

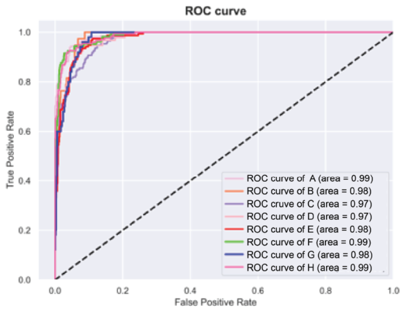

	Sensitivity	Specificity	Precision	Accuracy	f1-score
A	0.86	0.98	0.86	0.96	0.86
B	0.88	0.98	0.89	0.97	0.88
C	0.70	0.97	0.76	0.94	0.73
D	0.84	0.97	0.80	0.96	0.82
E	0.76	0.98	0.85	0.96	0.80
F	0.86	0.97	0.71	0.96	0.78
G	0.88	0.98	0.90	0.96	0.89
H	0.88	0.98	0.87	0.97	0.87

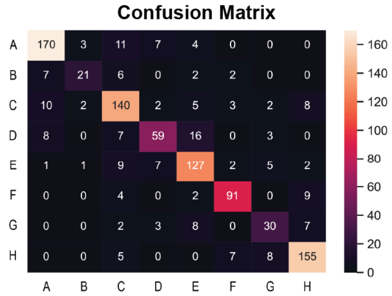

Figure S14. One-vs-rest multi-class classification of lupus endotype memberships.
One-vs-rest multi-class classification by ML analysis. Area under the ROC curve (AUC), performance metrics, and confusion matrices of each of five classifiers are summarized: (A) random forest, (B) support vector machine, (C) logistic regression, (D) gradient boosting, and (E) extreme gradient boosting (XGB). Each model was trained on 1,746 lupus samples, validated with 437 samples, and tested on the remaining 983 samples for a total n=3,166 from 17 datasets. Plots were created in Python with matplotlib.

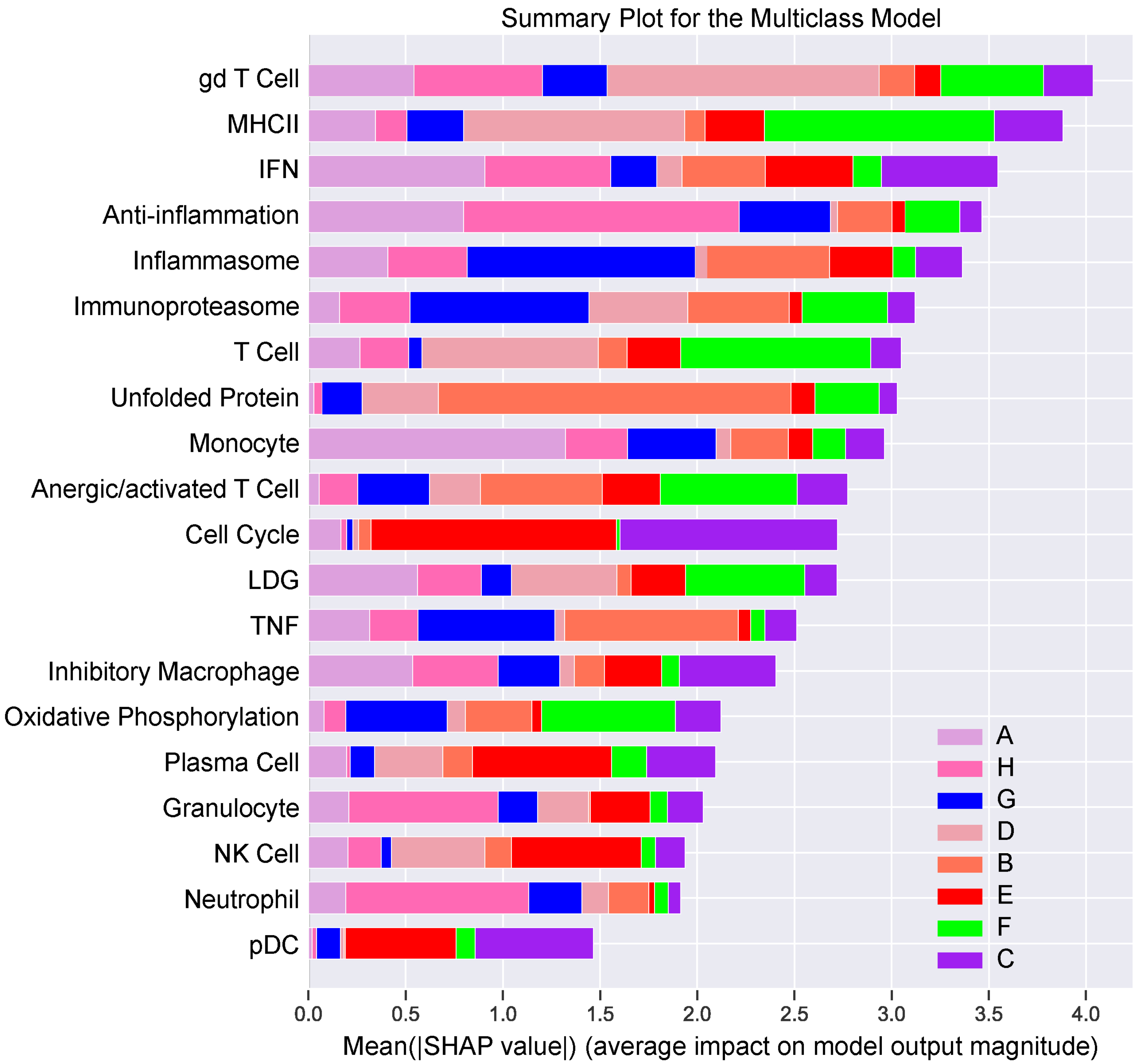

Figure S15. Summary Plot of SHAP values for XGB multi-class ML model.
Mean absolute SHAP values of the top 20 features of the XGB multi-class one-vs-rest ML model across patients in eight endotypes identified in 3,166 samples from 17 lupus whole blood datasets. A refers to the least perturbed endotype (Figure 3A) and H refers to the most perturbed endotype. The plot was generated in Python with the shap module.

A

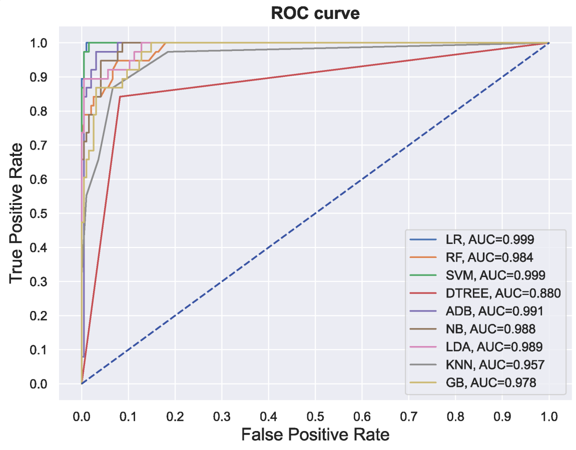

	Classifier	Sensitivity	Specificity	Cohen_Kappa_Score	Precision	f-1 score	Accuracy
0	LR	0.89	1.00	0.934329	1.00	0.941799	0.98
1	RF	0.79	0.98	0.817385	0.91	0.845765	0.95
2	SVM	0.87	0.99	0.901494	0.97	0.917283	0.97
3	DTREE	0.84	0.92	0.687248	0.67	0.745430	0.91
4	ADB	0.87	0.98	0.856987	0.89	0.879886	0.96
5	NB	0.74	0.99	0.793866	0.93	0.824192	0.95
6	LDA	0.79	0.99	0.847169	0.97	0.870795	0.96
7	KNN	0.66	0.96	0.664217	0.78	0.715000	0.91
8	GB	0.76	0.97	0.770152	0.85	0.802484	0.94

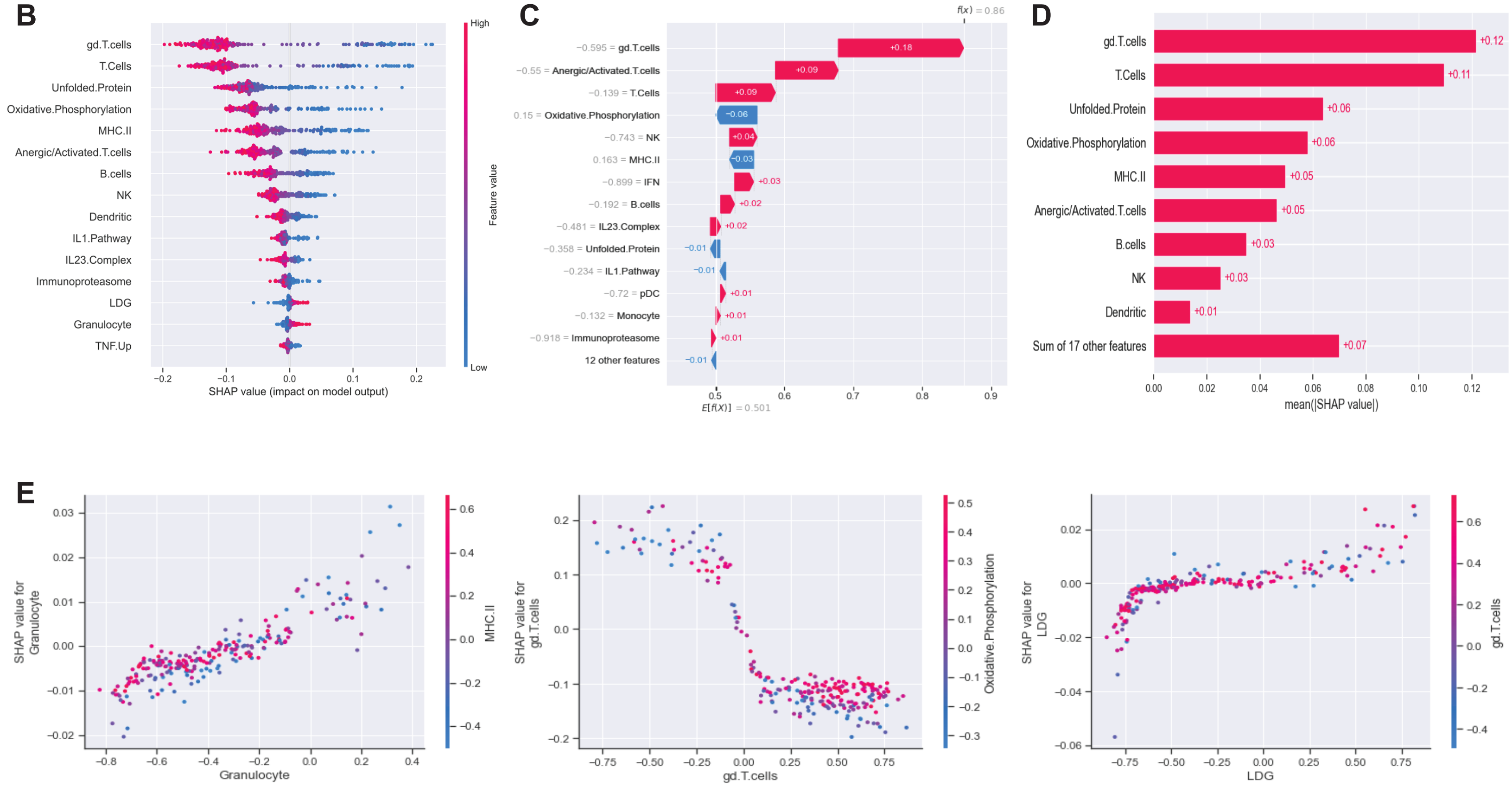

Figure S16. Distinguishment of endotype B from A.
Results of binary classification to distinguish endotype B (Figure 3A) from A using the GSVA enrichment scores of 26/32 molecular features present in all 17 datasets (Table S1). (A) ML performance metrics of nine classifiers. LR = logistic regression, RF = random forest, SVM = support vector machine, DTREE = decision trees, ADB = adaptive boosting, NB = naïve Bayes, LDA = linear discriminant analysis, KNN = k nearest neighbors, GB = gradient boosting. (B) RF SHAP summary plot of the top 15 important features ranked top to bottom. Feature value represents the GSVA enrichment score and each dot is a sample. (C) RF SHAP waterfall plot of an exemplary individual sample. Expected SHAP values are displayed on the bottom x-axis (calculated as the average SHAP value across all samples) and the actual SHAP value for that sample is displayed on the top x-axis. GSVA scores are displayed next to y-axis labels for the top 15 features. Values in the red and blue boxes in the plot enumerate how much a feature increases or decreases the final model outcome for the exemplary sample. (D) RF SHAP bar plot which enumerates the contribution of each feature across all samples. (E) RF SHAP dependence plots or scatter plots illustrating the impact of each feature toward the final model prediction and its interaction with a second feature for three exemplary features. The x-axis represents the GSVA score of the primary feature and the color represents the GSVA score of the secondary feature.

A

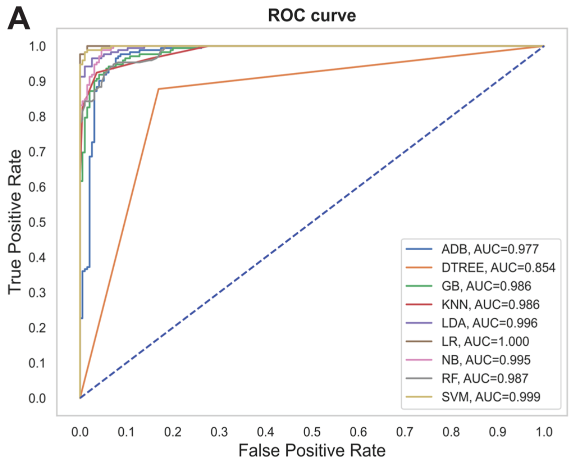

	Classifier	Sensitivity	Specificity	Cohen_Kappa_Score	Precision	f-1 score	Accuracy
0	ADB	0.97	0.92	0.885539	0.91	0.939043	0.94
1	DTREE	0.88	0.83	0.705772	0.82	0.848941	0.85
2	GB	0.90	0.97	0.868152	0.96	0.929032	0.93
3	KNN	0.92	0.96	0.890353	0.96	0.939574	0.95
4	LDA	0.94	0.98	0.928705	0.98	0.959583	0.96
5	LR	0.98	0.99	0.972635	0.99	0.984975	0.99
6	NB	0.99	0.94	0.923667	0.93	0.959063	0.96
7	RF	0.90	0.95	0.851824	0.94	0.919565	0.93
8	SVM	0.98	0.98	0.967174	0.98	0.980000	0.98

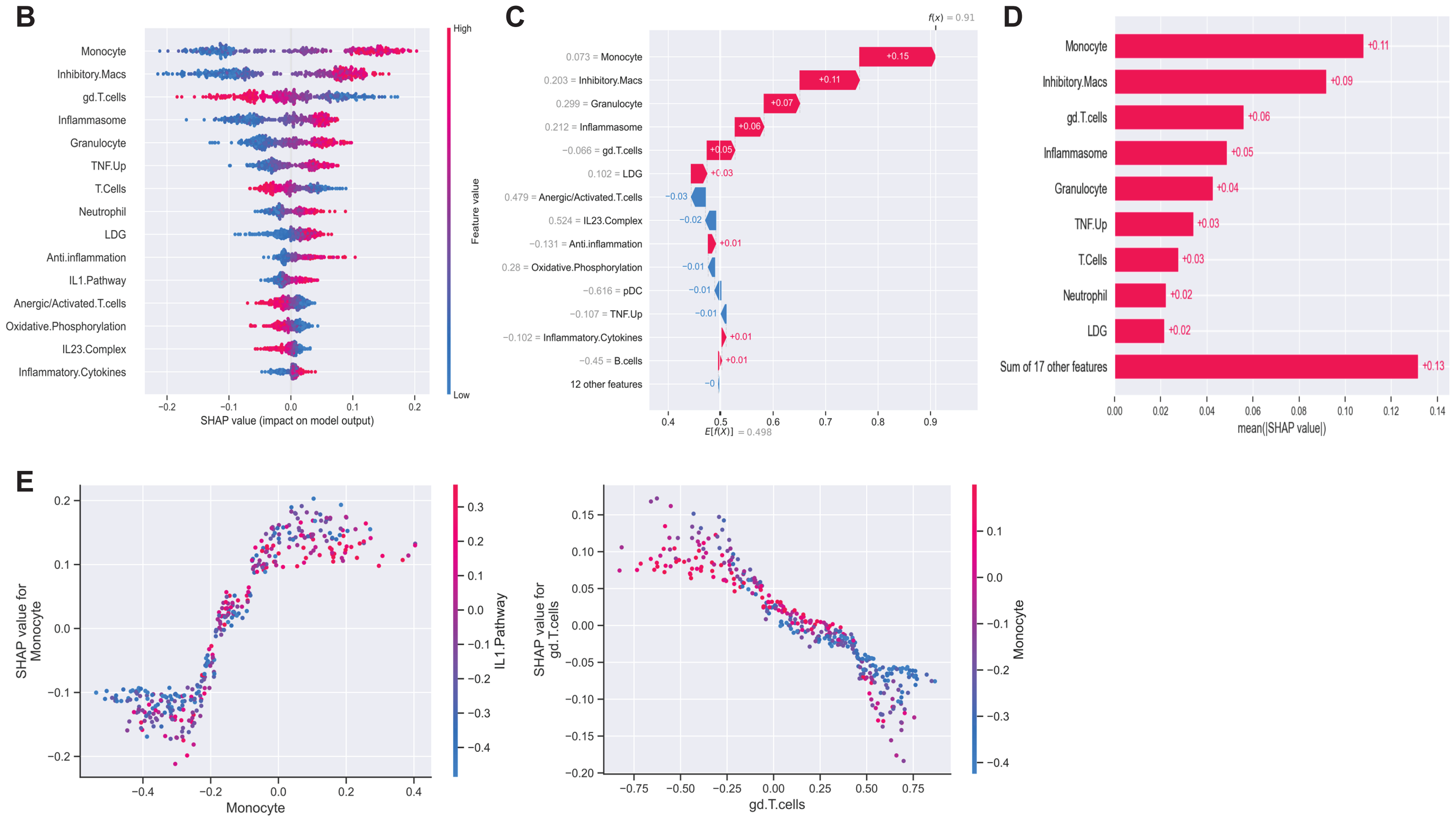

Figure S17. Distinguishment of endotype C from A.

Results of binary classification to distinguish endotype C (Figure 3A) from A using the GSVA enrichment scores of 26/32 molecular features present in all 17 datasets (Table S1). (A) ML performance metrics of nine classifiers. LR = logistic regression, RF = random forest, SVM = support vector machine, DTREE = decision trees, ADB = adaptive boosting, NB = naïve Bayes, LDA = linear discriminant analysis, KNN = k nearest neighbors, GB = gradient boosting. (B) RF SHAP summary plot of the top 15 important features ranked top to bottom. Feature value represents the GSVA enrichment score and each dot is a sample. (C) RF SHAP waterfall plot of an exemplary individual sample. Expected SHAP values are displayed on the bottom x-axis (calculated as the average SHAP value across all samples) and the actual SHAP value for that sample is displayed on the top x-axis. GSVA scores are displayed next to y-axis labels for the top 15 features. Values in the red and blue boxes in the plot enumerate how much a feature increases or decreases the final model outcome for the exemplary sample. (D) RF SHAP bar plot which enumerates the contribution of each feature across all samples. (E) RF SHAP dependence plots or scatter plots illustrating the impact of each feature toward the final model prediction and its interaction with a second feature for two exemplary features. The x-axis represents the GSVA score of the primary feature and the color represents the GSVA score of the secondary feature.

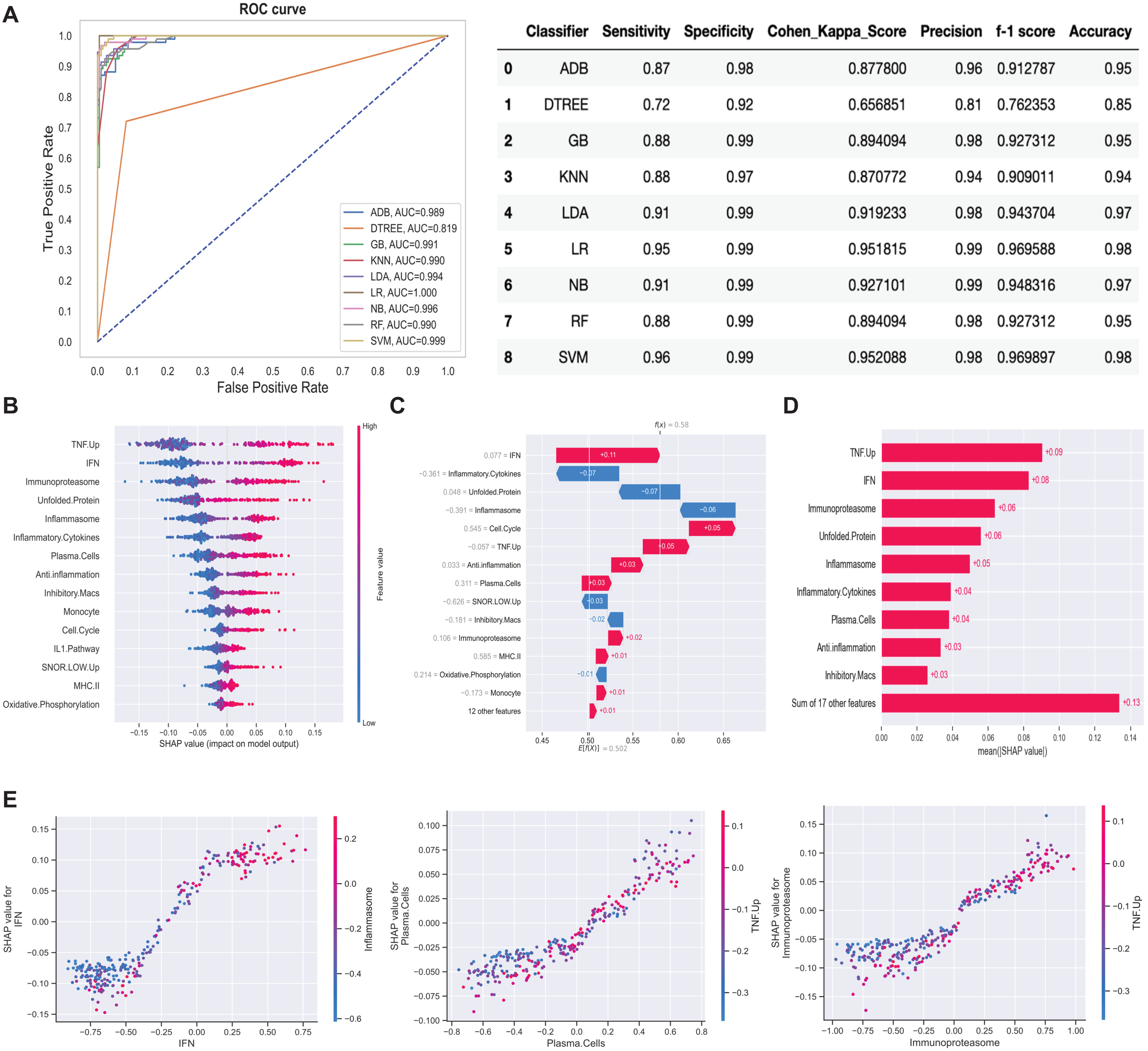

	Classifier	Sensitivity	Specificity	Cohen_Kappa_Score	Precision	f-1 score	Accuracy
0	ADB	0.87	0.98	0.877800	0.96	0.912787	0.95
1	DTREE	0.72	0.92	0.656851	0.81	0.762353	0.85
2	GB	0.88	0.99	0.894094	0.98	0.927312	0.95
3	KNN	0.88	0.97	0.870772	0.94	0.909011	0.94
4	LDA	0.91	0.99	0.919233	0.98	0.943704	0.97
5	LR	0.95	0.99	0.951815	0.99	0.969588	0.98
6	NB	0.91	0.99	0.927101	0.99	0.948316	0.97
7	RF	0.88	0.99	0.894094	0.98	0.927312	0.95
8	SVM	0.96	0.99	0.952088	0.98	0.969897	0.98

Figure S18. Distinguishment of endotype D from A.

Results of binary classification to distinguish endotype D (Figure 3A) from A using the GSVA enrichment scores of 26/32 molecular features present in all 17 datasets (Table S1). (A) ML performance metrics of nine classifiers. LR = logistic regression, RF = random forest, SVM = support vector machine, DTREE = decision trees, ADB = adaptive boosting, NB = naïve Bayes, LDA = linear discriminant analysis, KNN = k nearest neighbors, GB = gradient boosting. (B) RF SHAP summary plot of the top 15 important features ranked top to bottom. Feature value represents the GSVA enrichment score and each dot is a sample. (C) RF SHAP waterfall plot of an exemplary individual sample. Expected SHAP values are displayed on the bottom x-axis (calculated as the average SHAP value across all samples) and the actual SHAP value for that sample is displayed on the top x-axis. GSVA scores are displayed next to y-axis labels for the top 15 features. Values in the red and blue boxes in the plot enumerate how much a feature increases or decreases the final model outcome for the exemplary sample. (D) RF SHAP bar plot which enumerates the contribution of each feature across all samples. (E) RF SHAP dependence plots or scatter plots illustrating the impact of each feature toward the final model prediction and its interaction with a second feature for three exemplary features. The x-axis represents the GSVA score of the primary feature and the color represents the GSVA score of the secondary feature.

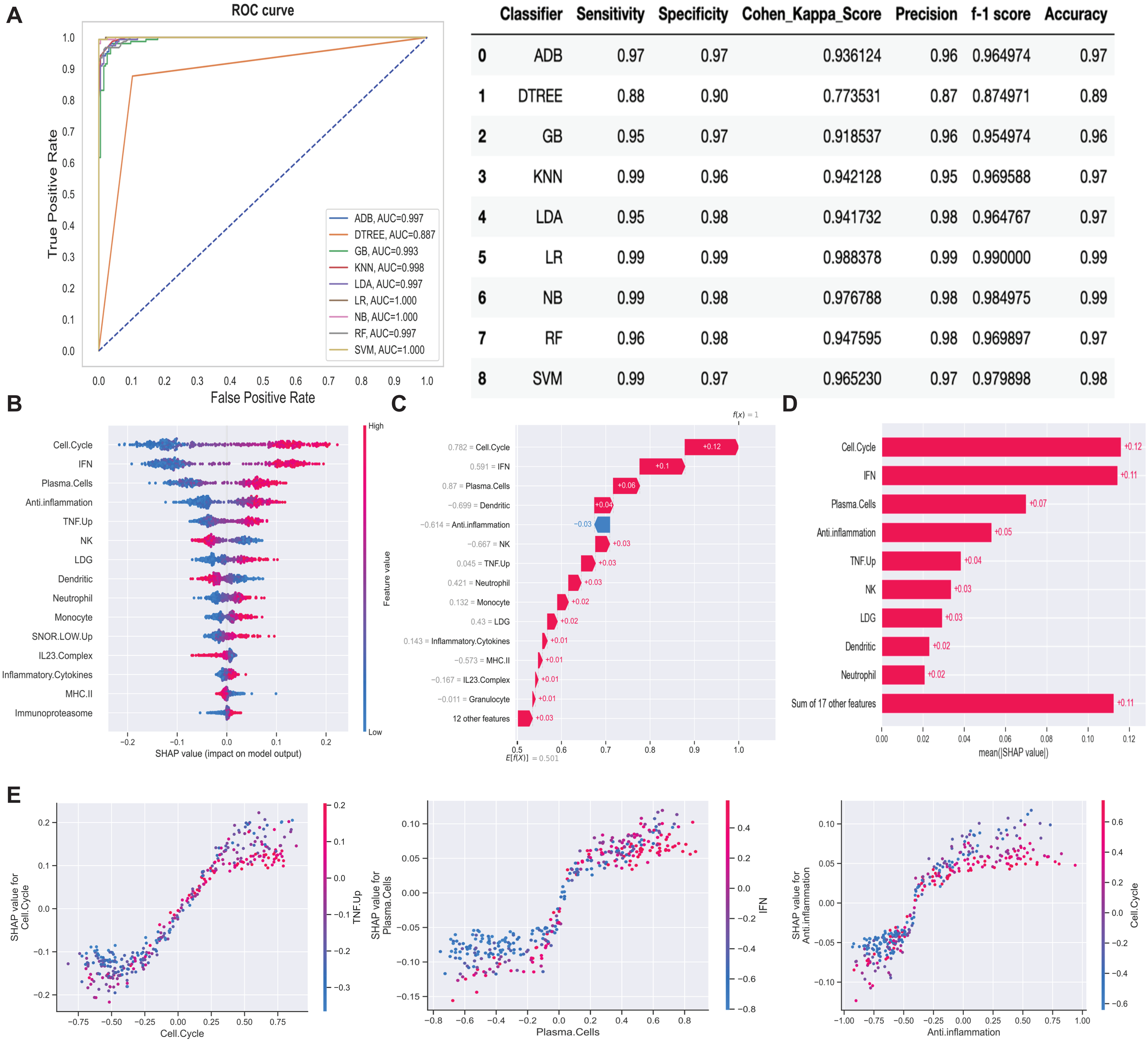

	Classifier	Sensitivity	Specificity	Cohen_Kappa_Score	Precision	f-1 score	Accuracy
0	ADB	0.97	0.97	0.936124	0.96	0.964974	0.97
1	DTREE	0.88	0.90	0.773531	0.87	0.874971	0.89
2	GB	0.95	0.97	0.918537	0.96	0.954974	0.96
3	KNN	0.99	0.96	0.942128	0.95	0.969588	0.97
4	LDA	0.95	0.98	0.941732	0.98	0.964767	0.97
5	LR	0.99	0.99	0.988378	0.99	0.990000	0.99
6	NB	0.99	0.98	0.976788	0.98	0.984975	0.99
7	RF	0.96	0.98	0.947595	0.98	0.969897	0.97
8	SVM	0.99	0.97	0.965230	0.97	0.979898	0.98

Figure S19. Distinguishment of endotype E from A.

Results of binary classification to distinguish endotype E (Figure 3A) from A using the GSVA enrichment scores of 26/32 molecular features present in all 17 datasets (Table S1). (A) ML performance metrics of nine classifiers. LR = logistic regression, RF = random forest, SVM = support vector machine, DTREE = decision trees, ADB = adaptive boosting, NB = naïve Bayes, LDA = linear discriminant analysis, KNN = k nearest neighbors, GB = gradient boosting. (B) RF SHAP summary plot of the top 15 important features ranked top to bottom. Feature value represents the GSVA enrichment score and each dot is a sample. (C) RF SHAP waterfall plot of an exemplary individual sample. Expected SHAP values are displayed on the bottom x-axis (calculated as the average SHAP value across all samples) and the actual SHAP value for that sample is displayed on the top x-axis. GSVA scores are displayed next to y-axis labels for the top 15 features. Values in the red and blue boxes in the plot enumerate how much a feature increases or decreases the final model outcome for the exemplary sample. (D) RF SHAP bar plot which enumerates the contribution of each feature across all samples. (E) RF SHAP dependence plots or scatter plots illustrating the impact of each feature toward the final model prediction and its interaction with a second feature for three exemplary features. The x-axis represents the GSVA score of the primary feature and the color represents the GSVA score of the secondary feature.

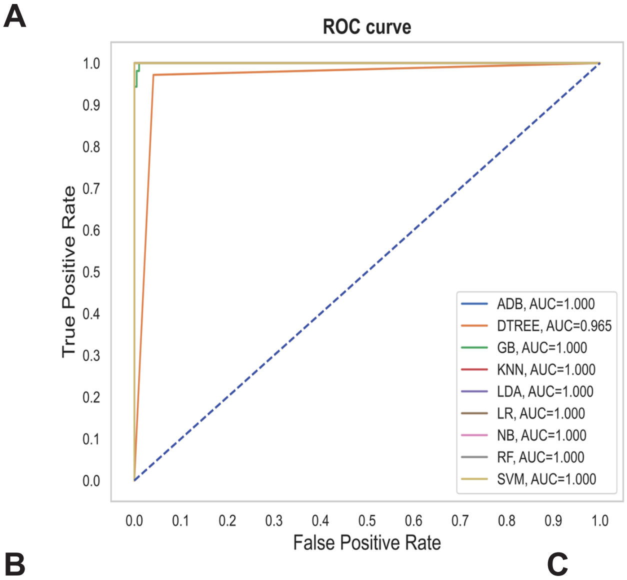

	Classifier	Sensitivity	Specificity	Cohen_Kappa_Score	Precision	f-1 score	Accuracy
0	ADB	1.00	1.00	1.000000	1.00	1.000000	1.00
1	DTREE	0.97	0.96	0.920761	0.93	0.949579	0.96
2	GB	0.99	0.99	0.978204	0.98	0.984975	0.99
3	KNN	1.00	1.00	1.000000	1.00	1.000000	1.00
4	LDA	1.00	1.00	1.000000	1.00	1.000000	1.00
5	LR	1.00	1.00	1.000000	1.00	1.000000	1.00
6	NB	1.00	1.00	1.000000	1.00	1.000000	1.00
7	RF	0.99	1.00	0.992703	1.00	0.994975	1.00
8	SVM	1.00	1.00	1.000000	1.00	1.000000	1.00

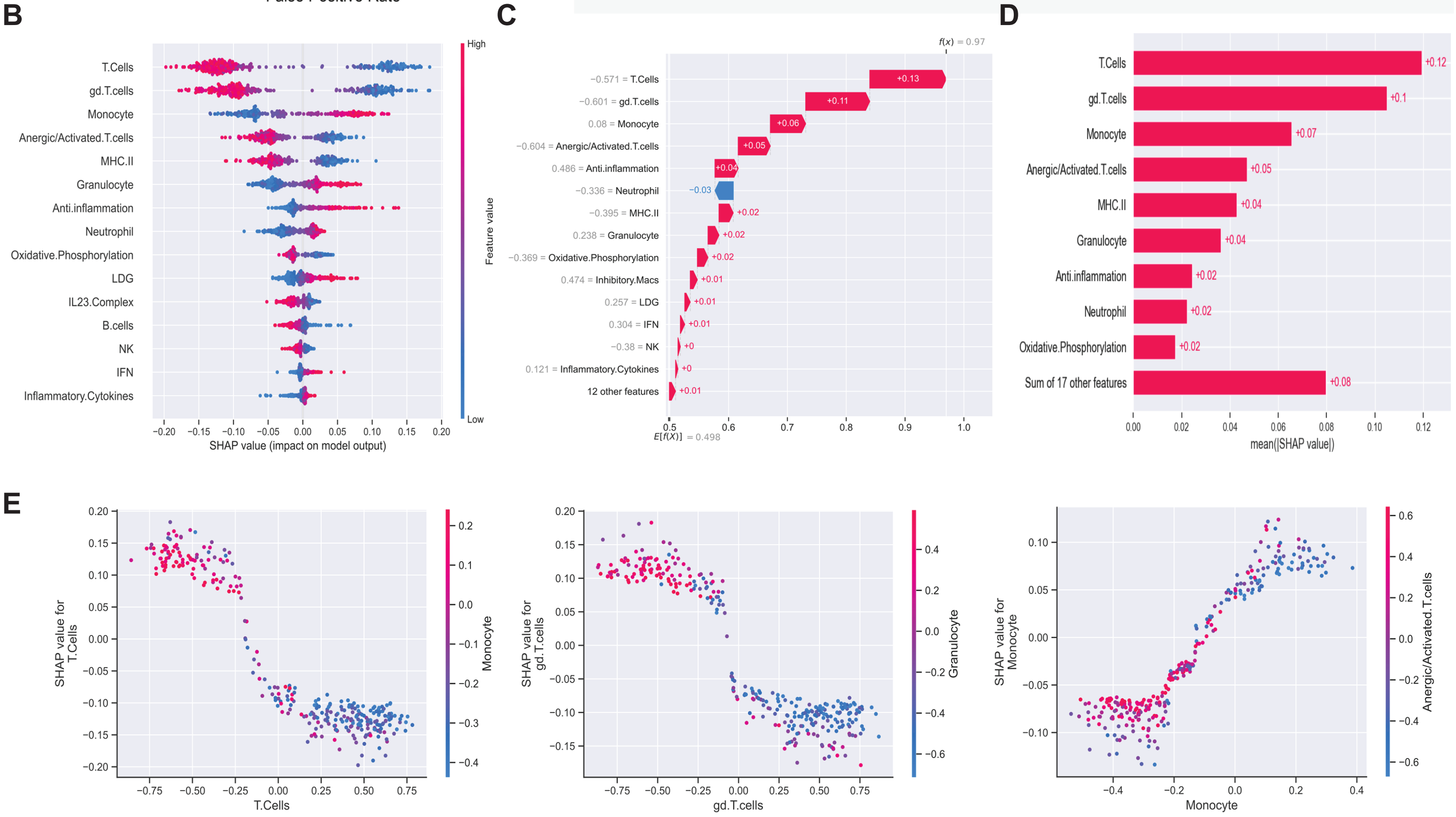

Figure S20. Distinguishment of endotype F from A.

Results of binary classification to distinguish endotype F (Figure 3A) from A using the GSVA enrichment scores of 26/32 molecular features present in all 17 datasets (Table S1). (A) ML performance metrics of nine classifiers. LR = logistic regression, RF = random forest, SVM = support vector machine, DTREE = decision trees, ADB = adaptive boosting, NB = naïve Bayes, LDA = linear discriminant analysis, KNN = k nearest neighbors, GB = gradient boosting. (B) RF SHAP summary plot of the top 15 important features ranked top to bottom. Feature value represents the GSVA enrichment score and each dot is a sample. (C) RF SHAP waterfall plot of an exemplary individual sample. Expected SHAP values are displayed on the bottom x-axis (calculated as the average SHAP value across all samples) and the actual SHAP value for that sample is displayed on the top x-axis. GSVA scores are displayed next to y-axis labels for the top 15 features. Values in the red and blue boxes in the plot enumerate how much a feature increases or decreases the final model outcome for the exemplary sample. (D) RF SHAP bar plot which enumerates the contribution of each feature across all samples. (E) RF SHAP dependence plots or scatter plots illustrating the impact of each feature toward the final model prediction and its interaction with a second feature for three exemplary features. The x-axis represents the GSVA score of the primary feature and the color represents the GSVA score of the secondary feature.

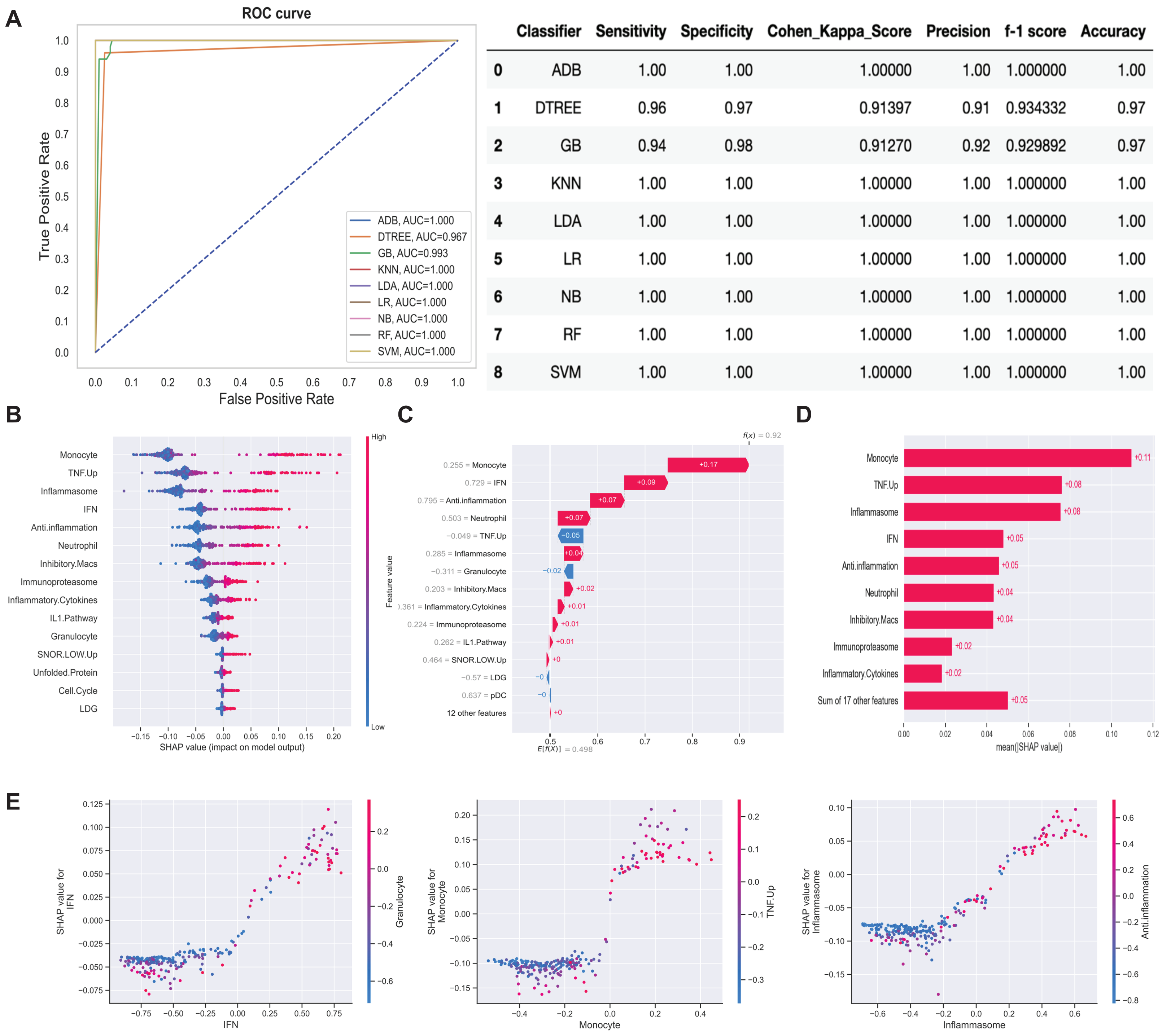

	Classifier	Sensitivity	Specificity	Cohen_Kappa_Score	Precision	f-1 score	Accuracy
0	ADB	1.00	1.00	1.00000	1.00	1.000000	1.00
1	DTREE	0.96	0.97	0.91397	0.91	0.934332	0.97
2	GB	0.94	0.98	0.91270	0.92	0.929892	0.97
3	KNN	1.00	1.00	1.00000	1.00	1.000000	1.00
4	LDA	1.00	1.00	1.00000	1.00	1.000000	1.00
5	LR	1.00	1.00	1.00000	1.00	1.000000	1.00
6	NB	1.00	1.00	1.00000	1.00	1.000000	1.00
7	RF	1.00	1.00	1.00000	1.00	1.000000	1.00
8	SVM	1.00	1.00	1.00000	1.00	1.000000	1.00

Figure S21. Distinguishment of endotype G from A.
Results of binary classification to distinguish endotype G (Figure 3A) from A using the GSVA enrichment scores of 26/32 molecular features present in all 17 datasets (Table S1). (A) ML performance metrics of nine classifiers. LR = logistic regression, RF = random forest, SVM = support vector machine, DTREE = decision trees, ADB = adaptive boosting, NB = naïve Bayes, LDA = linear discriminant analysis, KNN = k nearest neighbors, GB = gradient boosting. (B) RF SHAP summary plot of the top 15 important features ranked top to bottom. Feature value represents the GSVA enrichment score and each dot is a sample. (C) RF SHAP waterfall plot of an exemplary individual sample. Expected SHAP values are displayed on the bottom x-axis (calculated as the average SHAP value across all samples) and the actual SHAP value for that sample is displayed on the top x-axis. GSVA scores are displayed next to y-axis labels for the top 15 features. Values in the red and blue boxes in the plot enumerate how much a feature increases or decreases the final model outcome for the exemplary sample. (D) RF SHAP bar plot which enumerates the contribution of each feature across all samples. (E) RF SHAP dependence plots or scatter plots illustrating the impact of each feature toward the final model prediction and its interaction with a second feature for three exemplary features. The x-axis represents the GSVA score of the primary feature and the color represents the GSVA score of the secondary feature.

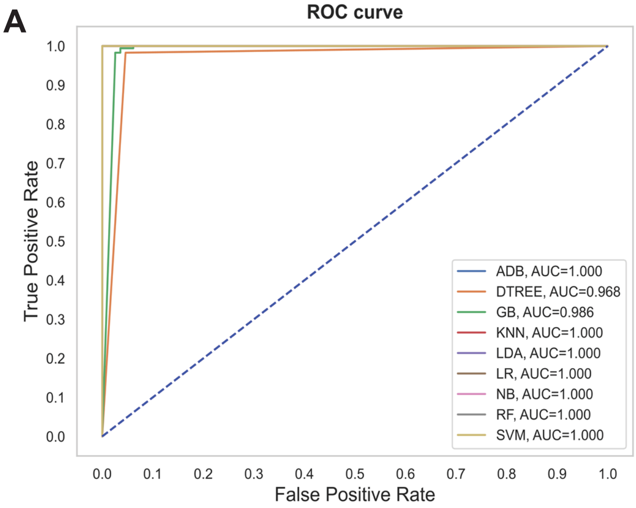

	Classifier	Sensitivity	Specificity	Cohen_Kappa_Score	Precision	f-1 score	Accuracy
0	ADB	1.00	1.00	1.000000	1.00	1.000000	1.00
1	DTREE	0.98	0.95	0.935059	0.95	0.964767	0.97
2	GB	0.99	0.95	0.940523	0.95	0.969588	0.97
3	KNN	1.00	1.00	1.000000	1.00	1.000000	1.00
4	LDA	1.00	1.00	1.000000	1.00	1.000000	1.00
5	LR	1.00	1.00	1.000000	1.00	1.000000	1.00
6	NB	1.00	1.00	1.000000	1.00	1.000000	1.00
7	RF	1.00	1.00	1.000000	1.00	1.000000	1.00
8	SVM	1.00	1.00	1.000000	1.00	1.000000	1.00

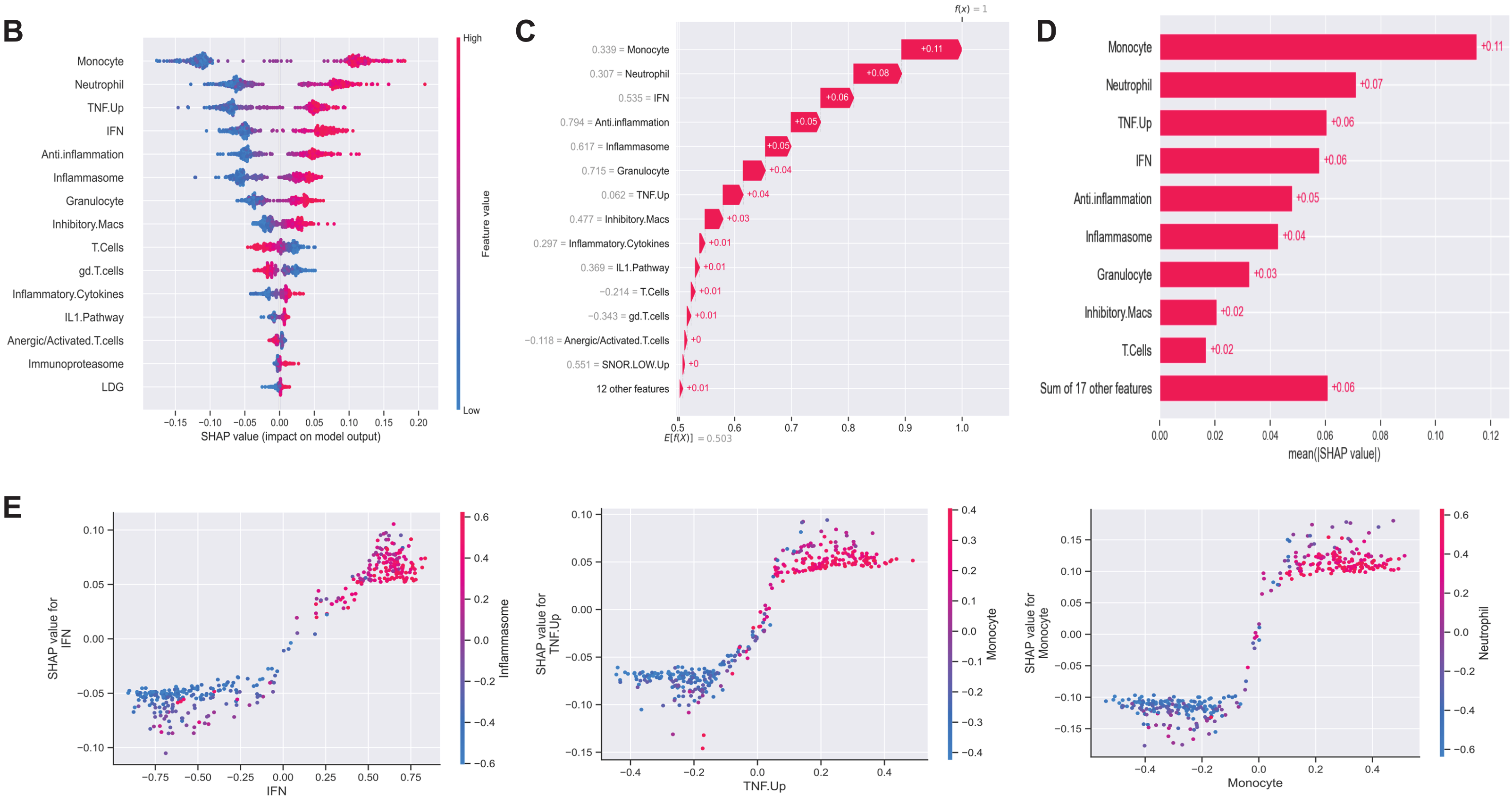

Figure S22. Distinguishment of endotype H from A.
Results of binary classification to distinguish endotype H (Figure 3A) from A using the GSVA enrichment scores of 26/32 molecular features present in all 17 datasets (Table S1). (A) ML performance metrics of nine classifiers. LR = logistic regression, RF = random forest, SVM = support vector machine, DTREE = decision trees, ADB = adaptive boosting, NB = naïve Bayes, LDA = linear discriminant analysis, KNN = k nearest neighbors, GB = gradient boosting. (B) RF SHAP summary plot of the top 15 important features ranked top to bottom. Feature value represents the GSVA enrichment score and each dot is a sample. (C) RF SHAP waterfall plot of an exemplary individual sample. Expected SHAP values are displayed on the bottom x-axis (calculated as the average SHAP value across all samples) and the actual SHAP value for that sample is displayed on the top x-axis. GSVA scores are displayed next to y-axis labels for the top 15 features. Values in the red and blue boxes in the plot enumerate how much a feature increases or decreases the final model outcome for the exemplary sample. (D) RF SHAP bar plot which enumerates the contribution of each feature across all samples. (E) RF SHAP dependence plots or scatter plots illustrating the impact of each feature toward the final model prediction and its interaction with a second feature for three exemplary features. The x-axis represents the GSVA score of the primary feature and the color represents the GSVA score of the secondary feature.

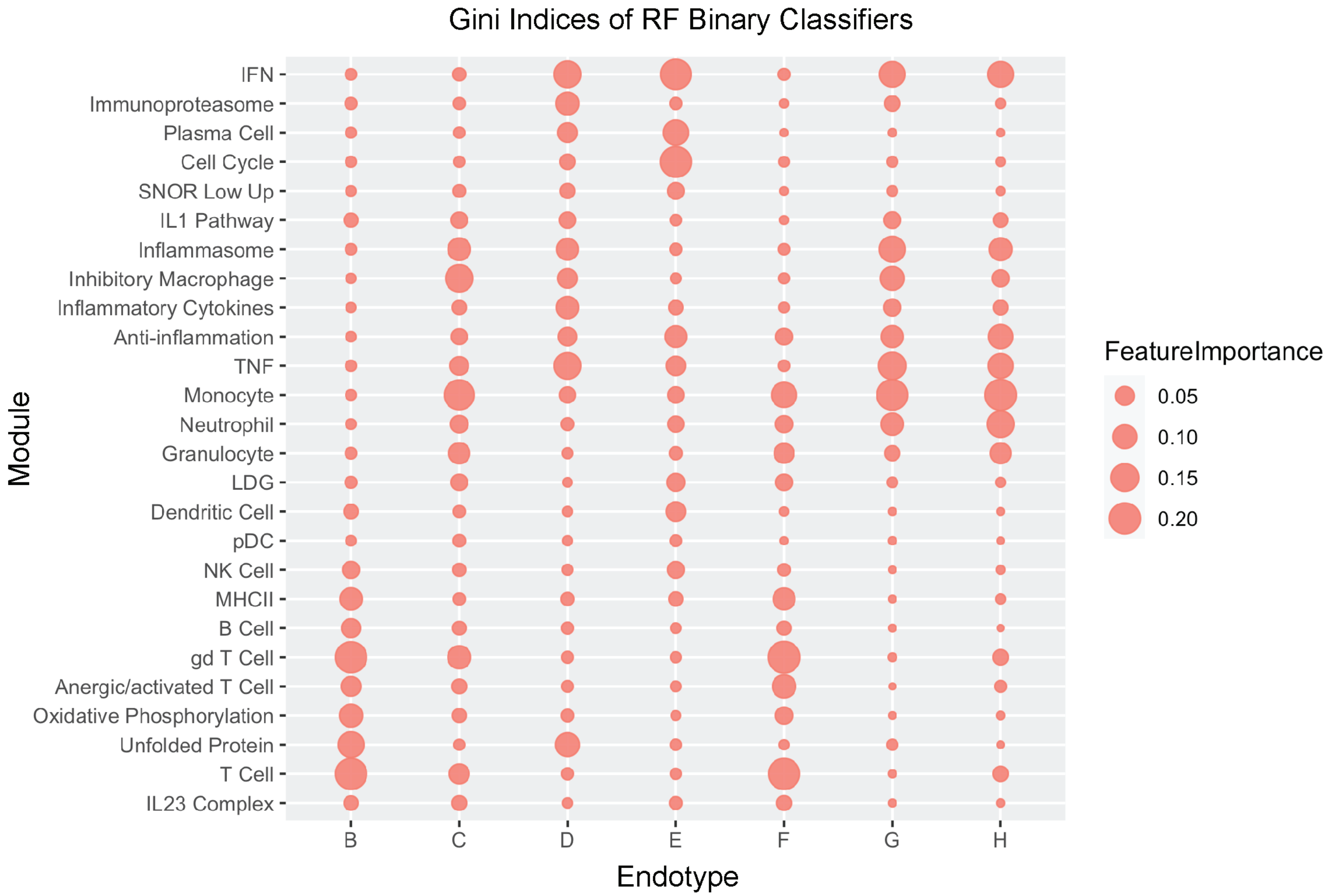

Figure S23. Gini index analysis reveals features most distinctive of transcriptional perturbations in the seven abnormal lupus endotypes.
Gini index analysis of the seven binary RF classifiers distinguishing the seven out of eight most transcriptionally abnormal lupus endotypes (B-H) from the eighth least abnormal endotype (A) reveals the features most contributory to the ML model's classification capacity. The size of the data points enumerates the Gini index value of each feature listed on the y-axis. Bubble plot rendered in R with the ggplot2 package.

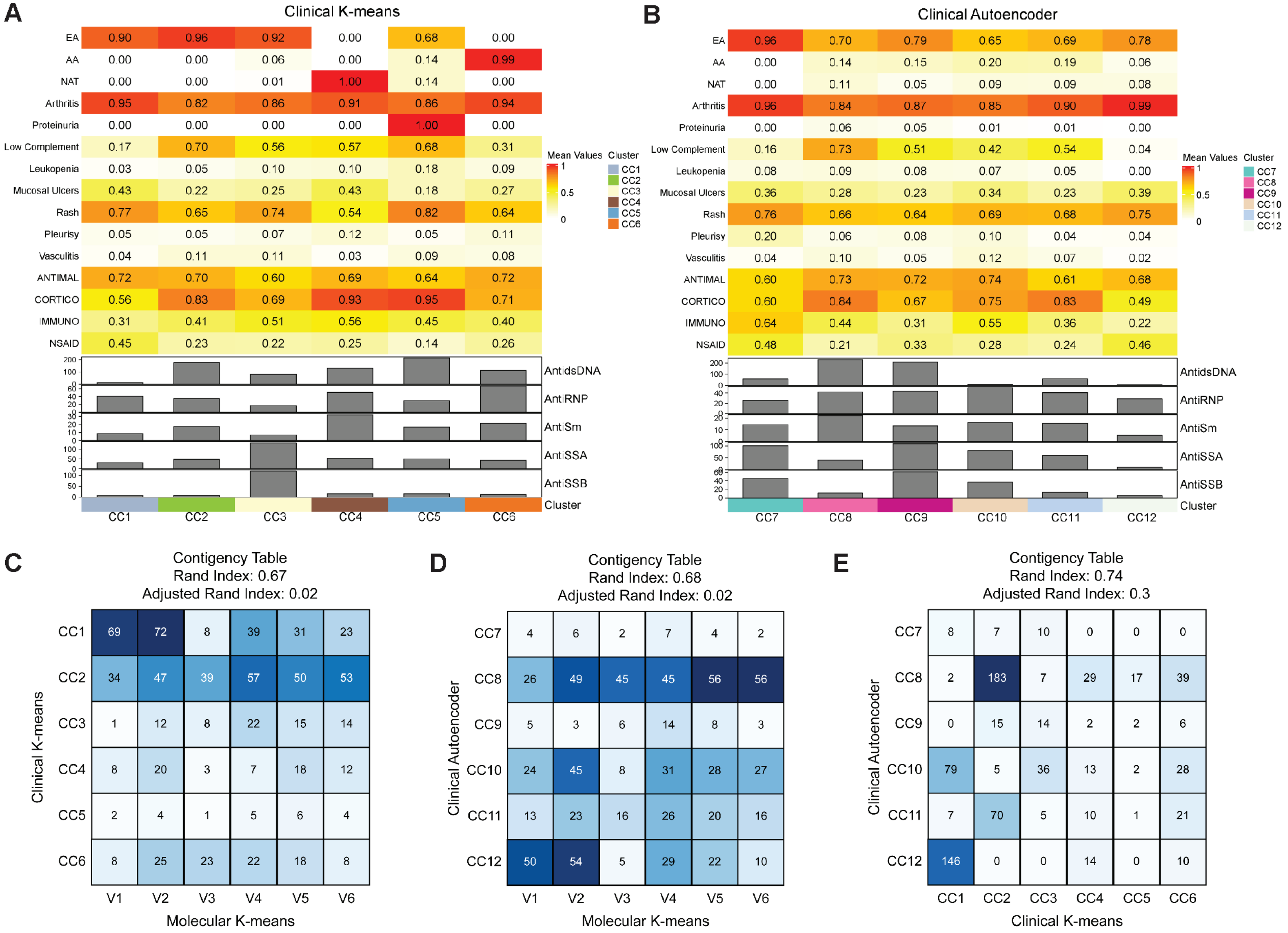

Figure S24. Lupus subsets derived from clinical features.
Clustering based on clinical metadata of 807 active lupus samples from GSE88884 ILL-2 by (A) k-means or (B) GMVAE. (C-E) Rand indices comparing the patient clusters derived using clinical data and/or molecular features by the k-means and GMVAE algorithms. The heatmap in (C) compares the clinical k-means endotypes and molecular endotypes, (D) compares molecular endotypes and clinical GMVAE endotypes, and (E) compares clinical k-means endotypes and clinical GMVAE endotypes. Heatmaps in (A-B) were generated in R with the ComplexHeatmap package. Categorical variables in (A-B) including ancestry, medication, and components of SLEDAI, were binarized. Heatmaps in (C-E) were visualized with the Seaborn package in Python and edited in Adobe Illustrator. Rand indices were calculated in Python with the scikit-learn package. EA = European Ancestry, AA = African Ancestry, NAT = Native American (Hispanic) Ancestry, ANTIMAL = antimalarials, CORTICO = corticosteroids, IMMUNO = immunosuppressants.

A Random forest classification of the molecular endotypes of GSE88884 ILL-2

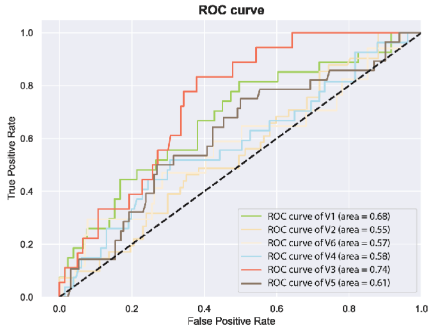

	Sensitivity	Specificity	Precision	Accuracy	f1-score
V1	0.37	0.93	0.50	0.85	0.43
V2	0.41	0.74	0.30	0.67	0.35
V6	0.20	0.86	0.21	0.75	0.20
V4	0.34	0.87	0.43	0.75	0.38
V3	0.30	0.94	0.30	0.89	0.30
V5	0.19	0.82	0.17	0.71	0.18

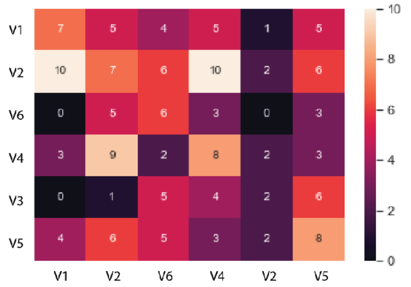

B Support vector machine classification of the molecular endotypes of GSE88884 ILL-2

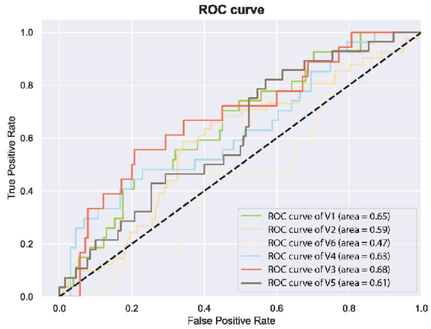

	Sensitivity	Specificity	Precision	Accuracy	f1-score
V1	0.21	0.95	0.44	0.84	0.28
V2	0.41	0.60	0.22	0.56	0.29
V6	0.00	1.00	NaN	0.84	NaN
V4	0.07	0.90	0.17	0.71	0.10
V3	0.50	0.86	0.24	0.83	0.32
V5	0.29	0.74	0.18	0.67	0.22

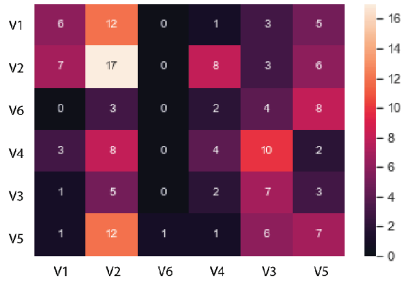

C Logistic regression classification of the molecular endotypes of GSE88884 ILL-2

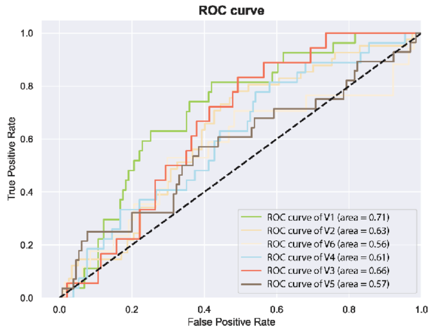

	Sensitivity	Specificity	Precision	Accuracy	f1-score
V1	0.26	0.94	0.45	0.84	0.33
V2	0.30	0.70	0.21	0.61	0.25
V6	0.20	0.83	0.18	0.73	0.19
V4	0.34	0.89	0.48	0.76	0.40
V3	0.50	0.95	0.45	0.91	0.47
V5	0.29	0.84	0.26	0.75	0.27

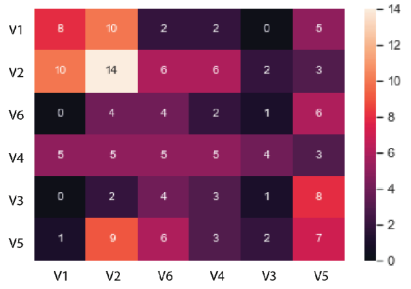

D Gradient boosting machines classification of the molecular endotypes of GSE88884 ILL-2

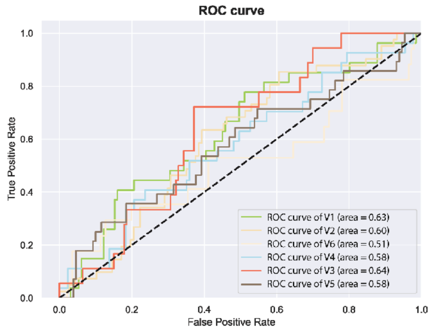

	Sensitivity	Specificity	Precision	Accuracy	f1-score
V1	0.42	0.94	0.57	0.87	0.48
V2	0.30	0.81	0.30	0.70	0.30
V6	0.40	0.76	0.24	0.71	0.30
V4	0.17	0.82	0.23	0.67	0.20
V3	0.50	0.96	0.50	0.92	0.50
V5	0.10	0.83	0.10	0.71	0.10

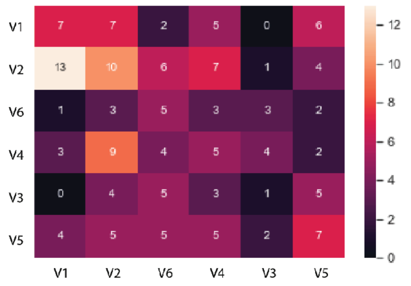

Figure S25. Machine learning prediction of molecular endotype memberships using clinical metadata as features.

One-vs.-one ML classifiers predicting the molecular endotype memberships of GSE88884 ILL-2 using baseline clinical metadata as features rather than baseline gene expression. (A) RF, (B) SVM, (C) LR, and (D) GB classifier ROC curves, performance metrics, and classification schema. Clinical metadata used to determine subsets by either k-means clustering or GMVAE were used as input features: patient ancestry, presence of arthritis, proteinuria, low complement, leukopenia, mucosal ulcers, rash, pleurisy, vasculitis, use of antimalarials, use of corticosteroids, use of immunosuppressants, use of non-steroidal anti-inflammatory drugs, and anti-dsDNA, anti-RNP, anti-Sm, anti-SSA, and anti-SSB titers. Figure components were generated in Python with matplotlib and edited in Adobe Illustrator.

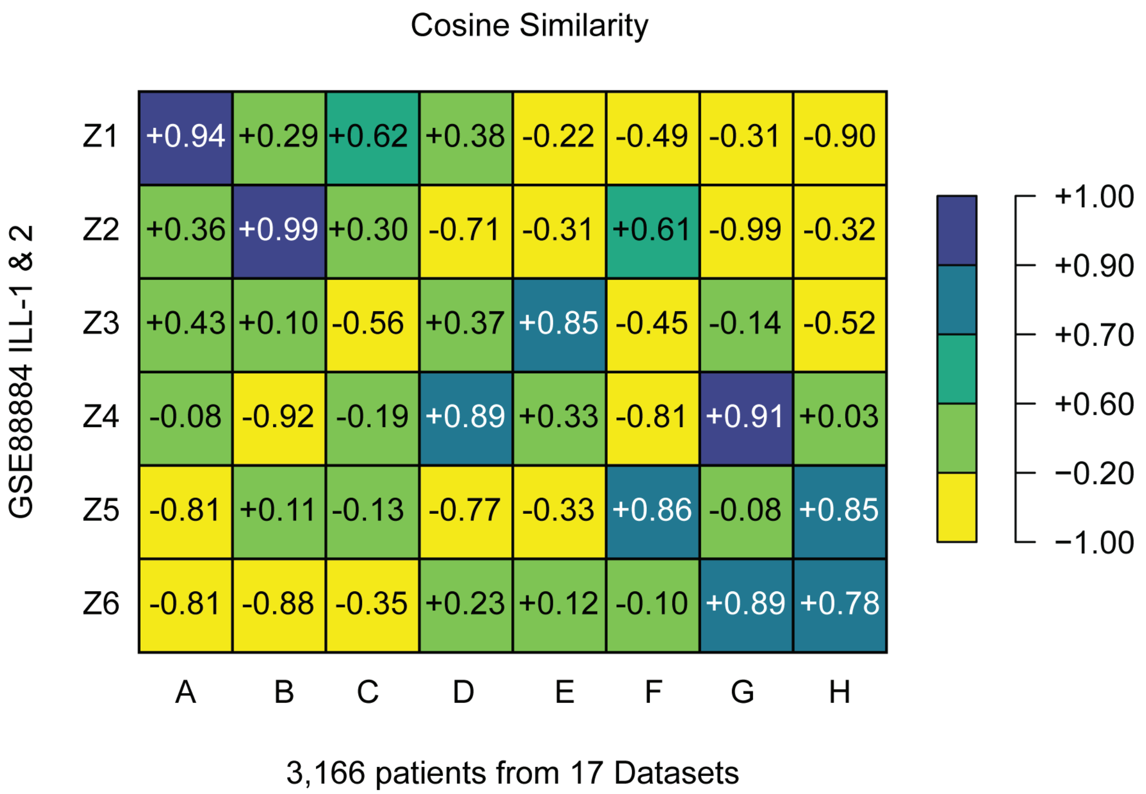

Figure S26. Endotype reassignment in GSE88884.
Cosine similarity of the identified endotypes in each GSE88884 ILL-1 & ILL-2 combined (n=1,620) and the entire lupus patient cohort (n=3,166). The cosine similarity plot was generated in R with the plot.matrix package and edited in Adobe Illustrator.

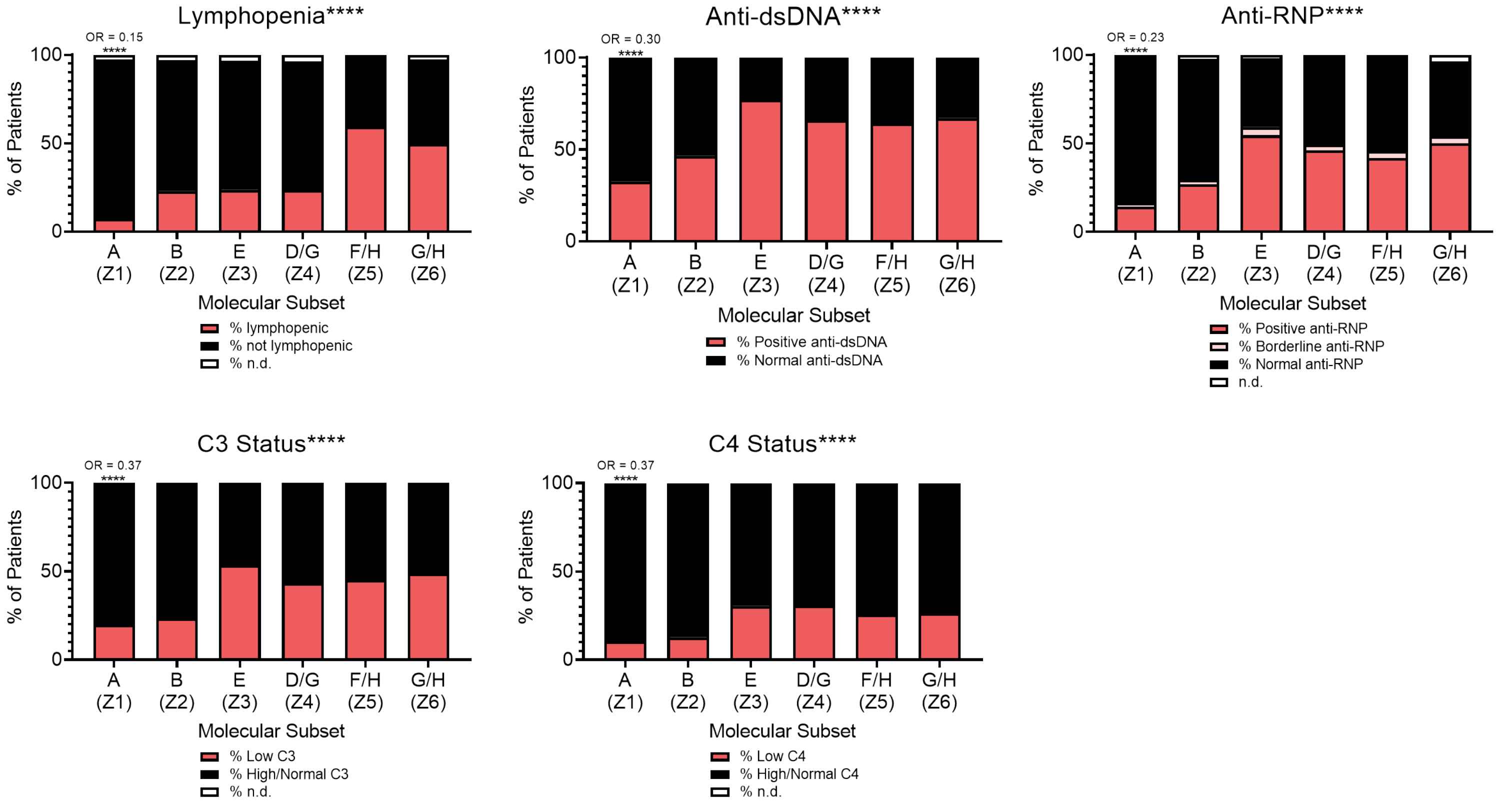

Figure S27. Clinical characterization of SLE endotypes

Clinical metadata of immunological/inflammatory disease indicators was summarized for each cluster from GSE88884 (ILL-1 & ILL-2) using baseline values. Clusters (labels on x-axes) were relabeled as one of the eight endotypes using cosine similarity. Lymphopenia was defined as less than 1 billion lymphocytes per liter. Significant associations between categorical variables and endotypes (denoted with asterisks in titles) were identified using Chi-square Test of Independence. Odds ratios of endotype A (Z1) having a positive value for the clinical trait of interest as compared to the other cohorts combined are displayed above the respective bar with significance indicated by asterisks. Missing data (n.d.) were excluded from analyses. Graphs were created in GraphPad Prism v 9.4.0 (673). n.d. = no data. *p<0.05; **p<0.01; ***p<0.001; ****p<0.0001.

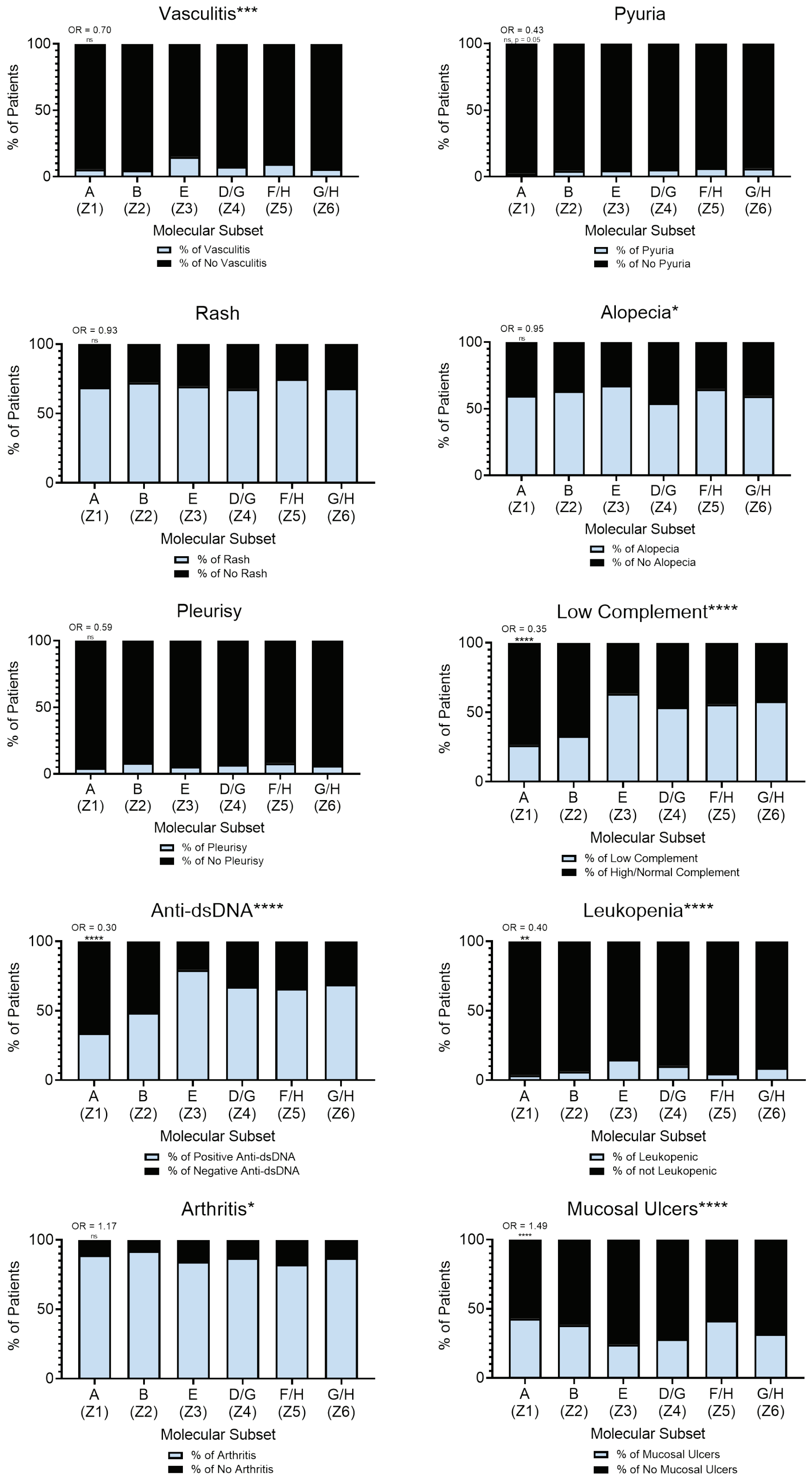

Figure S28. SLEDAI manifestations among six adult lupus endotypes in GSE88884.
K-means clustering of GSVA scores of the 32 features in 1,620 adult lupus patients from GSE88884 (ILL-1 & ILL-2) yielded six clusters using baseline gene expression. Clinical metadata were summarized for each cluster using baseline values of manifestations defined by SLEDAI. Clusters (labels on x-axes) were relabeled as one of the eight endotypes using cosine similarity. Significant associations between categorical variables and endotypes (denoted with asterisks in titles) were identified using Chi-square Test of Independence. Odds ratios of A (Z1) having a positive value for the clinical trait of interest are displayed above the A (Z1) bar with significance indicated by asterisks. Graphs were created in GraphPad Prism v 9.4.0 (673). *p<0.05; **p<0.01; ***p<0.001; ****p<0.0001.

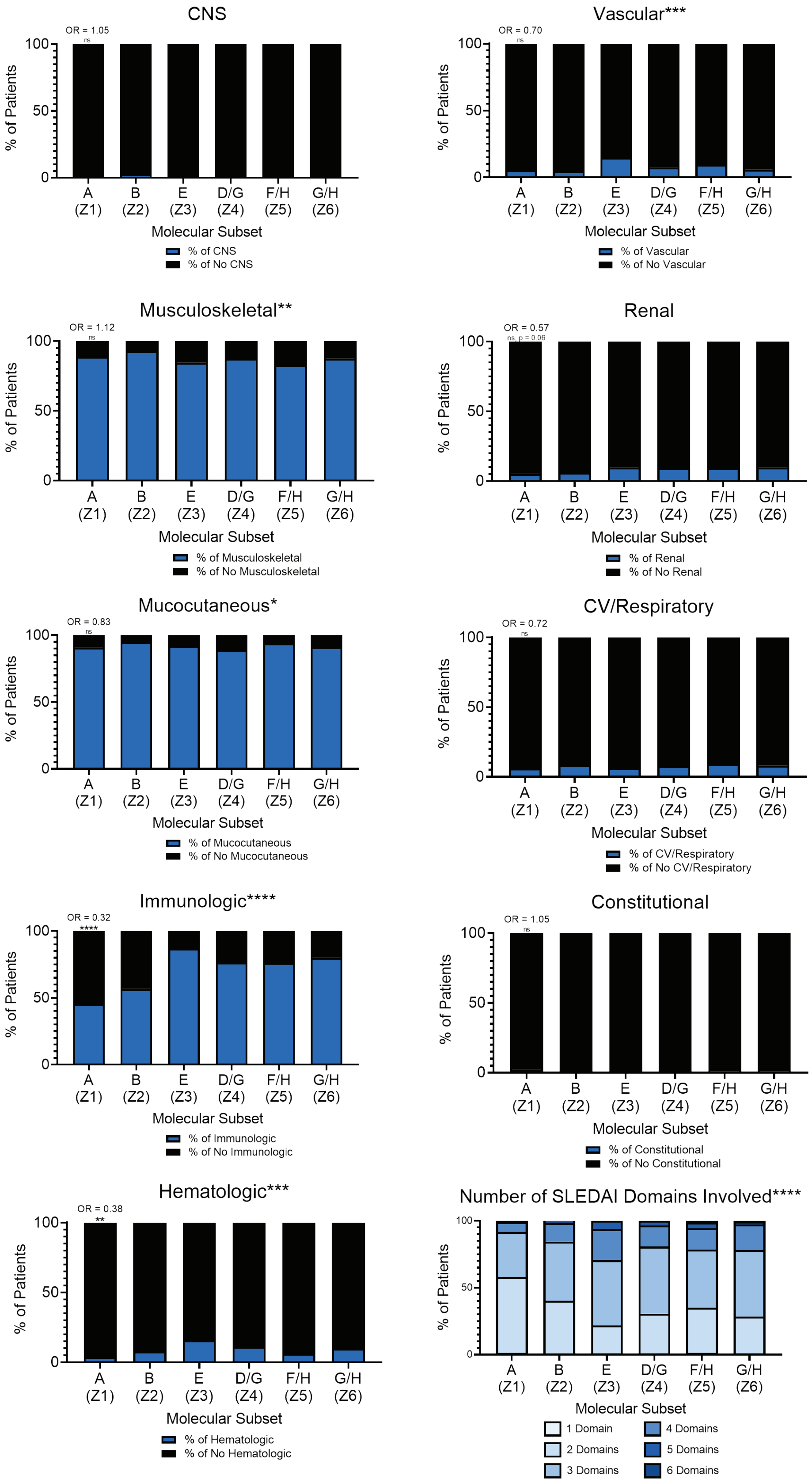

Figure S29. Organ system involvement among six adult lupus endotypes in GSE88884.
K-means clustering of GSVA scores of the 32 features in 1,620 adult lupus patients from GSE88884 (ILL-1 & ILL-2) yielded six clusters using baseline gene expression. Clinical metadata were summarized for each cluster using baseline values of organ system involvement defined by SLEDAI. Clusters (labels on x-axes) were relabeled as one of the eight endotypes using cosine similarity. Significant associations between categorical variables and endotypes (denoted with asterisks in titles) were identified using Chi-square Test of Independence. Odds ratios of A (Z1) having a positive value for the clinical trait of interest are displayed above the A (Z1) bar with significance indicated by asterisks. Graphs were created in GraphPad Prism v 9.4.0 (673). *p<0.05; **p<0.01; ***p<0.001; ****p<0.0001.

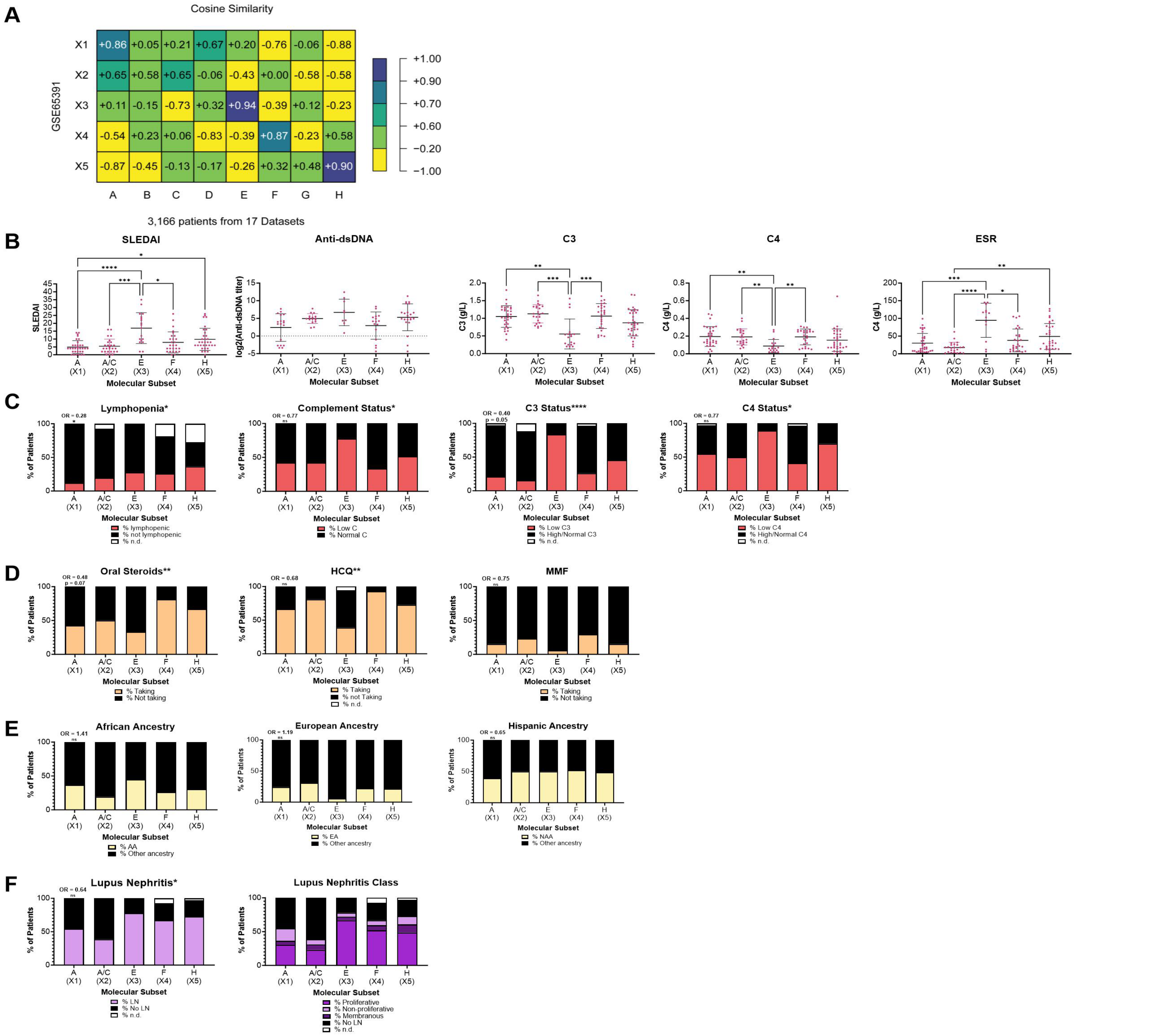

Figure S30. Clinical characteristics of five pediatric lupus endotypes.
(A) Cosine similarity comparing the eight SLE endotypes to the subsets in GSE65391. Quantitative and categorical clinical metadata were summarized for each endotype identified in GSE65391 using baseline values. Metadata was categorized by (B) quantitative immunologic/inflammatory and systemic disease indicators, (C) qualitative immunologic/inflammatory and systemic disease indicators, (D) medication use, (E) patient ancestry, and (F) kidney disease activity. Clusters in (B-F) were relabeled as one of the eight endotypes using cosine similarity. X2 was labeled as A/C, although it did not quite meet the cosine similarity cutoff of > 0.7. Scatterplots in (A) display the mean±SD for each endotype; statistical differences were found with Dunn’s multiple comparisons test. Lymphopenia was defined as less than 1 billion lymphocytes per liter. Low C3 and C4 were defined as less than 0.8 g/L and 0.2 g/L, respectively. Significant associations between categorical variables and endotype (denoted with asterisks) were identified using Chi-square Test of Independence. In (B) – (E), odds ratios of X1 having a positive value for the clinical trait of interest are displayed above the X1 bar with significance indicated by asterisks. Missing data (n.d.) were excluded from analyses. Graphs were created in GraphPad Prism v 9.1.0 (221). ESR=erythrocyte sedimentation rate. n.d.=no data. HCQ=hydroxychloroquine. MMF=mycophenolate mofetil. LN=lupus nephritis. *p<0.05; **p<0.01; ***p<0.001; ****p<0.0001.

A

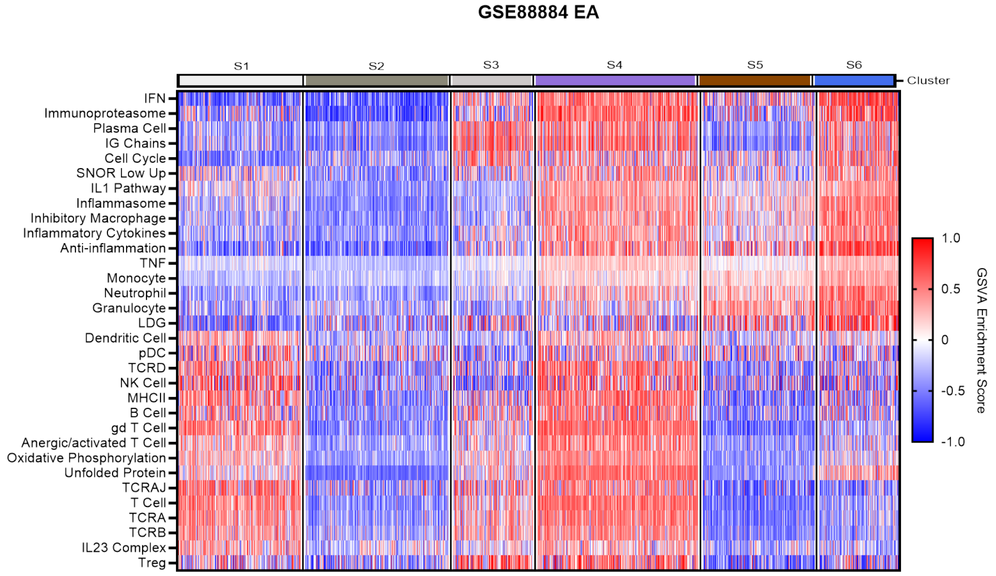

B

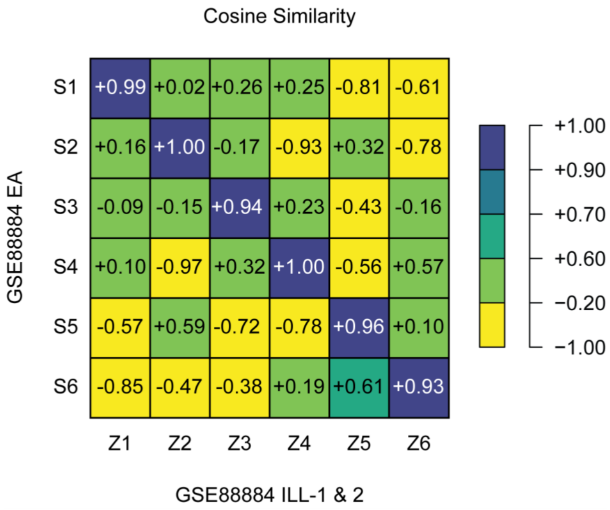

Figure S31. Endotypes of EA adult SLE patients.
(A) The k-means clustering pipeline applied to 1,118 baseline active lupus samples from GSE88884 (ILL-1 & ILL-2) of European ancestry identified six endotypes. (B) The identified clusters were compared to the six endotypes identified using all 1,620 active lupus samples by cosine similarity. Heatmap in (A) was generated with GraphPad Prism v. 9.5.0 (730). The cosine similarity plot in (B) was generated in R with the plot.matrix package and edited in Adobe Illustrator.

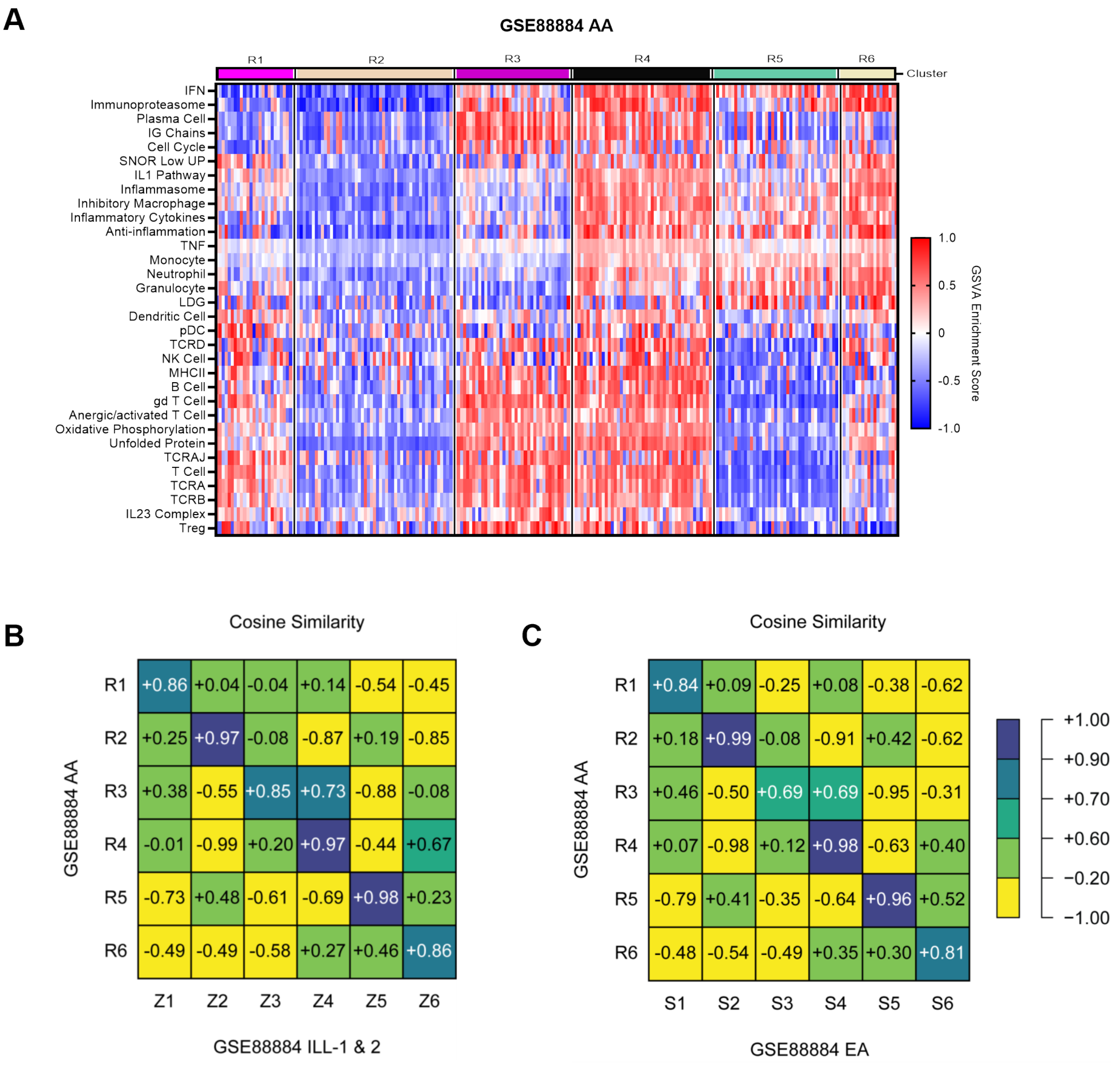

Figure S32. Endotypes of AA adult SLE patients.
(A) The k-means clustering pipeline applied to 216 baseline active lupus samples from GSE88884 (ILL-1 & ILL-2) of African ancestry identified six endotypes. The identified clusters were compared to (B) the six endotypes identified using all 1,620 active lupus samples and (C) the six EA patient endotypes by cosine similarity. Heatmap in (A) was generated GraphPad Prism v. 9.5.0 (730). The cosine similarity plots in (B-C) were generated in R with the plot.matrix package and edited in Adobe Illustrator.

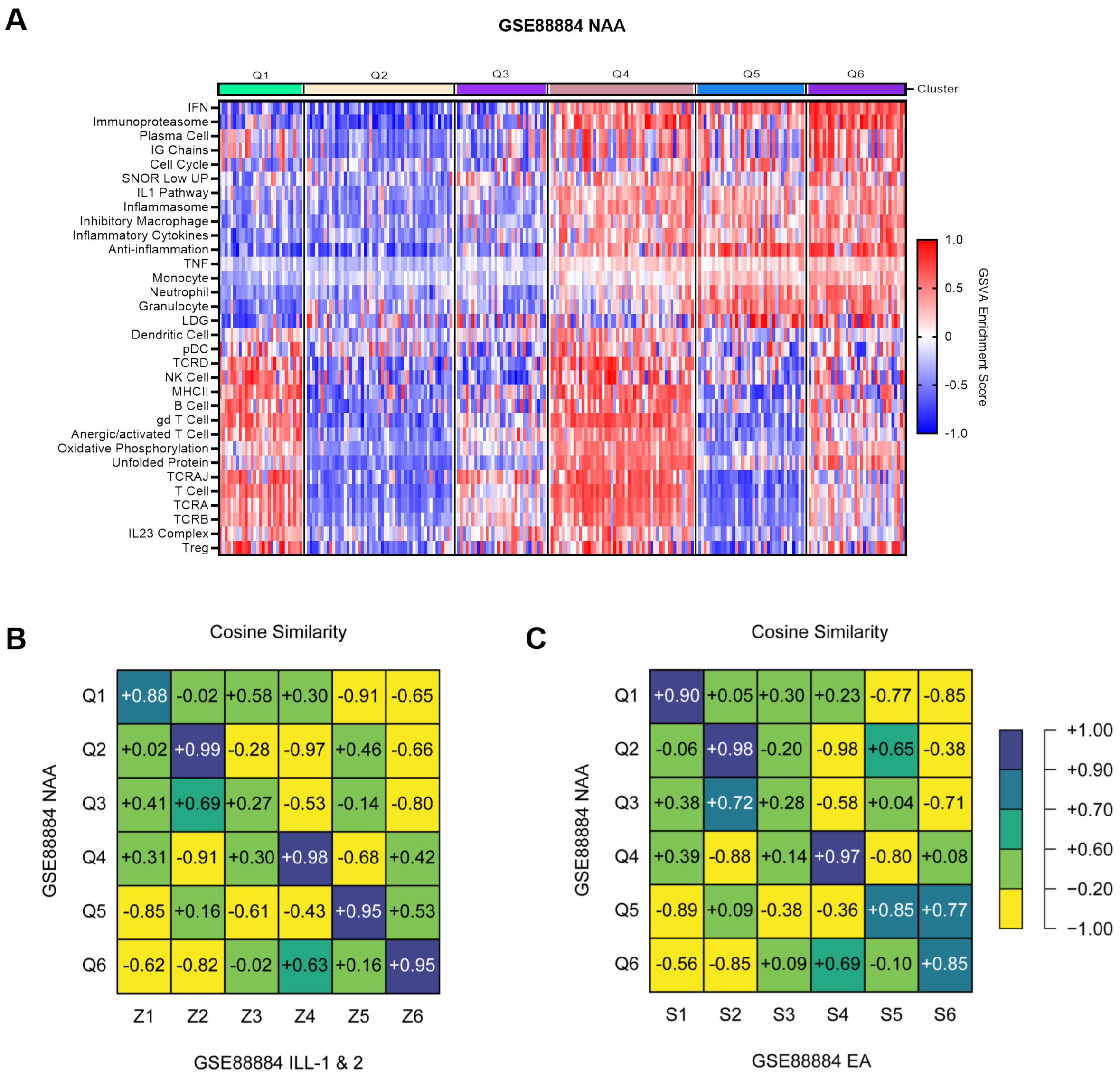

Figure S33. Endotypes of NAA adult SLE patients.
(A) The k-means clustering pipeline applied to 232 baseline active lupus samples from GSE88884 ILL-1 & ILL-2 of Native American (Hispanic) ancestry identified six endotypes. The identified clusters were compared to (B) the six endotypes identified using all 1,620 active lupus samples and (C) the six EA patient endotypes (C) by cosine similarity. Heatmap in (A) was generated with GraphPad Prism v. 9.5.0 (730). The cosine similarity plots in (B-C) were generated in R with the plot.matrix package and edited in Adobe Illustrator.

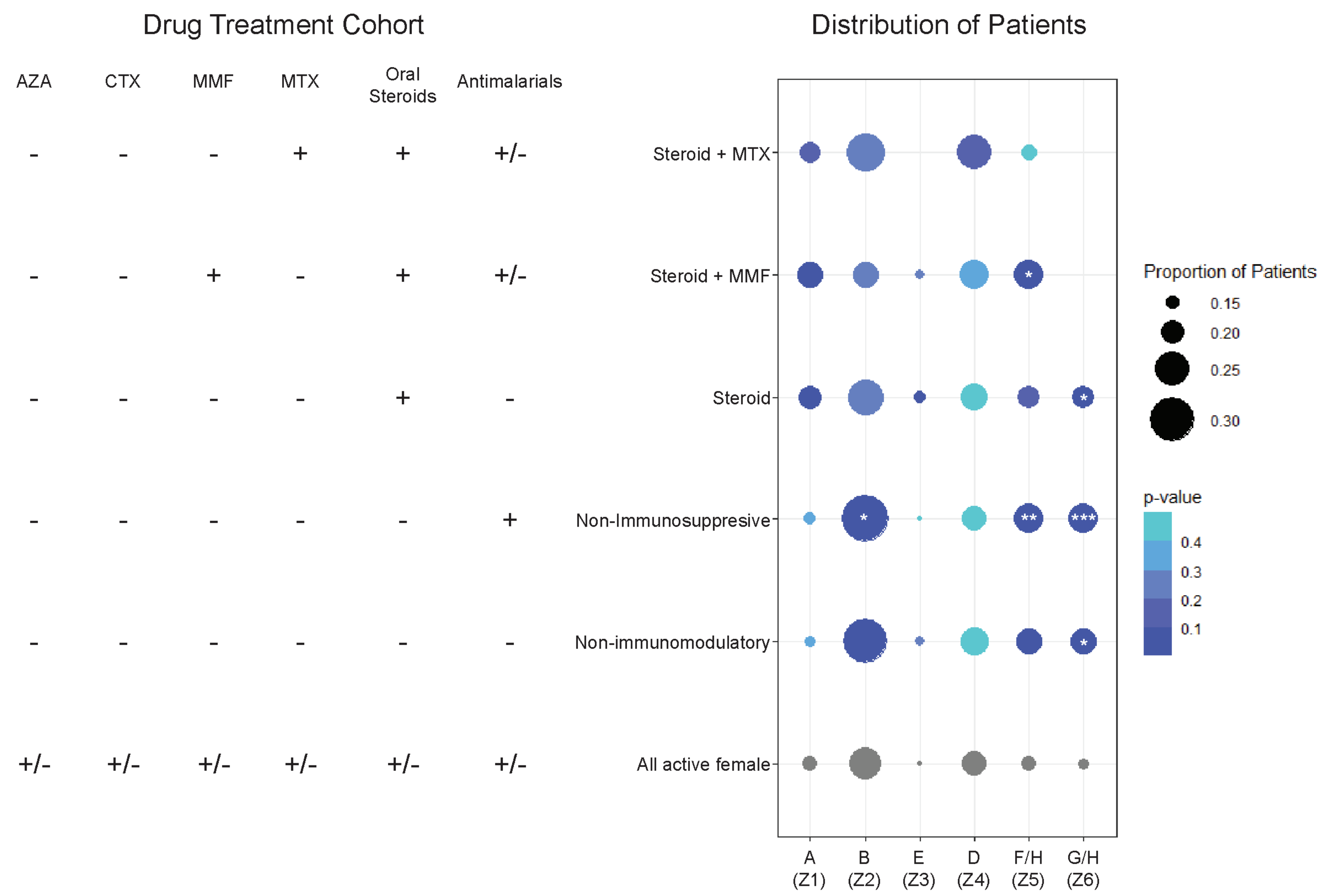

Figure S34. Distribution of patients in GSE88884 drug treatment cohorts.
Active, female SLE patients in GSE88884 ILL-1 & ILL-2 were stratified by lupus SoC at baseline into drug treatment cohorts. Each cohort was individually endotyped with k=5 subsets per Elbow and Silhouette analyses. Resultant endotypes were then compared to the six endotypes identified with all 1,620 active, female SLE patients in GSE88884 using cosine similarity. The proportion of patients in each endotype with cosine similarity > 0.7 are displayed. The absence of a data point indicates that no endotype in the drug treatment cohort was similar to the reference endotype using this cosine similarity cutoff. Significant deviations in the proportion of patients from the full cohort (gray) are denoted with black asterisks. Significance was determined using the prop_test function in R and the bubbleplot was generated with the ggplot2 package. AZA=azathioprine; CTX=Cytoxan (cyclophosphamide); MMF=mycophenolate mofetil; MTX=methotrexate. *p<0.05; **p<0.01; ***p<0.001.

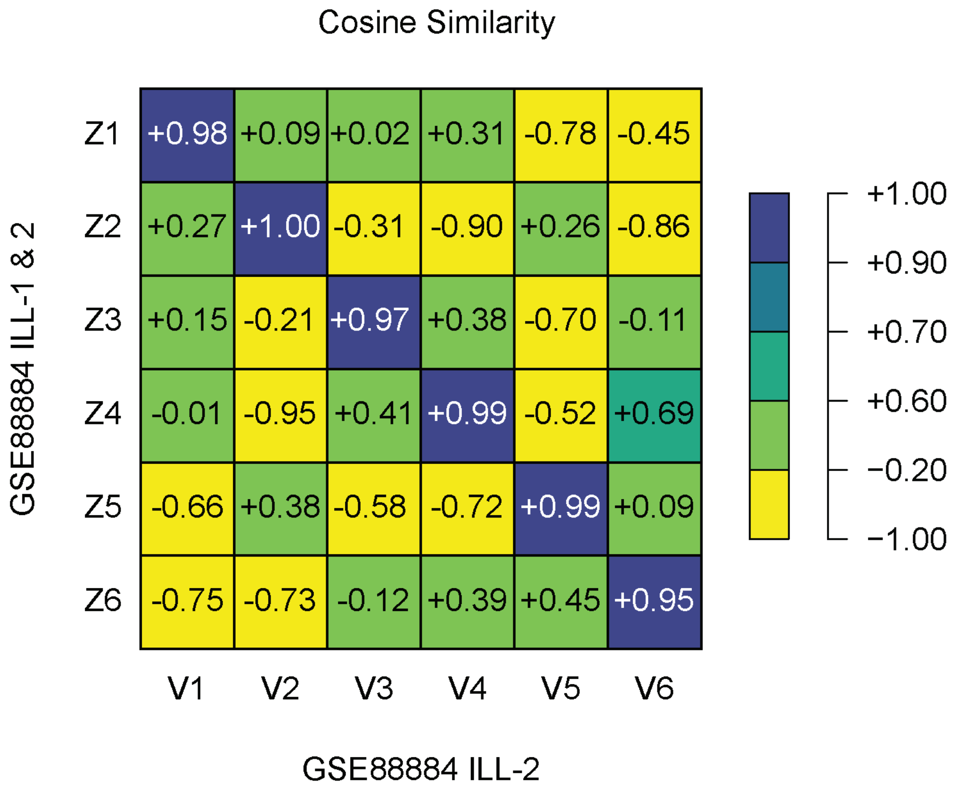

Figure S35. Endotypes in GSE88884 ILL-2.
Cosine similarity of the identified endotypes in each GSE88884 ILL-1 & ILL-2 (n=1,620) and GSE88884 ILL-2 (n=807). The cosine similarity plot was generated with the plot.matrix R package and edited in Adobe Illustrator.

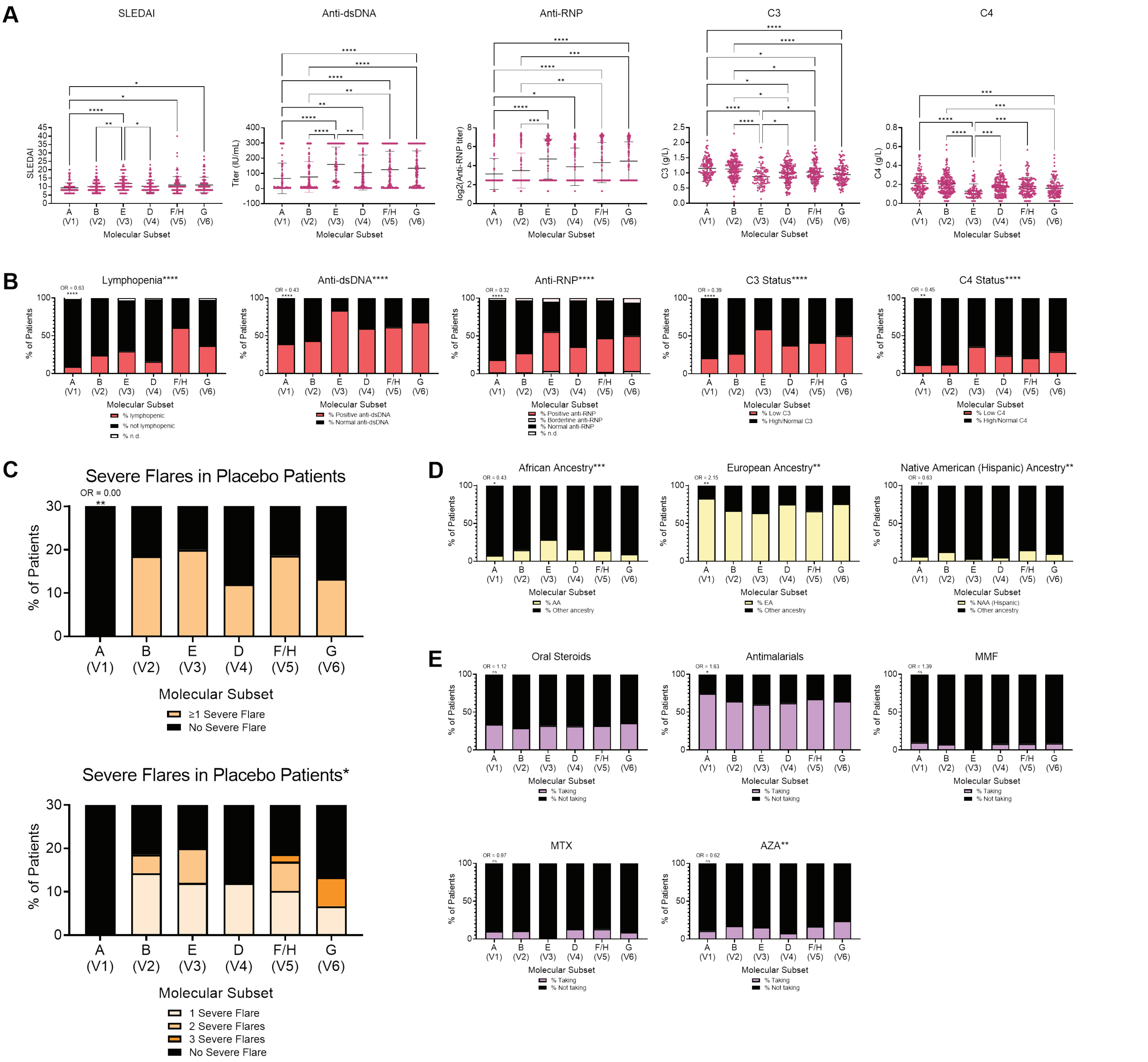

Figure S36. Clinical characterization of SLE endotypes in GSE88884 ILL-2.
Clinical metadata were summarized for each cluster from GSE88884 ILL-2 using baseline values. Metadata was categorized by (A) quantitative immunologic/inflammatory and systemic disease indicators, (B) categorical immunologic/inflammatory disease indicators, (C) incidence of subsequent flares over 52 weeks in placebo patients receiving SoC medication (n=274) (Table S7, and clusters were relabeled as one of the eight endotypes using cosine similarity. Scatterplots in (B) display the mean±SD for each endotype; statistical differences were found with Dunn's multiple comparisons test. Lymphopenia was defined as less than 1 billion lymphocytes per liter. Significant associations between categorical variables and endotypes (denoted with asterisks in titles) were identified using Chi Square Test of Independence. In (B)-(E), odds ratios of V1 having a positive value for the clinical trait of interest as compared to the other cohorts combined are displayed above the V1 bar with significance indicated by asterisks. Missing data (n.d.) were excluded from analyses. Graphs in (A)-(E) were created in GraphPad Prism v. 9.4.0 (673). n.d. = no data. MMF = mycophenolate mofetil. AZA = azathioprine. MTX = methotrexate.*p<0.05; **p<0.01; ***p<0.001; ****p<0.0001.
